# Genetic overlap and causality between depression and preterm birth: a large-scale genome-wide cross-trait analysis

**DOI:** 10.1017/S0033291725100718

**Published:** 2025-09-25

**Authors:** Min Zhang, Xinzhen Chen, Wenzheng Zhou, Niya Zhou, Cuihua Zhang, Yunping Yang, Qiyin Li, Xin Ming, Yifu Wu, Hongbo Qi, Wei Zhou

**Affiliations:** 1 Department of Sleep and Psychology, Women and Children’s Hospital of Chongqing Medical University, Chongqing Health Center for Women and Children, Chongqing, China; 2 Clinical and Public Health Research Center, Women and Children’s Hospital of Chongqing Medical University, Chongqing Health Center for Women and Children, Chongqing, China; 3 Chongqing Research Center for Prevention & Control of Maternal and Child Diseases and Public Health, Chongqing, China; 4 Department of Quality Control, Women and Children’s Hospital of Chongqing Medical University, Chongqing Health Center for Women and Children, Chongqing, China; 5 Department of Obstetrics and Gynecology, Women and Children’s Hospital of Chongqing Medical University, Chongqing Health Center for Women and Children, Chongqing, China

**Keywords:** bipolar disorder, depression, genome-wide cross-trait analysis, major depression, preterm birth, shared genetic architecture

## Abstract

**Background:**

Little is known regarding the shared genetic architecture underlying the phenotypic associations between depression and preterm birth (PTB). We aim to investigate the genetic overlap and causality of depression with PTB.

**Methods:**

Leveraging summary statistics from the largest genome-wide association studies for broad depression (N_total_ = 807,533), major depression (N_total_ = 173,005), bipolar disorder (N_total_ = 414,466), and PTB (N_total_ = 226,330), we conducted a large-scale genome-wide cross-trait analysis to assess global and local genetic correlations, identify pleiotropic loci, and infer potential causal relationships

**Results:**

Positive genetic correlations were observed between PTB and broad depression (*r_g_* = 0.242), major depression (*r_g_* = 0.236), and bipolar disorder (*r_g_* = 0.133) using the linkage disequilibrium score regression, which were further verified by the genetic covariance analyzer. Local genetic correlation was identified at chromosome 11q22.3 (harbors *NCAM1-TTC12-ANKK1-DRD2*) for PTB with depression. Cross-trait meta-analysis identified two loci shared between PTB and broad depression, two loci shared with major depression, and five loci shared with bipolar disorder, among which three were novel (rs7813444, rs3132948 and rs9273363). Mendelian randomization demonstrated a significantly increased risk of PTB for genetic liability to broad depression (odds ratio [OR]=1.30; 95% confidence interval [CI]: 1.11-1.52) and major depression (OR=1.27; 95%CI: 1.08-1.49), and the estimates remained significant across the sensitivity analyses.

**Conclusions:**

Our findings demonstrate an intrinsic link underlying depression and PTB and shed novel light on the biological mechanisms, highlighting an important role of early screening and effective intervention of depression in PTB prevention, and may provide novel treatment strategies for both diseases.

## Introduction

Individuals suffering from depression, especially among women of reproductive age, are frequently at an elevated risk of various subsequent health issues (Faravelli *et al.*, 2013). Depressive episodes that occur during pregnancy have devastating effects, including preterm birth (PTB), which is the leading cause of perinatal morbidity and mortality with a global prevalence of 11.1% (da Fonseca *et al.*, 2020; Miller *et al.*, 2022; Pearlstein, 2015). Hypothesized biological mechanisms underlying the observed association between depression and PTB include dysregulation in the hypothalamic–pituitary–adrenal axis and the immune system (Miller *et al.*, 2022). Yet, observational evidence concerning the association between depression and PTB remains inconclusive. Despite a meta-analysis of 26 studies involving 402,375 individuals suggested a significantly increased risk of PTB among depression patients [odds ratio (OR): 1.20; 95% confidence interval (CI): 1.10–1.40], the effect attenuated to null when restricted to women without antidepressant use (OR: 1.20; 95%CI: 0.90–1.70) (Vlenterie *et al.*, 2021). Inversely, a longitudinal study of 1,824 individuals with a follow-up of approximately 30 years suggested a notably elevated risk of depression among those who experienced preterm delivery (OR: 2.88; 95%CI: 1.15–7.22) (Loret de Mola *et al.*, 2014). These evidence indicate phenotypic associations derived from traditional epidemiological research may be influenced by bias, confounders, and reverse causality owing to their observational nature.

To address these contradictory findings, one approach is to explore the genetic basis of co-morbid conditions. Twin studies or family studies have demonstrated the genetic component in both depression and PTB, with heritability estimates of around 35.0% (Otte *et al.*, 2016) and 15.0% (Wadon *et al.*, 2020), respectively. Recent extensive genome-wide studies have suggested substantial genetic links between depression and several female reproductive characteristics, such as age at menarche and age of first childbirth, both of which are risk factors for PTB (Howard *et al.*, 2019; Wray *et al.*, 2018). Candidate gene-based studies have further identified multiple loci that influence both traits [i.e., *IL-1β*, *TNF-α*, *IL-6* (Bufalino *et al.*, 2013, Moore *et al.*, 2004), and *DEFB1* (Athreya *et al.*, 2019, Strauss *et al.*, 2018)]. These findings imply a potential shared genetic foundation between depression and PTB; however, the precise nature and scope of these associations remain ambiguous.

Recent progress in statistical genetics and genome-wide association studies (GWASs) has introduced various methods for conducting comprehensive genome-wide cross-trait analysis. This strategy is particularly useful in disentangling associations between complex traits that are difficult to investigate through observational studies due to confounding or reverse causation (Zhu *et al.*, 2021). First, genetic correlation analysis (both global and local) helps identify whether two traits share common genetic influences, which suggests a biological rather than purely environmental connection. Second, cross-trait meta-analysis allows for the discovery of pleiotropic genetic loci that simultaneously influence both traits, providing clues about shared molecular pathways. Third, Mendelian randomization (MR) enables causal inference by using genetic variants as instrumental variables, thereby minimizing confounding and establishing the directionality of effect. Together, these approaches allow us to investigate the genetic basis of the observed phenotypic association between depression and preterm birth in a rigorous and unbiased manner. However, as far as we understand, no genome-wide cross-trait analysis has been performed to investigate the common and unique etiological factors underlying depression and PTB.

**Figure 1.**
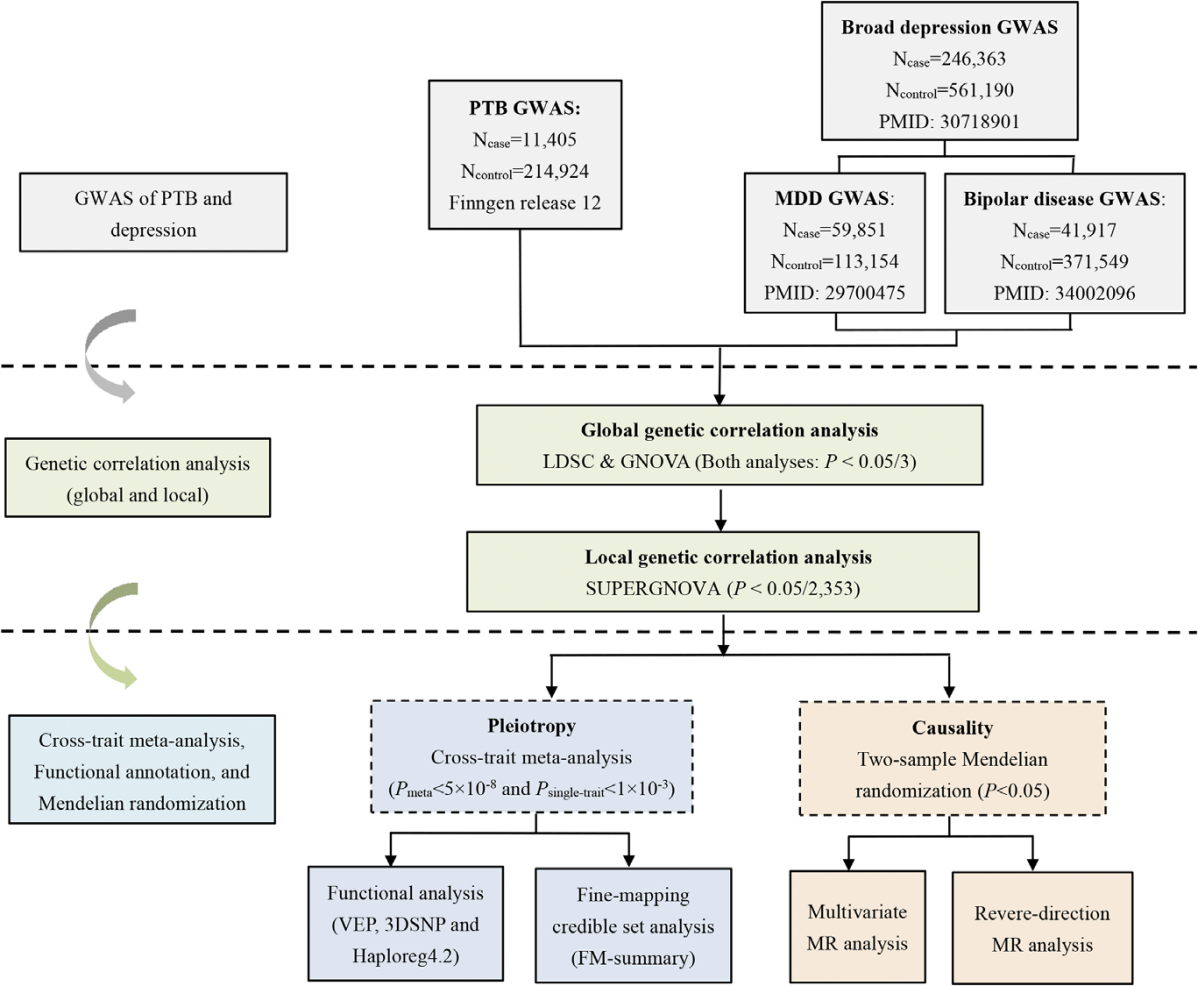
Overall study design of the genome-wide cross-trait analysis. GWAS summary statistics for each trait of interest were retrieved from publicly available GWAS(s). Global and local genetic correlation analyses between PTB and depression were conducted. Cross-trait meta-analysis was applied to identify pleiotropic loci and a bidirectional two-sample Mendelian randomization analysis was used to infer putative causal relationship. Note: PTB, ‘preterm birth’; MDD, ‘major depressive disorder’; GWAS, ‘genome-wide association study’.

Thus, in this study, we conducted a comprehensive genome-wide cross-trait analysis to systematically evaluate the genetic overlap and causality between PTB and depression, as well as its related phenotypes, major depression (MDD) and bipolar disorder (BIPD). The overall design of this large-scale genome-wide cross-trait analysis is illustrated in Figure 1.

## Methods and materials

### Data sources of PTB and depression

This study is a secondary analysis of existing GWASs. All the summary statistics were obtained from publicly available GWASs conducted for PTB, broad depression, MDD, and BIPD. Comprehensive details on the characteristics of each included data source are presented in Supplementary Table S1.

### Broad depression

GWAS summary statistics for broad depression (Howard *et al.*, 2019) was sourced from a meta-analysis involving 807,553 participants of European ancestry (with 246,363 depressive cases, 561,190 healthy controls), which meta-analyzed data from the three biggest genetic researches of depression including UK Biobank, Psychiatric Genomics Consortium (PGC), and 23andMe (Supplementary Table S1). In this GWAS, the confirmation of depression was based on self-reports from individuals who had experienced a diagnosis or treatment due to clinical depressive symptoms (30.7%), having seen a general practitioner or psychiatrist due to anxiety, neurological, tension, or depressive symptoms (51.8%), or clinically-derived phenotypes for MDD (17.5%). Independent genome-wide significant SNPs were identified at a *P*-threshold of 5.0 × 10^−8^. In total, 97 broad-depression associated index variants were associated with the outcome and subsequently utilized as genetic instrumental variables (IVs). The relevant information was extracted. The relevant information on broad depression IVs is shown in Supplementary Table S2.

### Major depressive disorder

GWAS summary data for MDD (Wray *et al.*, 2018) was sourced from a meta-analysis of seven European-ancestry cohorts including PGC29, 23andMe, deCODE, GenScotland, GERA, iPSYCH, and UK Biobank comprising 173,005 participants (with 59,851 MDD cases, 113,154 healthy controls) (Supplementary Table S1). Cases were diagnosed with MDD according to the international consensus criteria (DSM-IV, ICD-9, or ICD-10) through structured diagnostic instruments, interviews conducted by trained professionals, clinician-administered checklists, or review of medical records. The MDD cases excluded patients with lifetime BIPD or schizophrenia. In total, 40 independent MDD-associated genome-wide significant (5.0 × 10^−8^) index variants were identified as associating with the outcome, and subsequently used as genetic IVs for MDD in the final MR analyses (Supplementary Table S3).

### Bipolar disorder

GWAS summary data for BIPD (also known as manic depression) (Mullins *et al.*, 2021) was obtained from a meta-analysis involving 57 cohorts from Australia, Europe, and North America comprising 414,466 individuals of European descent (with 41,917 BIPD cases, 371,549 healthy controls) (Supplementary Table S1). Cases were diagnosed with BIPD according to the international consensus criteria (DSM-IV, ICD-9, or ICD-10) through structured diagnostic instruments, interviews conducted by trained professionals, clinician-administered checklists, or review of medical records. Sixty-four independently BIPD-associated genome-wide significant (5.0 × 10^−8^) index variants were identified associating with the outcome and subsequently used as genetic IVs for BIPD in the final MR analysis (Supplementary Table S4).

### Preterm birth

GWAS summary data for PTB was obtained from FinnGen release 12 (https://finngen.gitbook.io/documentation/data-download), consisting of 226,330 individuals (11,405 PTB cases and 214,924 controls) (Supplementary Table S1). Preterm deliveries are those that occur at less than 37 weeks of gestational age for pregnant women (Kurki *et al.*, 2023). A total of 118 independent index variants were obtained associating with the outcome with a *P*-threshold of 5.0 × 10^−5^ (clumped in EUR with r^2^ = 0.001 and kb = 10000) and subsequently used as genetic IVs as no genome-wide significant variants (5.0 × 10^−8^) were identified. The relevant information on PTB-associated IVs is shown in Supplementary Table S5.

## Statistical analysis

### Global genetic correlation analysis

To quantify the genome-wide genetic correlations across PTB and broad depression (as well as MDD and BIPD), which are not biased by environmental factors, we employed the methods of linkage disequilibrium score regression (LDSC) (Bulik-Sullivan *et al.*, 2015) and genetic covariance analyzer (GNOVA) (Lu *et al.*, 2017a). The global genetic correlation coefficients (represented by 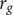 ) range between −1 and + 1, where −1 represents an entirely negative correlation, and + 1 represents a wholly positive correlation. In conducting these analyses, we applied a Bonferroni-adjusted *P*-value (*P* < 0.017 = 0.05/3, number of depression-related symptoms) to establish statistical significance.

### Local genetic correlation analyses

Specific genetic variants within a particular genomic area also play a role in linking two traits. We further investigated the local genetic correlation using the SUPERGNOVA tool (Zhang *et al.*, 2021b). This method divides the entire genome into about 2,353 linkage disequilibrium (LD)-independent segments, offering an accurate measure of the genetic similarity across trait pairs influenced by genetic loci at each segment. To establish statistical significance, we used a Bonferroni-adjusted *P*-value threshold (*P* < 2.12 × 10^−5^ = 0.05/2,353 number of LD-independent segments).

### Cross-trait meta-analysis

Using the cross-phenotype association (CPASSOC) (Li and Zhu, 2017), a cross-trait meta-analysis was performed to pinpoint pleiotropic loci that influence both PTB and broad depression, as well as its two subtypes. CPASSOC amalgamates multiple traits’ association evidence from various GWAS summary statistics, thereby uncovering cross-phenotype connections. It is equipped to conduct both S_Het_ and S_Hom_ tests. The definitions of S_Hom_ and S_Het_ are listed in Supplementary Materials. Briefly, S_Hom_ can be viewed as a fixed-effect inverse variance weighted meta-analysis. S_Hom_ assumes that genetic effects are homogeneous and is suitable when the effect size is the same for all traits and populations. S_Het_ is an extension of S_Hom_, which allows for varying effect sizes and directions across traits and cohorts. S_Het_ addresses heterogeneity by introducing a truncated signed-weight statistic that assigns more weight to larger effect values for specific traits. As S_Het_ is more robust to heterogeneity, we adopted S_Het_ rather than S_Hom_ in our analyses.

Subsequent to CPASSOC analysis, PLINK’s clumping function was utilized to isolate independent shared loci for both traits, by using settings like ‘--clump-p1 5e-8 --clump-p2 1e-5 --clump-r2 0.2 --clump-kb 1000’. A variant was deemed significantly pleiotropic if it had a *P*_single-trait_ of less than 1.0 × 10^−3^ for both traits and a *P*_CPASSOC_ of less than 5.0 × 10^−8^.

Every substantial pleiotropic SNP was categorized into one of the four distinct groups. Initially, a ‘known’ SNP is characterized as one having a *P*_single-trait_ of less than 5.0 × 10^−8^, shared among both traits. Next, a ‘single-trait-driven’ SNP is one where the *P*_single-trait_ is less than 5.0 × 10^−8^ for just one out of the two traits. Third, an ‘LD-tagged’ SNP is described as a variant linked through linkage disequilibrium (LD) to index SNPs pinpointed by single-trait GWAS. (LD *r*^2^ > 0.1). Lastly, a novel SNP is identified as one that is not influenced by any individual trait and does not correlate through LD with any index SNPs pinpointed by single-trait GWAS.

### Functional annotation

To elucidate the biological significance of the shared SNPs discovered through CPASSOC, we linked these SNPs with specific genes and performed functional annotation using the variant effect predictor (VEP) (McLaren *et al.*, 2016), Haploreg4.2 (Ward and Kellis, 2012), and 3DSNP (Lu *et al.*, 2017b). VEP and Haploreg4.2 identify potential genes by considering their physical closeness to the SNPs, whereas 3DSNP examines the regulatory role of variants by analyzing their three-dimensional chromatin interactions with the genes mediated through chromatin loops.

### Fine-mapping credible set analysis

We subsequently pinpointed a 99% credible set of causal loci using the FM-summary tool which is a streamlined Bayesian fine-mapping algorithm, given that an index SNP might not directly indicate causal loci (Schaid *et al.*, 2018). In summary, for each of the nine variants pinpointed collectively by CPASSOC, we extracted variants within a 1000 kb radius of the index SNP to serve as input for the FM-summary. This method primarily detects the chief signal and employs a flat prior alongside the steepest descent approximation, thereby generating a posterior inclusion probability (PIP) for each variant. The 99% credible set of loci is determined by ordering the variants based on descending PIPs and accumulating the PIPs until reaching at least 99%.

### Mendelian randomization analyses

To make a causal inference, a two-sample MR was finally conducted using specific packages in R with version 4.1.2 (Burgess *et al.*, 2015). We calculated *R*^2^ to estimate the variance proportion in an ‘exposure’ interpreted by the genetic IVs and calculated *F*-statistics to assess these genetic IVs’ strength. The statistical power for MR was calculated using an online calculator (https://sb452.shinyapps.io/power/). We employed the inverse-variance weighted (IVW) model as the main method. Additionally, we employed the MR-Egger regression and weighted median models to affirm the credibility of our findings, accommodating less stringent model assumptions (Bowden *et al.*, 2015). We set a *P*-value of less than 0.05 to define statistical significance.

Additional sensitivity analyses were carried out to verify the credibility of our MR findings. Initially, genetic IVs that were palindromic – where alleles match on both the forward and the reverse strands – were omitted. Then, a leave-one-out analysis was performed by sequentially removing each SNP, employing the IVW model with the remaining SNPs. Additionally, the MR-Pleiotropy Residual Sum and Outlier (MR-PRESSO) tool was applied to assess horizontal pleiotropy and revise the causal estimates after outlier exclusion (Verbanck *et al.*, 2018). Fourth, a reverse-directional MR was also performed to explore the potential causal impact of PTB on depression with ruling out reverse causality. Horizontal pleiotropy and heterogeneity were further assessed using the MR-Egger intercept and Cochran’s Q test, respectively, with significance noted for *P*-values less than 0.05 (Bowden *et al.*, 2018). Lastly, multivariate MR (MVMR) was applied to incorporate SNP associations with multiple phenotypes in a single model, addressing the influence of major confounding factors (Burgess and Thompson, 2015), such as antidepressants (Wu *et al.*, 2019), body mass index (BMI) (Pulit *et al.*, 2019), type 2 diabetes mellitus (T2DM) (Mahajan *et al.*, 2018), current smoking (Liu *et al.*, 2019), alcohol consumption per day (Liu *et al.*, 2019), and sleep duration (Dashti *et al.*, 2019).

## Results

### Global and local genetic correlation

Using the method of LDSC (Table 1), we found a notable global genetic correlation between broad depression and PTB ( 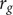 : 0.242, *P*: 2.89 × 10^−07^), and this genetic correlation estimated by the GNOVA method was consistent in direction, although the effect size was about half smaller ( 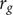 : 0.147, *P*: 3.35 × 10^−05^). As to the subtypes, similar significant genetic correlations were observed for both MDD ( 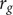 : 0.236, *P*: 2.89 × 10^−07^) and BIPD ( 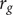 : 0.133, *P*: 5.80 × 10^−03^) with PTB by using LDSC. These estimates of MDD-PTB ( 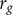 : 0.150, *P*: 4.06 × 10^−04^) and BIPD-PTB ( 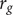 : 0.086, *P*: 1.45 × 10^−02^) remained significant in GNOVA. All the estimates withstood Bonferroni correction (*P* < 1.67 × 10^−2^).

**Table 1.** Global genetic correlations between preterm birth and depression based on LDSC and GNOVA

Trait 1	Trait 2	LDSC			GNOVA	
		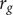	se	*P*	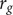	*P*
PTB	Broad Depression	0.242	0.047	2.89 × 10^−07^	0.147	3.35 × 10^−05^
	Major Depression	0.236	0.061	1.00 × 10^−04^	0.150	4.06 × 10^−04^
	Bipolar Disorder	0.133	0.048	5.80 × 10^−03^	0.086	1.45 × 10^−02^

*Note:* 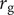 , genetic correlation; SE, standard error; PTB, preterm birth.

**Figure 2.**
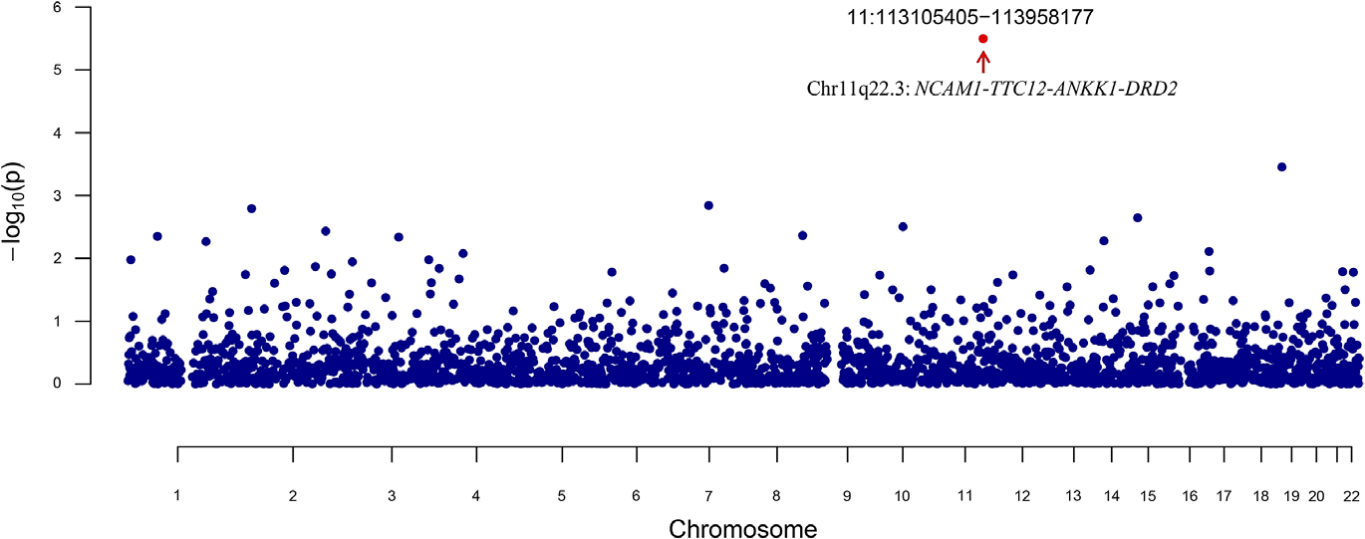
Local genetic correlation between preterm birth and broad depression identified by SUPERGNOVA.

When segmenting the entire genome into LD-independent areas (2,353 blocks), a single notable region was pinpointed for broad depression and PTB at 11q22.3 (chromosome 11:113105405–113958177) with a *P* value of 9.58 × 10^−07^, which harbors *NCAM1-TTC12-ANKK1-DRD2* gene cluster (Figure 2). In addition, a significant local signal was observed for BIPD and PTB at 22q13.2 (chromosome 43,187,900-43,645,335) with a *P* value of 3.81 × 10^−08^. No significant local genetic correlations were identified between MDD and PTB in any genetic regions.

### Cross-trait meta-analysis

As shown in Table 2, a total of nine SNPs were pinpointed that were shared between depression-related traits and PTB, among which two loci were shared between broad depression and PTB (rs2734837 and rs13220522), two loci were shared between MDD and PTB (rs149543464 and rs57440165), and five loci were shared between BIPD and PTB (rs3132948, rs1264349, rs7813444, rs9273363, and rs60476972). Notably, these loci had not been previously identified at genome-wide significance for PTB, although most were associated with depression-related symptoms (five out of nine loci) which were classified as ‘single-trait-driven’ shared loci. Interestingly, the single-trait-driven variant, rs2734837, shared between broad depression and PTB, is mapped to chromosome 11q22.3 (*DRD2* gene region), which is also consistent with the results of the local genetic analysis. In addition, rs13220522 (shared between broad depression and PTB) and rs57440165 (shared between MDD and PTB) were located in an LD block (*r*^2^: 0.86), which mapped to chromosome 6p22.2. This chromosome region contains a large histone gene cluster (e.g. *HIST1H1A*), a subset of the immunoglobulin gene superfamily clusters (e.g. *BTN2A2*), and other genes, which are related to neuronal development [e.g. *SCGN* (Liu *et al.*, 2023), *ABT1* (Oda *et al.*, 2000)] and innate immunity [e.g. *TRIM38* (Xue *et al.*, 2012), *BTN2A2* (Sarter *et al.*, 2016), *BTN3A1* (Payne *et al.*, 2020)]. Another two ‘single-trait-driven’ pleiotropic loci (rs149543464 and rs1264349) were also in an LD block (*r*^2^: 0.80). These variants mapped to *HLA-B*, a gene that also plays a central role in adaptive immunity (Di *et al.*, 2021).

**Table 2.** Genome-wide significant loci shared between preterm birth and depression (*P*_CPASSOC_ < 5 × 10^−8^, single trait *P*-value <1 × 10^−3^)

SNP	Chr:Position	A1/A2	*P.*cpassoc	Depression		Preterm birth		Genes within clumping area	Linear closest genes^a^	Interacting genes^b^	Class
				Beta	*P*	Beta	*P*				
*Preterm birth and broad depression*											
rs2734837	Chr11:1132868291	T/-C	2.23 × 10^−08^	−0.02	2.49 × 10^−08^	−0.04	2.34 × 10^−03^	ANKK1, DRD2, TTC12	DRD2	ANKK1, DRD2, MIR4301, TTC12	Single-trait
rs13220522	Chr6:25514179	A/G	6.15 × 10^−11^	−0.05	3.15 × 10^−11^	0.13	2.96 × 10^−04^	BTN2A2, HIST1H1A, SCGN, HFE, SLC17A1 and other 41	HIST1H4H	HIST1H1D and other 26	Single-trait
*Preterm birth and major depression*											
rs149543464	Chr6:302877773	A/G	3.91 × 10^−10^	−0.07	4.51 × 10^−09^	0.07	2.99 × 10^−03^	HLA-B, HLA-C and other 40	HLA-E	GNL1, HLA-E, PRR3	Single-trait
rs57440165	Chr6:25848025	A/C	6.67 × 10^−10^	0.08	2.52 × 10^−08^	0.08	7.40 × 10^−03^	ABT1, BTN1A1, HCG11, HFE, HIST1H1A, and other 59	GUSBP2	-	Single-trait
*Preterm birth and bipolar disorder*											
rs60476972	Chr16:9152663	G/A	3.98 × 10^−08^	−0.04	1.65 × 10^−05^	−0.05	7.62 × 10^−05^	–	RP11–473I1.6	C16orf72, USP7	LD-tagged
rs7813444	Chr8:65437506	A/G	4.01 × 10^−09^	0.04	3.55 × 10^−05^	0.06	8.41 × 10^−06^	BHLHE22, LOC401463	RP11–21C4.1	BHLHE22, LOC401463	Novel
rs3132948	Chr6:32191730	T/G	1.65 × 10^−08^	−0.05	1.92 × 10^−07^	0.05	2.90 × 10^−04^	NOTCH4		FKBPL and other 17	Novel
rs9273363	Chr6:32626272	C/A	5.76 × 10^−09^	0.05	3.94 × 10^−05^	0.08	3.77 × 10^−06^	–	HLA-DQB1	HLA-DQA1, HLA-DQA2, HLA-DQB1, HLA-DQB2	Novel
rs1264349	Chr6:30566407	A/G	2.72 × 10^−11^	0.10	4.30 × 10^−11^	−0.07	4.85 × 10^−03^	HLA-B, HLA-C and other 40	BTN2A1	FLOT1 and other 14	Single-trait

*Note*: A1/A2: effect-allele/other-allele.

a Linear closest genes of index SNP was mapped by VEP or Heploreg.

b 3D interacting genes of index SNP was mapped by 3DSNP.

In addition to ‘single-trait-driven’ pleiotropic SNPs, we identified three novel SNPs shared by BIPD and PTB, among which rs7813444 (*P*_CPASSOC_: 4.01×10^−9^) mapped to *BHLHE22*, rs9273363 (*P*_CPASSOC_: 5.79×10^−9^) mapped to *HLA-DQB1*, and rs3132948 (*P*_CPASSOC_: 1.65×10^−8^) mapped to *NOTCH4.*

### Functional annotation

To gain a potential biological understanding of the shared variants pinpointed by CPASSOC, functional annotations were conducted through VEP, Haploreg4.2, and 3DSNP. Functional annotation by Haploreg4.2 indicated that these shared SNPs fall within potential functional regions (Supplementary Table S6). For example, rs9273363, located in 971 bp 3′ of *HLA-DQB1*, overlaps with an enhancer activity cluster in five major tissue types, is bound by POL24H8 and four altered motifs, and is DNAse hypersensitive in two cell types (BLD, BLD). Functional analysis by VEP also showed that these shared SNPs have potential regulatory features (Supplementary Table S7). In addition, functional annotations by 3DSNP identified many genes that interact with the shared SNPs through 3D chromatin loops in different cell types (Supplementary Tables S8–S10), and many eQTLs that were significantly associated with the shared SNPs in 44 human tissues obtained from GTEx Portal (Supplementary Tables S11–S13). For example, for rs1264349, which was shared between BIPD and PTB, 15 three-dimensional interacting genes (*FLOT1* and other 14) in six tissues had been identified, among which *FLOT1* was identified as a risk gene for neurological diseases, such as depressive disorder (Zhan *et al.*, 2023) and also expressed in term villous placental cytotrophoblasts and endothelial cells (Walton *et al.*, 2013).

### Fine-mapping credible set analysis

Fine-mapping analyses by the method of FM-summary evaluated the 99% credible set of causal SNPs at each shared locus identified by CPASSOC, pinpointing targets for subsequent experimental investigations. The credible set variants for each locus related to the three depressive symptoms and PTB derived from this fine mapping are shown in Tables S14–S16. A total of 104 potential causal variants were pinpointed for broad depression and PTB (Supplementary Table S14), 135 potential causal variants were pinpointed for MDD and PTB (Supplementary Table S15), and 180 potential causal variants were pinpointed for BIPD and PTB (Supplementary Table S16).

### Mendelian randomization

At last, a two-sample MR analysis was conducted utilizing 89 depression-associated, 36 MDD-associated, and 56 BIPD-associated variants as genetic IVs to infer the causal relationships. *F*-statistics for these IVs were listed in Tables S2–S4 and all of the *F*-statistics were no less than 10, suggesting no existence of weak instruments.

**Figure 3.**
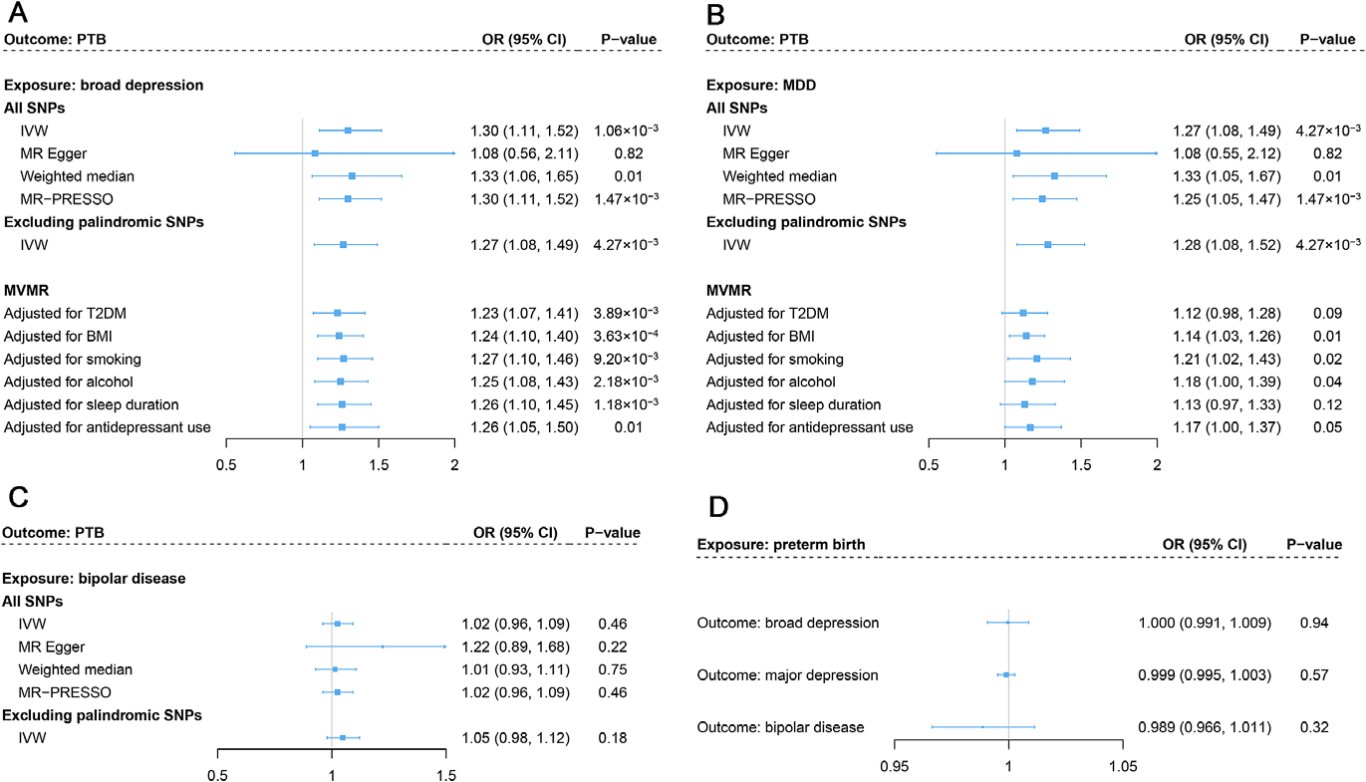
Bidirectional causal relationship between depression and preterm birth. Estimates represent causal effects for broad depression (a), major depression (b), and bipolar disease (c) with preterm birth. (d) Estimates of causal effects for preterm birth with depression and its subtypes. Note: PTB, ‘preterm birth’; MDD, ‘major depression’; BIPD, ‘bipolar disease’; IVW, ‘inverse-variance weighted’; T2DM, ‘type 2 diabetes mellitus’; BMI, ‘body mass index’.

As shown in Figure 3a, genetic predisposition to broad depression significantly correlated with an elevated PTB risk (OR_IVW_: 1.30, 95%CI: 1.11–1.52). This estimate did not alter in the weighted median model with an OR of 1.33 (95%CI: 1.06–1.65) and remained stable in the sensitivity analyses by excluding palindromic variants with an OR of 1.27 (95%CI: 1.08–1.49) and outliers in MR-PRESSO with an OR of 1.30 (95%CI: 1.11–1.52). No evidence of horizontal pleiotropy (*P* for MR-Egger intercept is 0.58) was detected. After adjusting for T2DM, BMI, smoking, alcohol, sleep duration, and antidepressant use in the MVMR, the effect also remained significant (Figure 3a).

As for the other two phenotypes, a similarly significant association was observed for MDD with PTB with an OR_IVW_ of 1.27 (95%CI: 1.08–1.49), as depicted in Figure 3b. This causality was supported further using the weighted median model with an OR of 1.33 (95%CI: 1.05–1.67), and sensitivity analyses after excluding palindromic SNPs (OR: 1.28, 95%CI: 1.08–1.52) and outliers (OR: 1.25, 95%CI: 1.05–1.47), and further adjustment for the potential confounders (Figure 3b). No causal impact of genetic predisposition to BIPD on the risk of PTB was pinpointed in the IVW model (OR: 1.02, 95%CI: 0.96–1.09), consistent across all the sensitivity analyses (Figure 3c). No evidence of horizontal pleiotropy (MDD: *P* for MR-Egger intercept is 0.49; BIPD: *P* for MR-Egger Q is 0.27) and heterogeneity (MDD: *P*_MR-Egger Q_ = 0.12, *P*_MR-Egger Q_ = 0.22) were detected.

No apparent influence of genetic liability to PTB was found on the risk of broad depression, MDD, or BIPD in the reverse directional MR analyses (Figure 3d).

## Discussion

As far as we understand, this genome-wide cross-trait analysis is the first one that comprehensively investigated the common genetic foundations underlying depression and PTB, supporting a substantial genetic link between PTB and both broad depression and its major subtypes. When the entire genome was divided into independent regions, a significant correlation at 11q22.3 was further identified. In addition, nine pleiotropic loci with joint associations with both PTB and depression were pinpointed using cross-trait meta-analysis. Finally, a causal role of depression and its major subtype on PTB risk was observed.

Through both LDSC and GNOVA, a strong global genetic similarity was pinpointed for broad depression and PTB, underscoring shared genetic biology between the two conditions. A pronounced local genetic correlation was also pinpointed at chromosome 11q22.3. This genetic region harbors a gene cluster *NCAM1-TTC12-ANKK1-DRD2* (also known as *NTAD* cluster), which was previously reported to be independently linked to depressive symptoms, neuropsychiatric disorders (Kimbrel *et al.*, 2023; Mota *et al.*, 2012, 2015) and brain function (Liu *et al.*, 2022; Petrovska *et al.*, 2017; Xu *et al.*, 2023). In addition, functional studies also suggested that the *NTAD* cluster had a potential role in pregnancy-related conditions. Silencing of the *NCAM1* gene as a potential therapeutic target in preeclampsia by suppressing oxidative stress and activating migration and invasion of umbilical vein endothelial cells (Zhang *et al.*, 2019). The *DRD2* gene regulates the decidualization of the human endometrial stromal cell (Bilibio *et al.*, 2015; Schoorlemmer *et al.*, 2020; Yu *et al.*, 2021). A significant signal at chromosome 22q13.2 was observed for BIPD and PTB. GWAS and functional genomics have revealed that genetic variants at the 22q13.2 risk locus (rs1801311 in *NDUFA6* gene) were robustly associated with schizophrenia (Li *et al.*, 2021, Schizophrenia Working Group of the Psychiatric Genomics, 2014). However, the specific mechanism of these genes in depression-related PTB needs to be further explored.

While our results suggest a potential shared biological etiology underlying depression and PTB, it could be due to pleiotropic impact (namely a loci influences both phenotypes) and/or causal impact (namely a loci influences one phenotype by its genetic effects on an intermediate phenotype). In the subsequent analyses aimed to explore these alternatives, a total of nine shared variants were pinpointed, among which two LD blocks (rs13220522-rs57440165 and rs149543464-rs1264349, both R^2^ > 0.80) were identified, suggesting the similarity of pathogenic mechanisms. These loci harbor genes that were previously linked to the development of nerve and the functions of the brain (*SCGN*, *ABT1*) (Liu *et al.*, 2023; Oda *et al.*, 2000), or biological processes related to the function of the placenta (*HLA-B*) (Hutter *et al.*, 1996). In addition, several pleiotropic variants were mapped to genes that play important roles in inflammatory responses and immune (e.g. *TRIM38*, *BTN2A2*, *BTN3A1*, and *HLA-B*) (Di *et al.*, 2021; Payne *et al.*, 2020; Sarter *et al.*, 2016; Xue *et al.*, 2012), which were significantly related to both depression and PTB. Thus, dysregulation of the immune system may serve as the biological basis for both the evolution of depression as well as its connection to PTB.

Through combining evidence of association from different studies, the meta-analysis of GWASs for multiple phenotypes can additionally uncover signals that are not detected as genome-wide significance in each single-phenotype analysis. Notably, three novel loci shared between BIPD and PTB (rs7813444 mapped to *BHLHE22*, rs9273363 mapped to *HLA-DQB1*, and rs3132948 mapped to *NOTCH4*) were identified. *NOTCH4* gene encodes a member of the NOTCH family of proteins, which play a role in vascular, especially brain arteriovenous malformation. In addition, convergent lines of evidence support that *NOTCH4* is a risk gene for schizophrenia (Zhang *et al.*, 2021a). Furthermore, altered *NOTCH4* in the human placenta is significantly inversely associated with low baby birth weight (Tiwari *et al.*, 2023). Further experimental researches were required for a more detailed functional annotation of these shared loci, especially concerning the onset of PTB and depression.

In addition to findings from the cross-trait meta-analyses (by CPASSOC) indicating biological pleiotropy (namely horizontal pleiotropy), results from MR analyses suggest causal relationships (namely vertical pleiotropy). Despite much epidemiological research regarding the association between depression and PTB had been performed, the findings remain inconclusive. For example, a previous meta-analysis consisting of 20 cohort studies which involved 29,295 participants showed a significantly increased risk of PTB among depression during pregnancy patients (ORs ranges: 1.01–4.90, pooled OR: 1.13; 95%CI: 1.06–1.21), however, this positive relationship attenuated to null when the study quality less than six (n = 5, pooled OR: 1.70; 95%CI: 0.99–2.92) and the effect size that without adjustment (n = 4, pooled OR: 1.46; 95%CI: 0.84–2.25) in the subgroup analyses (Grote *et al.*, 2010). A cohort study involving 7,267 individuals suggested pregnant women with depressive symptoms had a significantly evaluated risk of PTB (OR: 1.27, 95%CI: 1.04–1.55), however, those women who were treated for depression with an antidepressant medication did not (OR: 1.44, 95%CI: 0.86–2.43) (Venkatesh *et al.*, 2016), suggesting the positive association may be biased by confounding factors. We performed the first MR that provided evidence for a putative causal impact of depression on PTB, and this causal effect remained consistent when further adjusting for important confounders such as antidepressant use in the MVMR, indicating the independent role of depression in PTB. In the inverse direction, we did not observe a causal association between PTB and depression risk, which may be due to the selection of IVs for PTB (we set the threshold to 5.0 × 10^−5^). Further GWASs of PTB with extended study populations are warranted to verify the findings.

Our results hold significant implications for the field of public health. First, our research underscores the adverse impact of depression on PTB at the genetic level. Despite evidence-based guidelines for the prevention and treatment of depression or perinatal depression, they are often underutilized (Cox *et al.*, 2016; Force *et al.*, 2019; Siu *et al.*, 2016). This study further emphasizes that obstetric clinicians should pay more consideration to the screening, prevention, and treatment of perinatal depression. Second, the mechanism by which depression causes premature birth remains unknown. Our study identified several shared loci, providing a biological basis for further functional research into the pathogenesis of depression and PTB.

There are several limitations. First, all the results were limited to Europeans, limiting generalizability to other ethnicities. Second, it is hard to obtain publicly available women-specific GWASs statistics for depression with large sample size, which limits our analyses. We attempted to conducted sex-specific genetic analyses using sex-specific GWASs summary data of depression from the UK Biobank generated by the Neale Lab with 17,922 women cases and 9,358 men cases. The LDSC analysis suggested a potential genetic correlation between PTB and women-specific depression (*r*_g_ = 0.193, *P* = 0.056) but not men-specific depression (*r*_g_ = 0.184, *P* = 0.136) (Supplementary Table S17). In addition, MR analysis yielded a suggestive association between genetic liability to women-depression and increased risk of PTB (β = 2.92, *P* = 0.072) (Supplementary Table S18). These findings are consistent in direction with the primary analyses, although the potential reduction in statistical power due to the smaller sample size in the women-specific GWAS (17,922 cases versus 246,363 cases in the primary GWAS). Using adequately powered women-specific data in future research would be beneficial. Third, despite utilizing the hitherto largest GWAS for PTB, the case number remains relatively small, and future larger-scale GWASs are warranted to validate the results. Fourth, the lack of GWAS data for different PTB subclinical phenotype (spontaneous and medical-induced) precludes analysis of depression’s effects on these clinical subtypes, which warrants further clarification. Fifth, although shared loci (genes) were identified for depression and PTB, they rely on functional datasets and algorithms. Experimental studies are warranted to further uncover the physiopathological mechanisms. Finally, although we adjusted for antidepressant use in the MVMR, we acknowledge that medication-related confounding cannot be completely excluded. The GWAS summary statistics we used do not provide individual-level data on the timing, dosage, or duration of antidepressant exposure. Therefore, residual confounding due to unmeasured or misclassified medication use remains possible and may have influenced the observed associations. Future studies with individual-level data are needed to further validate and refine these findings.

In conclusion, this research advances our understanding of the phenotypic link between depression and PTB by providing genetic evidence of intrinsic correlation, disclosing shared genetic components, and drawing a causal inference between these two complex traits. Our findings highlight an intrinsic link between depression and PTB, shedding novel light on the physiological mechanisms and emphasizing the essential role of early screening and effective intervention of depression in PTB prevention, and may provide novel treatment strategies for both diseases.

## Supporting information

Zhang et al. supplementary material Zhang et al. supplementary material

## Supplementary material

The supplementary material for this article can be found at http://doi.org/10.1017/S0033291725100718.
