# Supplementary material for: Genetic overlap and causality between depression and preterm birth: a large-scale genome-wide cross-trait analysis
Source: Psychol Med. 2025 Sep 25;55:e279. doi: 10.1017/S0033291725100718 (PMC12527502; doi:10.1017/S0033291725100718)
Supplement: Zhang et al. supplementary material [file S0033291725100718sup001.docx]

## Supplementary Information to:

**Genetic overlap and causality between depression and preterm birth: a large-scale genome-wide cross-trait analysis**

Min Zhang et al.

Corresponding authors:

Hongbo Qi, E-mail: [qihongbo728@163.com](mailto:qihongbo728@163.com)

Wei Zhou, E-mail: [dr.zhouwei@163.com](mailto:dr.zhouwei@163.com)

**File Lists:**

**Supplementary Methods:** The definitions of S_Het_ and S_Hom_ in the CPASSOC.

**Table S1.** Details of the data sources used in this study.

**Table S2.** Characteristics of genetic instruments identified by GWAS to be associated with broad depression.

**Table S3.** Characteristics of genetic instruments identified by GWAS to be associated with major depression disorder.

**Table S4.** Characteristics of genetic instruments identified by GWAS to be associated with bipolar disorder.

**Table S5.** Characteristics of independent genetic instruments identified by Finngen to be associated with preterm birth.

**Table S6.** Functional annotation for the shared SNPs identified by CPASSOC through HeploReg V4.2.

**Table S7.** Detailed annotation of the shared SNPs identified from cross-trait meta-analysis through VEP.

**Table S8.** Genes that interact with the shared SNPs for broad depression and PTB through 3D chromatin loops in different cell types.

**Table S9.** Genes that interact with the shared SNPs for major depression and PTB through 3D chromatin loops in different cell types.

**Table S10.** Genes that interact with the shared SNPs for bipolar disease and PTB through 3D chromatin loops in different cell types.

**Table S11.** Significant SNP-gene pairs (FDR < 0.05) associated with the shared SNP for broad depression and PTB in 44 human tissues obtained from GTEx Portal.

**Table S12.** Significant SNP-gene pairs (FDR < 0.05) associated with the shared SNP for major depression and PTB in 44 human tissues obtained from GTEx Portal.

**Table S13.** Significant SNP-gene pairs (FDR < 0.05) associated with the shared SNP for bipolar disease and PTB in 44 human tissues obtained fromGTEx Portal.

**Table S14.** List of SNPs in the 99% credible set identified from fine-mapping analysis for each CPASSOC-identified locus shared between broad depression and PTB.

**Table S15.** List of SNPs in the 99% credible set identified from fine-mapping analysis for each CPASSOC-identified locus shared between major depression and PTB.

**Table S16.** List of SNPs in the 99% credible set identified from fine-mapping analysis for each CPASSOC-identified locus shared between bipolar disease and PTB.

**Table S17.** The results of LDSC using the gender-specific GWAS of UKB generated by the Neale Lab.

**Table S18.** The Mendelian randomization results of the association between female depression and the risk of preterm birth.

**Supplementary Methods:** The definitions of S_Het_ and S_Hom_ in the CPASSOC.

CPASSOC integrates association evidence from multiple correlated continuous and binary traits from one or multiple studies, and it provides two statistics, S_Hom_ and S_Het_ [PMID: 25500260]. S_Hom_ is defined as (assuming we have summary statistical results of GWASs from *J* cohorts with *K* phenotypic traits):


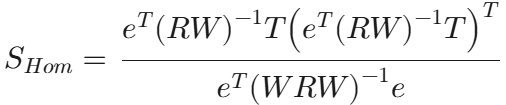


Where:

*T* is a vector of test statistics for the association of a SNP with *K* traits in *J* cohorts.

*R* is the correlation matrix of the test statistics.

*W* is a diagonal matrix of weights for the individual test statistics.

*e* is a vector of length *J*×*K* with all elements equal to 1.

When the statistics in *T* are mutually independent and *W* is diagonal with inverses of variances as elements, S_Hom_ is equivalent to an inverse variance weighted meta-analysis. S_Hom_ assumes that the genetic effect is homogeneous, meaning the effect size is the same across all traits and cohorts. However, when heterogeneous effects exist, in particular, if a variant contributes to only a subset of traits, this test is less powerful.

S_Het_ is an extension of S_Hom_, and is designed to integrate genetic associations while accounting for heterogeneity in effect sizes across multiple traits and cohorts. Unlike S_Hom_, S_Het_ allows for varying effect sizes and directions across traits and cohorts, making it more robust when heterogeneity is present. S_Het_ addresses heterogeneity by introducing a truncated signed-weight statistic that assigns more weight to larger effect values for specific traits.

S_Het_ is defined as:


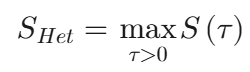


where


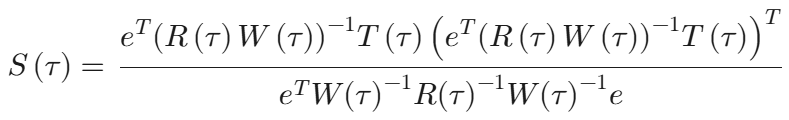


Here:

*T(τ)* is a subvector of the test statistics *T*, including only those with absolute values above a predefined threshold τ (τ >0).

*R(τ)* and *W(τ)* are submatrices corresponding to the selected test statistics.

*e* is a vector of ones.

**Table S1. Details of the data sources used in this study**

| **Factors** | **PMID** | **Sample size** | **Consortium** | **IVs** | **URLs** |
| --- | --- | --- | --- | --- | --- |
| Preterm birth | - | Total: 226,330  (11,405 case/214,924 controls) | Finngen R12 |  | [https://finngen.gitbook.io/documentation/data-download](https://finngen.gitbook.io/documentation/data-download) |
| Broad depression | 30718901 | Total: 807,553  (246,363 cases/561,190 controls) | PGC and UK Biobank (excluding 23andMe) | 97 | [https://datashare.is.ed.ac.uk/handle/10283/3203](https://datashare.is.ed.ac.uk/handle/10283/3203) |
| Major depression | 29700475 | Total: 173,005  (59,851 cases/113,154 controls) | PGC, deCODE, GenScotland, GERA, iPSYCH and UK Biobank | 40 | [https://www.ebi.ac.uk/gwas/studies/GCST005839](https://www.ebi.ac.uk/gwas/studies/GCST005839) |
| Bipolar disorder | 34002096 | Total: 414,466  (41,917 cases/371,549 controls) | 57 cohorts collected in Europe, North America and Australia | 64 | https://figshare.com/articles/dataset/PGC3_bipolar_disorder_GWAS_summary_statistics/14102594 |

**Table S2. Characteristics of genetic instruments identified by GWAS to be associated with broad depression**

| **SNP** | **Chr** | **Position** | **Effect allele** | **Other allele** | **EAF** | **Beta** | **SE** | **P-value** | **F-statistic** |
| --- | --- | --- | --- | --- | --- | --- | --- | --- | --- |
| rs1002656 | 1 | 37192741 | T | C | 0.703 | -0.021 | 0.002 | 4.84E-19 | 389.128 |
| rs10061069 | 5 | 93071630 | C | G | 0.221 | -0.025 | 0.003 | 5.17E-22 | 455.303 |
| rs10149470 | 14 | 104017953 | A | G | 0.487 | -0.021 | 0.002 | 6.88E-22 | 448.316 |
| rs1021363 | 10 | 106610839 | A | G | 0.355 | 0.023 | 0.002 | 3.77E-23 | 494.375 |
| rs1045430 | 14 | 75130235 | T | G | 0.479 | -0.023 | 0.002 | 4.44E-25 | 539.052 |
| rs10789214 | 1 | 67146817 | T | C | 0.566 | 0.014 | 0.002 | 3.81E-10 | 194.930 |
| rs10817969 | 9 | 119731045 | T | G | 0.717 | 0.017 | 0.002 | 1.32E-12 | 244.887 |
| rs10890020* | 1 | 73668836 | A | G | 0.516 | -0.027 | 0.002 | 3.31E-34 | 753.026 |
| rs10913112 | 1 | 175913828 | T | C | 0.377 | -0.021 | 0.002 | 1.72E-20 | 454.704 |
| rs1095626 | 3 | 157977962 | T | C | 0.580 | -0.029 | 0.002 | 6.31E-39 | 836.918 |
| rs11135349 | 5 | 164523472 | A | C | 0.471 | -0.025 | 0.002 | 2.18E-30 | 658.624 |
| rs113188507 | 1 | 80809636 | A | G | 0.284 | 0.023 | 0.003 | 2.94E-21 | 470.642 |
| rs1152578 | 14 | 64697037 | T | C | 0.436 | -0.015 | 0.002 | 4.19E-12 | 240.187 |
| rs11579246 | 1 | 50559162 | A | G | 0.907 | 0.032 | 0.004 | 8.07E-18 | 366.299 |
| rs12052908* | 2 | 22503044 | A | T | 0.533 | -0.021 | 0.002 | 3.98E-22 | 468.682 |
| rs1226412 | 2 | 157111313 | T | C | 0.792 | 0.023 | 0.003 | 3.35E-18 | 375.334 |
| rs12624433 | 20 | 44680853 | A | G | 0.258 | 0.019 | 0.003 | 2.16E-14 | 295.600 |
| rs12923444 | 16 | 21639710 | A | C | 0.563 | -0.024 | 0.002 | 1.76E-24 | 584.565 |
| rs12966052 | 18 | 52751639 | C | G | 0.181 | -0.019 | 0.003 | 3.76E-11 | 218.711 |
| rs12967143 | 18 | 53099012 | C | G | 0.698 | -0.026 | 0.002 | 1.61E-27 | 583.782 |
| rs12967855 | 18 | 35138245 | A | G | 0.330 | 0.021 | 0.002 | 1.32E-19 | 400.306 |
| rs13084037 | 3 | 49214066 | A | G | 0.774 | -0.022 | 0.003 | 1.79E-17 | 348.302 |
| rs1343605 | 13 | 53647048 | A | C | 0.384 | 0.023 | 0.002 | 1.62E-25 | 533.778 |
| rs1354115 | 9 | 2983774 | A | C | 0.624 | 0.019 | 0.002 | 4.39E-17 | 343.120 |
| rs1409379 | 13 | 31907741 | T | C | 0.764 | 0.016 | 0.003 | 8.26E-11 | 204.986 |
| rs141954845 | 3 | 61192911 | A | G | 0.388 | 0.018 | 0.002 | 1.18E-15 | 321.715 |
| rs143186028 | 20 | 39997404 | T | G | 0.178 | 0.024 | 0.003 | 7.23E-18 | 368.027 |
| rs1448938 | 11 | 30892824 | A | G | 0.417 | 0.017 | 0.002 | 3.74E-15 | 304.137 |
| rs1466887 | 1 | 37709328 | T | C | 0.551 | -0.013 | 0.002 | 1.37E-08 | 163.437 |
| rs1568452 | 2 | 58012833 | T | C | 0.385 | 0.029 | 0.002 | 3.08E-40 | 859.816 |
| rs16887442 | 7 | 82936909 | T | C | 0.435 | 0.015 | 0.002 | 1.25E-11 | 227.591 |
| rs169235 | 1 | 181740924 | A | G | 0.753 | -0.017 | 0.003 | 1.61E-11 | 227.275 |
| rs17641524 | 1 | 197704717 | T | C | 0.209 | -0.024 | 0.003 | 7.90E-20 | 412.941 |
| rs1890946 | 1 | 52342427 | T | C | 0.467 | -0.019 | 0.002 | 8.62E-19 | 396.147 |
| rs1933802 | 6 | 105365891 | C | G | 0.454 | -0.020 | 0.002 | 1.36E-19 | 410.879 |
| rs1956373 | 14 | 60141822 | T | G | 0.744 | -0.017 | 0.003 | 6.08E-12 | 230.247 |
| rs1982277 | 9 | 11513019 | T | C | 0.759 | 0.021 | 0.003 | 1.28E-15 | 327.856 |
| rs198457 | 11 | 61471678 | T | C | 0.193 | -0.021 | 0.003 | 5.41E-14 | 298.201 |
| rs200949 | 6 | 27835435 | A | G | 0.874 | 0.043 | 0.003 | 2.13E-37 | 870.903 |
| rs2029865 | 6 | 165121844 | A | T | 0.453 | -0.018 | 0.002 | 4.02E-17 | 354.793 |
| rs2043539 | 7 | 12253880 | A | G | 0.418 | 0.022 | 0.002 | 8.32E-24 | 497.822 |
| rs2247523 | 7 | 82454404 | C | G | 0.532 | -0.016 | 0.002 | 9.05E-14 | 266.154 |
| rs2509805 | 11 | 57650796 | T | C | 0.321 | 0.019 | 0.002 | 4.92E-16 | 336.167 |
| rs2568958 | 1 | 72765116 | A | G | 0.616 | 0.034 | 0.002 | 4.45E-52 | 1130.077 |
| rs263645 | 9 | 17016503 | A | T | 0.544 | 0.018 | 0.002 | 6.92E-16 | 324.939 |
| rs2670139 | 9 | 126634255 | T | C | 0.761 | -0.018 | 0.003 | 2.19E-12 | 241.001 |
| rs2876520 | 6 | 142996618 | C | G | 0.527 | -0.019 | 0.002 | 2.65E-17 | 368.584 |
| rs301799 | 1 | 8489302 | T | C | 0.569 | -0.025 | 0.002 | 4.05E-31 | 668.978 |
| rs30266 | 5 | 103972357 | A | G | 0.330 | 0.032 | 0.002 | 1.99E-45 | 975.084 |
| rs3099439 | 5 | 87545318 | T | C | 0.529 | -0.021 | 0.002 | 1.61E-21 | 455.861 |
| rs3213572 | 12 | 121205078 | A | G | 0.475 | 0.020 | 0.002 | 5.51E-20 | 405.060 |
| rs33431 | 19 | 30939989 | T | C | 0.614 | 0.012 | 0.002 | 4.69E-08 | 146.658 |
| rs34488670 | 15 | 47684936 | T | C | 0.789 | -0.019 | 0.003 | 4.69E-13 | 262.479 |
| rs34653192 | 9 | 31124452 | C | G | 0.320 | -0.020 | 0.002 | 3.39E-16 | 353.241 |
| rs34937911 | 4 | 42110353 | T | C | 0.884 | 0.024 | 0.003 | 5.39E-13 | 258.528 |
| rs35553410* | 4 | 131237381 | T | C | 0.746 | -0.016 | 0.003 | 2.18E-10 | 202.441 |
| rs3793577 | 9 | 23737627 | A | G | 0.467 | -0.021 | 0.002 | 5.58E-22 | 468.557 |
| rs3823624 | 7 | 2110346 | T | C | 0.807 | 0.029 | 0.003 | 1.61E-26 | 569.995 |
| rs4346585 | 3 | 44736493 | T | C | 0.696 | -0.016 | 0.002 | 4.87E-12 | 237.696 |
| rs45510091 | 4 | 123186393 | A | G | 0.947 | 0.046 | 0.005 | 7.74E-21 | 453.363 |
| rs4772087 | 13 | 99115041 | T | C | 0.373 | 0.021 | 0.002 | 2.34E-20 | 432.083 |
| rs56314503 | 12 | 84465022 | T | G | 0.749 | -0.020 | 0.003 | 1.28E-16 | 327.850 |
| rs56887639 | 16 | 13755530 | A | G | 0.726 | -0.017 | 0.002 | 6.04E-13 | 245.725 |
| rs57344483 | 11 | 127022560 | A | G | 0.926 | -0.024 | 0.004 | 5.26E-09 | 168.487 |
| rs58104186 | 7 | 109099919 | A | G | 0.469 | 0.019 | 0.002 | 3.56E-18 | 380.152 |
| rs58621819* | 11 | 65314830 | A | T | 0.790 | -0.017 | 0.003 | 2.17E-10 | 200.134 |
| rs5995992 | 22 | 41487218 | T | C | 0.716 | -0.030 | 0.002 | 2.60E-35 | 780.011 |
| rs60157091 | 5 | 61509655 | T | C | 0.515 | 0.021 | 0.002 | 5.54E-21 | 443.878 |
| rs61902811 | 11 | 113370758 | A | G | 0.368 | -0.029 | 0.002 | 5.17E-39 | 821.757 |
| rs61990288 | 14 | 42074726 | A | G | 0.508 | -0.025 | 0.002 | 1.96E-31 | 655.344 |
| rs62091461 | 18 | 52488672 | T | C | 0.227 | -0.020 | 0.003 | 1.75E-14 | 294.190 |
| rs62188629* | 2 | 208044470 | A | G | 0.314 | 0.017 | 0.002 | 6.15E-13 | 259.953 |
| rs67436663* | 8 | 71347626 | C | G | 0.240 | -0.014 | 0.003 | 2.83E-08 | 153.411 |
| rs6783233 | 3 | 117509984 | T | C | 0.283 | 0.018 | 0.002 | 1.14E-13 | 268.968 |
| rs7030813 | 9 | 36999369 | T | C | 0.374 | 0.025 | 0.002 | 8.88E-30 | 623.519 |
| rs7117514 | 11 | 70544937 | A | G | 0.542 | -0.017 | 0.002 | 3.64E-14 | 289.268 |
| rs7193263 | 16 | 6315880 | A | G | 0.668 | -0.021 | 0.002 | 3.83E-19 | 398.026 |
| rs7198928 | 16 | 7666402 | T | C | 0.616 | 0.022 | 0.002 | 3.71E-23 | 479.796 |
| rs7200826 | 16 | 13066833 | T | C | 0.255 | 0.027 | 0.003 | 3.09E-28 | 585.830 |
| rs7227069 | 18 | 50731802 | A | G | 0.433 | 0.024 | 0.002 | 2.13E-28 | 607.921 |
| rs7241572 | 18 | 77580712 | A | G | 0.201 | 0.020 | 0.003 | 2.56E-13 | 271.628 |
| rs725616 | 6 | 147950422 | T | C | 0.364 | 0.014 | 0.002 | 1.06E-09 | 178.477 |
| rs72710803 | 1 | 177428018 | A | C | 0.912 | -0.025 | 0.004 | 1.35E-10 | 206.816 |
| rs75581564 | 17 | 27363750 | A | G | 0.117 | 0.025 | 0.003 | 3.49E-13 | 267.674 |
| rs7585722 | 2 | 86819128 | T | C | 0.846 | -0.022 | 0.003 | 5.47E-13 | 254.916 |
| rs7685686 | 4 | 3207142 | A | G | 0.575 | 0.017 | 0.002 | 5.52E-15 | 309.206 |
| rs7758630 | 6 | 101387304 | A | T | 0.405 | -0.017 | 0.002 | 7.79E-14 | 277.426 |
| rs7807677 | 7 | 117502574 | T | C | 0.551 | 0.021 | 0.002 | 5.40E-22 | 465.868 |
| rs78337797 | 12 | 23987925 | T | G | 0.878 | 0.023 | 0.003 | 1.37E-11 | 237.347 |
| rs7837935 | 8 | 65562019 | T | G | 0.152 | -0.023 | 0.003 | 1.15E-13 | 278.675 |
| rs7932640 | 11 | 88744425 | T | C | 0.442 | 0.023 | 0.002 | 3.40E-25 | 537.369 |
| rs8037355 | 15 | 37643831 | T | C | 0.556 | -0.019 | 0.002 | 1.52E-18 | 384.887 |
| rs913930 | 9 | 120484009 | A | G | 0.643 | -0.022 | 0.002 | 1.03E-21 | 465.389 |
| rs9363467* | 6 | 66565703 | T | C | 0.604 | 0.016 | 0.002 | 2.50E-12 | 252.582 |
| rs9545360* | 13 | 80826373 | A | C | 0.181 | -0.017 | 0.003 | 6.70E-09 | 174.576 |
| rs9592461 | 13 | 66941792 | A | G | 0.487 | 0.024 | 0.002 | 1.17E-28 | 623.908 |
| rs997934 | 10 | 1795194 | T | C | 0.380 | 0.016 | 0.002 | 4.60E-13 | 264.544 |

*IV not available in preterm birth GWAS. EAF: Effect allele frequency, Standard error: SE. F-statistic was calculated using the following formulas: F=R^2 (n-2)⁄(1-R^2) and R^2=2×β^2×MAF×(1-MAF), where F represents F-statistic, R^2 represents the phenotypic variance explained by a genetic instrument, n is the sample size, β is the estimated genetic association of SNP with the exposure, MAF is the minor allele frequency. SNP, single nucleotide polymorphisms.

**Table S3. Characteristics of genetic instruments identified by GWAS to be associated with major depression disorder**

| **SNP** | **Chr** | **Position** | **Effect allele** | **Other allele** | **EAF** | **Beta** | **SE** | ***P*-value** | ***F*-statistic** |
| --- | --- | --- | --- | --- | --- | --- | --- | --- | --- |
| rs159963 | 1 | 8504421 | A | C | 0.56 | -0.030 | 0.005 | 3.20E-08 | 219.721 |
| rs1432639 | 1 | 72813218 | A | C | 0.63 | 0.039 | 0.005 | 4.60E-15 | 344.730 |
| rs12129573 | 1 | 73768366 | A | C | 0.37 | 0.039 | 0.005 | 4.00E-12 | 344.730 |
| rs2389016 | 1 | 80799329 | T | C | 0.28 | 0.030 | 0.005 | 1.00E-08 | 169.282 |
| rs4261101 | 1 | 90796053 | A | G | 0.37 | -0.030 | 0.005 | 1.00E-08 | 207.855 |
| rs9427672 | 1 | 197754741 | A | G | 0.24 | -0.030 | 0.006 | 3.10E-08 | 162.631 |
| rs11682175 | 2 | 57987593 | T | C | 0.52 | -0.030 | 0.005 | 4.70E-09 | 222.575 |
| rs1226412 | 2 | 157111313 | T | C | 0.79 | 0.030 | 0.006 | 2.40E-08 | 139.296 |
| rs7430565 | 3 | 158107180 | A | G | 0.58 | -0.030 | 0.005 | 2.90E-09 | 217.223 |
| rs34215985 | 4 | 42047778 | C | G | 0.24 | -0.041 | 0.006 | 3.10E-09 | 292.194 |
| rs116755193* | 5 | 124251883 | T | C | 0.38 | -0.030 | 0.005 | 7.00E-09 | 210.086 |
| rs11135349 | 5 | 164523472 | A | C | 0.48 | -0.030 | 0.005 | 1.10E-09 | 222.575 |
| rs4869056 | 5 | 166992078 | A | G | 0.63 | -0.030 | 0.005 | 6.80E-09 | 207.855 |
| rs115507122* | 6 | 30737591 | C | G | 0.18 | -0.041 | 0.006 | 3.30E-11 | 236.419 |
| rs9402472 | 6 | 99566521 | A | G | 0.24 | 0.030 | 0.006 | 2.80E-08 | 153.155 |
| rs10950398 | 7 | 12264871 | A | G | 0.41 | 0.030 | 0.005 | 2.60E-08 | 203.136 |
| rs12666117 | 7 | 109105611 | A | G | 0.47 | 0.030 | 0.005 | 1.40E-08 | 209.185 |
| rs1354115 | 9 | 2983774 | A | C | 0.62 | 0.030 | 0.005 | 2.40E-08 | 197.844 |
| rs10959913 | 9 | 11544964 | T | G | 0.76 | 0.030 | 0.006 | 5.10E-09 | 153.155 |
| rs7856424 | 9 | 119733595 | T | C | 0.29 | -0.030 | 0.005 | 8.50E-09 | 183.592 |
| rs7029033* | 9 | 126682068 | T | C | 0.07 | 0.049 | 0.009 | 2.70E-08 | 148.927 |
| rs61867293 | 10 | 106563924 | T | C | 0.2 | -0.041 | 0.006 | 7.00E-10 | 256.291 |
| rs1806153 | 11 | 31850105 | T | G | 0.22 | 0.039 | 0.006 | 1.20E-09 | 253.730 |
| rs4074723 | 12 | 23947737 | A | C | 0.41 | -0.030 | 0.005 | 3.10E-08 | 215.706 |
| rs4143229 | 13 | 44327799 | A | C | 0.92 | -0.051 | 0.009 | 2.50E-08 | 186.106 |
| rs12552 | 13 | 53625781 | A | G | 0.44 | 0.039 | 0.005 | 6.10E-19 | 364.414 |
| rs4904738 | 14 | 42179732 | T | C | 0.57 | -0.030 | 0.005 | 2.60E-09 | 218.561 |
| rs915057 | 14 | 64686207 | A | G | 0.42 | -0.030 | 0.005 | 7.60E-10 | 217.223 |
| rs10149470 | 14 | 104017953 | A | G | 0.49 | -0.030 | 0.005 | 3.10E-09 | 222.843 |
| rs8025231 | 15 | 37648402 | A | C | 0.57 | -0.030 | 0.005 | 2.40E-12 | 218.561 |
| rs8063603 | 16 | 6310645 | A | G | 0.65 | -0.030 | 0.005 | 6.90E-09 | 202.860 |
| rs7198928 | 16 | 7666402 | T | C | 0.62 | 0.030 | 0.005 | 1.00E-08 | 197.844 |
| rs7200826 | 16 | 13066833 | T | C | 0.25 | 0.030 | 0.006 | 2.40E-08 | 157.439 |
| rs11643192 | 16 | 72214276 | A | C | 0.41 | 0.030 | 0.005 | 3.40E-08 | 203.136 |
| rs17727765 | 17 | 27576962 | T | C | 0.92 | -0.051 | 0.009 | 8.50E-09 | 186.106 |
| rs62099069 | 18 | 36883737 | A | T | 0.42 | -0.030 | 0.005 | 1.30E-08 | 217.223 |
| rs11663393 | 18 | 50614732 | A | G | 0.45 | 0.030 | 0.005 | 1.60E-08 | 207.841 |
| rs1833288 | 18 | 52517906 | A | G | 0.72 | 0.030 | 0.005 | 2.60E-08 | 169.282 |
| rs12958048 | 18 | 53101598 | A | G | 0.33 | 0.030 | 0.005 | 3.60E-11 | 185.663 |
| rs5758265* | 22 | 41617897 | A | G | 0.28 | 0.030 | 0.005 | 7.60E-09 | 169.282 |

*IV not available in preterm birth GWAS. EAF: Effect allele frequency, Standard error: SE. F-statistic was calculated using the following formulas: F=R^2 (n-2)⁄(1-R^2) and R^2=2×β^2×MAF×(1-MAF), where F represents F-statistic, R^2 represents the phenotypic variance explained by a genetic instrument, n is the sample size, β is the estimated genetic association of SNP with the exposure, MAF is the minor allele frequency. SNP, single nucleotide polymorphisms.

**Table S4. Characteristics of genetic instruments identified by GWAS to be associated with bipolar disorder**

| **SNP** | **Chr** | **Position** | **Effect allele** | **Other allele** | **EAF** | **Beta** | **SE** | ***P*-value** | ***F*-statistic** |
| --- | --- | --- | --- | --- | --- | --- | --- | --- | --- |
| rs10043984 | 5 | 137712121 | T | C | 0.236 | 0.060 | 0.011 | 3.70E-08 | 541.523 |
| rs10255167 | 7 | 140676153 | A | G | 0.778 | 0.066 | 0.012 | 1.60E-08 | 620.568 |
| rs10455979 | 6 | 166995260 | G | C | 0.5 | 0.055 | 0.01 | 4.20E-09 | 637.805 |
| rs10737496 | 1 | 163745389 | C | T | 0.444 | 0.054 | 0.009 | 7.20E-09 | 608.438 |
| rs10761661 | 10 | 64525135 | T | C | 0.472 | 0.052 | 0.009 | 4.70E-08 | 551.693 |
| rs10866641 | 5 | 169289206 | T | C | 0.575 | 0.063 | 0.009 | 2.80E-11 | 804.915 |
| rs10973201 | 9 | 37090538 | C | T | 0.11 | 0.096 | 0.017 | 2.50E-08 | 752.676 |
| rs10994415 | 10 | 62322034 | C | T | 0.082 | 0.118 | 0.017 | 1.10E-11 | 867.455 |
| rs11062170 | 12 | 2348844 | C | G | 0.333 | 0.078 | 0.01 | 1.90E-15 | 1119.911 |
| rs112481526 | 4 | 123076007 | G | A | 0.256 | 0.063 | 0.011 | 1.90E-09 | 627.076 |
| rs113779084 | 7 | 11871787 | A | G | 0.299 | 0.076 | 0.01 | 1.40E-13 | 1006.893 |
| rs115694474 | 3 | 70488788 | T | A | 0.799 | 0.066 | 0.012 | 2.40E-08 | 576.971 |
| rs11764361 | 7 | 105043229 | A | G | 0.668 | 0.061 | 0.01 | 3.50E-09 | 687.327 |
| rs11870683 | 17 | 38129841 | T | A | 0.65 | 0.057 | 0.01 | 2.80E-08 | 620.636 |
| rs12289486 | 11 | 79092527 | T | C | 0.115 | 0.083 | 0.015 | 3.30E-08 | 575.017 |
| rs12575685 | 11 | 70517927 | A | G | 0.327 | 0.065 | 0.01 | 1.20E-10 | 768.630 |
| rs12668848 | 7 | 2020995 | G | A | 0.575 | 0.057 | 0.01 | 1.90E-09 | 666.747 |
| rs12672003 | 7 | 24647222 | G | A | 0.113 | 0.092 | 0.016 | 2.70E-09 | 699.325 |
| rs12932628 | 16 | 89632725 | T | G | 0.487 | 0.056 | 0.01 | 6.70E-09 | 659.339 |
| rs13044225 | 20 | 60865815 | G | A | 0.44 | 0.054 | 0.01 | 8.50E-09 | 607.293 |
| rs13195402 | 6 | 26463575 | G | T | 0.919 | 0.136 | 0.018 | 5.80E-15 | 1149.129 |
| rs13417268 | 2 | 169481837 | C | G | 0.758 | 0.062 | 0.011 | 2.10E-08 | 585.995 |
| rs1487445 | 6 | 98565211 | T | C | 0.487 | 0.075 | 0.009 | 1.50E-15 | 1171.535 |
| rs17183814 | 2 | 166152389 | G | A | 0.924 | 0.103 | 0.019 | 2.70E-08 | 613.157 |
| rs174592 | 11 | 61618608 | G | A | 0.395 | 0.071 | 0.01 | 9.90E-14 | 1012.053 |
| rs1998820 | 10 | 18751103 | T | A | 0.886 | 0.083 | 0.015 | 4.10E-08 | 583.477 |
| rs2011302 | 2 | 193738336 | A | T | 0.377 | 0.054 | 0.01 | 4.30E-08 | 558.857 |
| rs2126180 | 1 | 61105668 | A | G | 0.457 | 0.056 | 0.009 | 1.60E-09 | 654.898 |
| rs2273738 | 10 | 111648659 | T | C | 0.135 | 0.092 | 0.014 | 1.60E-11 | 814.981 |
| rs228768 | 17 | 42191893 | G | T | 0.294 | 0.065 | 0.01 | 2.80E-10 | 724.871 |
| rs2336147 | 3 | 52626443 | T | C | 0.498 | 0.068 | 0.009 | 3.60E-13 | 950.805 |
| rs237460 | 20 | 48033127 | T | C | 0.412 | 0.055 | 0.009 | 4.30E-09 | 618.019 |
| rs2693698 | 14 | 99719219 | G | A | 0.551 | 0.054 | 0.009 | 2.00E-08 | 588.709 |
| rs2719164 | 2 | 194437889 | A | G | 0.564 | 0.052 | 0.01 | 4.90E-08 | 544.351 |
| rs28455634 | 16 | 9230816 | G | A | 0.62 | 0.063 | 0.01 | 2.60E-10 | 775.958 |
| rs28565152 | 5 | 7542911 | A | G | 0.238 | 0.068 | 0.011 | 2.00E-09 | 689.314 |
| rs2953928 | 8 | 34152492 | A | G | 0.067 | 0.117 | 0.02 | 6.30E-09 | 709.248 |
| rs3088186 | 8 | 10226355 | T | C | 0.287 | 0.056 | 0.01 | 2.10E-08 | 539.894 |
| rs35306827 | 13 | 113869045 | G | A | 0.775 | 0.066 | 0.011 | 3.60E-09 | 626.537 |
| rs35958438 | 15 | 38973793 | G | A | 0.772 | 0.064 | 0.012 | 3.80E-08 | 596.866 |
| rs4331993 | 6 | 152793572 | A | T | 0.382 | 0.054 | 0.01 | 2.00E-08 | 581.812 |
| rs4447398 | 15 | 42904904 | A | C | 0.131 | 0.083 | 0.014 | 2.60E-09 | 643.283 |
| rs4619651 | 2 | 97416153 | G | A | 0.67 | 0.066 | 0.01 | 4.80E-11 | 794.744 |
| rs4672 | 11 | 64009879 | A | G | 0.083 | 0.102 | 0.017 | 3.40E-09 | 652.971 |
| rs4702 | 15 | 91426560 | G | A | 0.446 | 0.057 | 0.01 | 3.50E-09 | 674.150 |
| rs475805 | 11 | 65848738 | A | G | 0.767 | 0.068 | 0.011 | 2.00E-09 | 679.244 |
| rs4790841 | 17 | 1835482 | T | C | 0.151 | 0.072 | 0.013 | 3.10E-08 | 556.556 |
| rs5758064 | 22 | 41153879 | T | C | 0.523 | 0.053 | 0.009 | 2.00E-08 | 572.774 |
| rs6032110 | 20 | 43944323 | A | G | 0.512 | 0.057 | 0.009 | 1.00E-09 | 681.725 |
| rs61554907 | 17 | 38220432 | T | G | 0.124 | 0.087 | 0.015 | 1.60E-08 | 684.136 |
| rs62011709 | 15 | 83531774 | T | A | 0.747 | 0.062 | 0.011 | 1.40E-08 | 603.767 |
| rs62489493 | 8 | 9763581 | G | C | 0.128 | 0.090 | 0.014 | 2.60E-11 | 748.123 |
| rs62581014 | 9 | 141066490 | T | C | 0.366 | 0.065 | 0.012 | 2.80E-08 | 810.529 |
| rs67712855 | 20 | 43682551 | T | G | 0.687 | 0.068 | 0.01 | 4.20E-11 | 817.561 |
| rs678397 | 11 | 66324583 | T | C | 0.457 | 0.054 | 0.009 | 5.50E-09 | 611.615 |
| rs6865469 | 5 | 78849505 | T | G | 0.274 | 0.058 | 0.01 | 1.70E-08 | 560.615 |
| rs6887473 | 5 | 80961069 | G | A | 0.739 | 0.060 | 0.011 | 8.80E-09 | 579.344 |
| rs6946056 | 7 | 131870597 | C | A | 0.623 | 0.054 | 0.01 | 3.70E-08 | 558.857 |
| rs6954854 | 7 | 21492589 | G | A | 0.425 | 0.058 | 0.009 | 5.90E-10 | 688.920 |
| rs696366 | 3 | 107757060 | C | A | 0.55 | 0.052 | 0.009 | 4.50E-08 | 547.889 |
| rs6992333 | 8 | 144993377 | G | A | 0.41 | 0.060 | 0.01 | 1.60E-09 | 726.845 |
| rs7199910 | 16 | 9926348 | G | T | 0.312 | 0.055 | 0.01 | 1.70E-08 | 547.516 |
| rs748455 | 15 | 85149575 | T | C | 0.719 | 0.068 | 0.01 | 5.00E-11 | 768.073 |
| rs9834970 | 3 | 36856030 | C | T | 0.481 | 0.083 | 0.009 | 6.60E-19 | 1445.100 |

*IV not available in preterm birth GWAS. EAF: Effect allele frequency, Standard error: SE. F-statistic was calculated using the following formulas: F=R^2 (n-2)⁄(1-R^2) and R^2=2×β^2×MAF×(1-MAF), where F represents F-statistic, R^2 represents the phenotypic variance explained by a genetic instrument, n is the sample size, β is the estimated genetic association of SNP with the exposure, MAF is the minor allele frequency. SNP, single nucleotide polymorphisms.

**Table S5. Characteristics of independent genetic instruments identified by Finngen to be associated with preterm birth (P<5E-05)**

| **SNP** | **Chr** | **Position** | **Effect allele** | **Other allele** | **EAF** | **Beta** | **SE** | ***P*-value** | ***F*-statistic** |
| --- | --- | --- | --- | --- | --- | --- | --- | --- | --- |
| rs4440857 | 1 | 11626988 | A | G | 0.808 | -0.087 | 0.020 | 1.06E-05 | 400.548 |
| rs80173514 | 1 | 22028045 | A | C | 0.190 | -0.086 | 0.020 | 2.81E-05 | 388.795 |
| rs2492077 | 1 | 41494393 | A | C | 0.756 | -0.089 | 0.018 | 1.03E-06 | 498.185 |
| rs111695916 | 1 | 95592564 | T | C | 0.038 | -0.181 | 0.044 | 3.86E-05 | 412.834 |
| rs10798038 | 1 | 186217547 | A | T | 0.610 | -0.068 | 0.016 | 2.54E-05 | 380.425 |
| rs3120966 | 1 | 187703116 | G | A | 0.802 | 0.082 | 0.020 | 4.30E-05 | 369.080 |
| rs3790798 | 1 | 200148179 | G | C | 0.006 | -0.513 | 0.124 | 3.57E-05 | 503.607 |
| rs1380255 | 1 | 221483024 | T | C | 0.375 | 0.066 | 0.016 | 4.50E-05 | 351.254 |
| rs112379369 | 1 | 224452521 | T | C | 0.272 | -0.073 | 0.018 | 4.85E-05 | 362.288 |
| rs142340597 | 1 | 227398128 | A | G | 0.032 | -0.204 | 0.047 | 1.57E-05 | 450.112 |
| rs4669669 | 2 | 11010391 | A | C | 0.766 | -0.083 | 0.018 | 7.87E-06 | 420.412 |
| rs11690571 | 2 | 28852165 | T | C | 0.489 | -0.070 | 0.016 | 8.52E-06 | 422.147 |
| rs74521447 | 2 | 69118339 | A | G | 0.042 | 0.156 | 0.038 | 4.51E-05 | 337.865 |
| rs10779902 | 2 | 99310151 | G | C | 0.359 | -0.069 | 0.017 | 3.66E-05 | 371.954 |
| rs72849548 | 2 | 141022037 | T | C | 0.149 | 0.098 | 0.022 | 5.06E-06 | 420.806 |
| rs1860165 | 2 | 158046477 | C | T | 0.689 | -0.072 | 0.017 | 1.88E-05 | 384.223 |
| rs114864719 | 2 | 168657619 | C | T | 0.215 | 0.078 | 0.019 | 3.88E-05 | 353.567 |
| rs7591150 | 2 | 172032858 | C | G | 0.619 | -0.077 | 0.016 | 1.56E-06 | 486.134 |
| rs3769996 | 2 | 177472668 | A | G | 0.105 | 0.116 | 0.025 | 4.34E-06 | 431.654 |
| rs141592655 | 2 | 192471322 | T | C | 0.033 | -0.216 | 0.047 | 3.59E-06 | 513.393 |
| rs80346967 | 2 | 205545631 | G | A | 0.008 | 0.346 | 0.080 | 1.49E-05 | 339.166 |
| rs12692246 | 2 | 238804807 | G | C | 0.728 | -0.072 | 0.018 | 4.91E-05 | 347.680 |
| rs17049160 | 3 | 8459596 | T | C | 0.136 | -0.097 | 0.023 | 3.60E-05 | 377.755 |
| rs322694 | 3 | 25307943 | A | C | 0.827 | 0.095 | 0.021 | 8.42E-06 | 446.606 |
| rs6768627 | 3 | 46853886 | T | C | 0.075 | -0.132 | 0.031 | 2.14E-05 | 413.879 |
| rs73125696 | 3 | 64908328 | C | G | 0.109 | 0.109 | 0.025 | 1.17E-05 | 393.633 |
| rs73180196 | 3 | 105162915 | C | G | 0.269 | -0.077 | 0.018 | 1.81E-05 | 403.682 |
| rs11710590 | 3 | 127182271 | C | T | 0.700 | -0.072 | 0.017 | 2.63E-05 | 369.336 |
| rs6441042 | 3 | 156173300 | G | C | 0.519 | -0.078 | 0.016 | 6.81E-07 | 526.226 |
| rs548434848 | 3 | 175134436 | C | G | 0.014 | -0.305 | 0.075 | 4.17E-05 | 450.874 |
| rs148298502 | 4 | 27491638 | C | T | 0.012 | 0.328 | 0.066 | 7.45E-07 | 444.885 |
| rs115227367 | 4 | 52785413 | T | C | 0.000 | 2.044 | 0.474 | 1.63E-05 | 157.720 |
| rs41421249 | 4 | 80135867 | G | A | 0.164 | -0.094 | 0.022 | 1.29E-05 | 420.538 |
| rs6844159 | 4 | 120873380 | C | T | 0.328 | -0.069 | 0.017 | 4.66E-05 | 359.594 |
| rs9968523 | 4 | 145703857 | A | G | 0.444 | -0.068 | 0.016 | 2.20E-05 | 387.111 |
| rs13121102 | 4 | 180901891 | A | T | 0.012 | 0.271 | 0.065 | 3.39E-05 | 303.114 |
| rs80004232 | 5 | 3137637 | A | G | 0.095 | -0.112 | 0.028 | 4.94E-05 | 372.429 |
| rs27099 | 5 | 14463530 | T | A | 0.159 | -0.091 | 0.022 | 3.90E-05 | 377.639 |
| rs2946160 | 5 | 158464870 | A | G | 0.785 | -0.099 | 0.019 | 2.14E-07 | 563.822 |
| rs12201668 | 6 | 6171302 | G | T | 0.120 | 0.100 | 0.024 | 3.25E-05 | 361.585 |
| rs28732155 | 6 | 31644798 | A | C | 0.003 | 0.906 | 0.187 | 1.20E-06 | 981.125 |
| rs9275373 | 6 | 32700634 | A | G | 0.120 | 0.103 | 0.024 | 1.51E-05 | 382.410 |
| rs538146388 | 6 | 44586148 | G | C | 0.145 | 0.092 | 0.022 | 2.83E-05 | 357.316 |
| rs112762066 | 6 | 56973363 | G | C | 0.000 | -57.33 | 13.192 | 1.38E-05 | 109442.06 |
| rs62424178 | 6 | 102682629 | T | G | 0.027 | -0.234 | 0.052 | 6.25E-06 | 498.772 |
| rs6913081 | 6 | 117725796 | A | G | 0.058 | 0.135 | 0.033 | 4.60E-05 | 339.875 |
| rs654391 | 6 | 168674977 | C | T | 0.339 | 0.069 | 0.017 | 3.35E-05 | 363.998 |
| rs141807562 | 7 | 10629245 | A | G | 0.004 | -0.671 | 0.150 | 7.23E-06 | 645.580 |
| rs191606400 | 7 | 12598123 | G | A | 0.006 | 0.398 | 0.096 | 3.53E-05 | 310.303 |
| rs143851711 | 7 | 66732300 | T | G | 0.011 | 0.285 | 0.070 | 4.85E-05 | 306.743 |
| rs6960852 | 7 | 68164650 | T | C | 0.368 | -0.076 | 0.017 | 5.85E-06 | 457.104 |
| rs77654770 | 7 | 74480243 | T | C | 0.006 | 0.403 | 0.092 | 1.29E-05 | 338.529 |
| rs41487549 | 7 | 84209561 | G | A | 0.050 | 0.144 | 0.035 | 4.02E-05 | 341.487 |
| rs143529707 | 7 | 104587347 | T | C | 0.027 | 0.192 | 0.046 | 3.33E-05 | 326.189 |
| rs138127577 | 7 | 133402625 | T | A | 0.008 | 0.327 | 0.079 | 3.86E-05 | 298.852 |
| rs10487624 | 7 | 144177097 | T | C | 0.321 | -0.072 | 0.017 | 3.06E-05 | 382.303 |
| rs28419237 | 7 | 153822065 | C | G | 0.155 | 0.099 | 0.021 | 3.90E-06 | 437.374 |
| rs533629 | 7 | 154742544 | C | T | 0.232 | 0.083 | 0.018 | 7.90E-06 | 416.942 |
| rs139295429 | 8 | 3686035 | G | A | 0.006 | 0.384 | 0.092 | 2.98E-05 | 304.649 |
| rs117329781 | 8 | 3832735 | A | G | 0.039 | -0.185 | 0.043 | 1.85E-05 | 434.929 |
| rs12547019 | 8 | 46943262 | A | C | 0.028 | -0.213 | 0.051 | 2.86E-05 | 431.838 |
| rs12682503 | 8 | 61733254 | T | C | 0.228 | 0.078 | 0.019 | 2.88E-05 | 364.643 |
| rs3110427 | 8 | 106583556 | G | A | 0.542 | 0.068 | 0.016 | 1.66E-05 | 399.246 |
| rs7017937 | 8 | 138963980 | A | G | 0.447 | -0.069 | 0.016 | 2.33E-05 | 399.609 |
| rs148586695 | 9 | 147759 | A | G | 0.030 | -0.234 | 0.053 | 1.01E-05 | 543.674 |
| rs148537967 | 9 | 3889844 | T | C | 0.024 | 0.244 | 0.048 | 4.27E-07 | 481.985 |
| rs17350373 | 9 | 34284946 | A | G | 0.410 | -0.066 | 0.016 | 4.29E-05 | 358.642 |
| rs13289548 | 9 | 103109587 | A | G | 0.008 | 0.349 | 0.084 | 2.91E-05 | 316.567 |
| rs1333908 | 9 | 119911021 | G | T | 0.627 | 0.071 | 0.016 | 1.53E-05 | 402.985 |
| rs2767775 | 9 | 124192455 | T | C | 0.147 | -0.093 | 0.023 | 4.21E-05 | 370.913 |
| rs73556671 | 9 | 136155703 | A | G | 0.012 | 0.294 | 0.068 | 1.45E-05 | 355.435 |
| rs7904257 | 10 | 5838525 | T | G | 0.095 | 0.107 | 0.026 | 4.96E-05 | 336.521 |
| rs182368169 | 10 | 11371358 | G | C | 0.008 | -0.459 | 0.101 | 5.99E-06 | 588.311 |
| rs1904610 | 10 | 67263359 | G | T | 0.120 | 0.101 | 0.024 | 2.01E-05 | 368.007 |
| rs10785997 | 10 | 91206064 | T | G | 0.939 | 0.148 | 0.035 | 1.90E-05 | 429.442 |
| rs10787893 | 10 | 119010406 | G | A | 0.413 | 0.069 | 0.016 | 1.77E-05 | 391.904 |
| rs10767939 | 11 | 32492550 | A | G | 0.199 | 0.082 | 0.019 | 2.43E-05 | 369.766 |
| rs12302187 | 12 | 1414978 | G | A | 0.349 | -0.070 | 0.017 | 3.10E-05 | 380.509 |
| rs1479107 | 12 | 13318113 | G | A | 0.765 | -0.087 | 0.018 | 1.81E-06 | 471.028 |
| rs116984590 | 12 | 16177950 | T | C | 0.013 | -0.351 | 0.080 | 1.14E-05 | 525.666 |
| rs78938480 | 12 | 43645102 | A | G | 0.044 | 0.152 | 0.037 | 4.03E-05 | 330.652 |
| rs76574446 | 12 | 46815686 | T | C | 0.080 | 0.125 | 0.028 | 9.50E-06 | 394.612 |
| rs6582297 | 12 | 75714534 | A | G | 0.273 | 0.075 | 0.018 | 2.02E-05 | 383.837 |
| rs12818849 | 12 | 90286994 | A | G | 0.034 | -0.206 | 0.046 | 7.73E-06 | 477.081 |
| rs188729898 | 12 | 121757737 | T | C | 0.017 | 0.247 | 0.060 | 4.19E-05 | 339.878 |
| rs12309362 | 12 | 132466447 | A | G | 0.598 | 0.069 | 0.016 | 2.33E-05 | 395.150 |
| rs9512910 | 13 | 27888624 | C | T | 0.213 | 0.086 | 0.019 | 6.38E-06 | 426.159 |
| rs184771458 | 13 | 68549325 | T | C | 0.022 | -0.263 | 0.060 | 1.02E-05 | 502.046 |
| rs6491928 | 13 | 105096865 | G | A | 0.937 | 0.138 | 0.033 | 3.53E-05 | 385.373 |
| rs2031936 | 14 | 25110466 | A | G | 0.164 | -0.100 | 0.022 | 4.02E-06 | 472.554 |
| rs12879967 | 14 | 42562750 | C | T | 0.295 | -0.072 | 0.017 | 3.65E-05 | 370.167 |
| rs76827115 | 14 | 43889834 | A | G | 0.008 | -0.421 | 0.102 | 3.45E-05 | 483.481 |
| rs74624686 | 14 | 56487791 | G | T | 0.101 | 0.113 | 0.026 | 1.23E-05 | 399.424 |
| rs198225 | 14 | 56756010 | C | T | 0.986 | 0.311 | 0.074 | 2.41E-05 | 453.428 |
| rs7159452 | 14 | 99293860 | C | G | 0.228 | -0.080 | 0.019 | 2.84E-05 | 385.774 |
| rs4906919 | 15 | 24843957 | G | A | 0.361 | -0.078 | 0.017 | 2.75E-06 | 482.344 |
| rs72740258 | 15 | 52745351 | G | A | 0.016 | -0.297 | 0.070 | 1.94E-05 | 482.497 |
| rs12915633 | 15 | 96787860 | C | T | 0.762 | 0.084 | 0.019 | 7.31E-06 | 439.901 |
| rs60476972 | 16 | 9058806 | A | G | 0.504 | 0.069 | 0.016 | 1.24E-05 | 410.089 |
| rs7199627 | 16 | 10567244 | C | T | 0.047 | -0.161 | 0.039 | 4.31E-05 | 396.989 |
| rs4338802 | 16 | 65538967 | C | T | 0.143 | 0.094 | 0.022 | 2.58E-05 | 368.598 |
| rs6500485 | 16 | 88656634 | A | G | 0.131 | -0.104 | 0.024 | 1.40E-05 | 421.925 |
| rs560 | 17 | 78166054 | T | C | 0.035 | -0.192 | 0.046 | 3.46E-05 | 420.759 |
| rs35424237 | 17 | 78975144 | G | A | 0.083 | 0.118 | 0.028 | 2.60E-05 | 363.403 |
| rs78081143 | 18 | 27725956 | A | G | 0.038 | -0.176 | 0.043 | 4.43E-05 | 395.074 |
| rs62095615 | 18 | 32226947 | T | G | 0.121 | 0.101 | 0.024 | 2.14E-05 | 367.870 |
| rs8109033 | 19 | 3812036 | T | C | 0.556 | 0.074 | 0.016 | 4.67E-06 | 469.622 |
| rs12975912 | 19 | 31081156 | G | A | 0.051 | 0.181 | 0.034 | 8.83E-08 | 545.932 |
| rs2287953 | 19 | 38817285 | T | C | 0.259 | -0.074 | 0.018 | 4.29E-05 | 362.913 |
| rs143820692 | 19 | 50823694 | T | C | 0.031 | -0.207 | 0.048 | 1.77E-05 | 442.771 |
| rs150026473 | 19 | 57234219 | A | G | 0.016 | -0.290 | 0.069 | 2.54E-05 | 452.804 |
| rs6026301 | 20 | 58483922 | C | A | 0.504 | 0.068 | 0.016 | 1.70E-05 | 397.338 |
| rs7276718 | 21 | 19034970 | C | T | 0.495 | 0.070 | 0.016 | 1.20E-05 | 418.473 |
| rs112606994 | 22 | 35405942 | T | C | 0.093 | -0.119 | 0.028 | 2.38E-05 | 409.697 |
| rs112979011 | 22 | 35648305 | T | G | 0.009 | 0.315 | 0.077 | 4.25E-05 | 303.734 |

The cut off of the P-value is <5E-05. EAF: Effect allele frequency, Standard error: SE. F-statistic was calculated using the following formulas: F=R^2 (n-2)⁄(1-R^2) and R^2=2×β^2×MAF×(1-MAF), where F represents F-statistic, R^2 represents the phenotypic variance explained by a genetic instrument, n is the sample size, β is the estimated genetic association of SNP with the exposure, MAF is the minor allele frequency. SNP, single nucleotide polymorphisms.

**Table S6. Functional annotation for the shared SNPs identified by CPASSOC through HeploReg V4.2**

| **Index SNP** | **chr** | **pos (hg38)** | **Ref** | **Alt** | **ASN freq** | **EUR freq** | **dbSNP func annot** | **Promoter histone marks**1 | **Enhancer histone marks**2 | **DNAse**3 | **Proteins bound**4 | **Motifs changed**5 | **GENCODE genes** |
| --- | --- | --- | --- | --- | --- | --- | --- | --- | --- | --- | --- | --- | --- |
| ***Broad depression and preterm birth*** | | | | | | | | | | | | | |
| rs13220522 | 6 | 26316067 | G | A | 0 | 0.07 | - | - | LIV | - | - | 8 altered motifs | 31kb 5' of HIST1H4H |
| rs2734837 | 11 | 113416107 | C | T | 0.06 | 0.60 | intronic | - | - | BLD | - | Hand1 | DRD2 |
| ***Major depression and preterm birth*** | | | | | | | | | | | | | |
| rs149543464 | 6 | 30432986 | G | A | 0 | 0.09 | - | - | - | - | - | - | 56kb 5' of HLA-E |
| rs57440165 | 6 | 26875738 | A | C | 0 | 0.07 | intronic | - | - | - | - | 8 altered motifs | 145kb 5' of TR-I2 |
| ***Bipolar disease and preterm birth*** | | | | | | | | | | | | | |
| rs7813444 | 8 | 64524949 | A | G | 0.60 | 0.33 | - | - | - | - | - | 13 altered motifs | 49kb 3' of RP11-21C4.1 |
| rs9273363 | 6 | 32658495 | C | A | 0.35 | 0.27 | - | - | 5 tissues | BLD,BLD | POL24H8 | 4 altered motifs | 971bp 3' of HLA-DQB1 |
| rs3132948 | 6 | 32229442 | T | G | 0.66 | 0.43 | - | - | - | - | - | GATA,ZID | 5.4kb 5' of NOTCH4 |
| rs1264349 | 6 | 30828882 | A | G | 0 | 0.09 | - | 5 tissues | 22 tissues | 24 tissues | MAFK,CTCF | - | BTN2A1 |
| rs60476972 | 16 | 9058806 | G | A | 0.54 | 0.48 | - | - | - | - | - | Pou2f2 | 9.7kb 5' of RP11-473I1.6 |

^1^Evidence of local H3K4Me1 and H3K27Ac modification (cell lines/types: if >3, only the number is included).

^2^Evidence of local H3K4Me3 modification (cell lines/types: if >3, only the number is included).

^3^Evidence of chromatin hypersensitivity to DNase (cell lines/types: if >3, only the number is included).

^4^ChIP-seq experiments indicate alteration in binding of transcription factor (if >3, only the number is included).

^5^Evidence of alteration in regulatory motif (if >3, only the number is included).

**Table S7. Detailed annotation of the shared SNPs identified from cross-trait meta-analysis through VEP.**

| **Index SNP** | **Chromosome: Position** | **Consequence** | **SYMBOL** | **BIOTYPE** | **Feature_type** |
| --- | --- | --- | --- | --- | --- |
| ***Broad depression and preterm birth*** | | | | |  |
| rs2734837 | 11:113416107-113416107 | intron_variant | DRD2 | protein_coding | Transcript |
| rs2734837 | 11:113416107-113416107 | intron_variant | DRD2 | protein_coding | Transcript |
| rs2734837 | 11:113416107-113416107 | intron_variant,non_coding_transcript_variant | DRD2 | protein_coding_CDS_not_defined | Transcript |
| rs2734837 | 11:113416107-113416107 | intron_variant | DRD2 | protein_coding | Transcript |
| rs2734837 | 11:113416107-113416107 | intron_variant,non_coding_transcript_variant | DRD2 | retained_intron | Transcript |
| rs2734837 | 11:113416107-113416107 | intron_variant,non_coding_transcript_variant | DRD2 | retained_intron | Transcript |
| rs2734837 | 11:113416107-113416107 | intron_variant | DRD2 | protein_coding | Transcript |
| rs2734837 | 11:113416107-113416107 | downstream_gene_variant | DRD2 | protein_coding | Transcript |
| rs2734837 | 11:113416107-113416107 | intron_variant | DRD2 | protein_coding | Transcript |
| rs2734837 | 11:113416107-113416107 | downstream_gene_variant | - | lncRNA | Transcript |
| rs13220522 | 6:26316067-26316067 | intron_variant,non_coding_transcript_variant | - | TEC | Transcript |
| rs13220522 | 6:26316067-26316067 | intron_variant,non_coding_transcript_variant | - | TEC | Transcript |
| rs13220522 | 6:26316067-26316067 | intron_variant,non_coding_transcript_variant | - | TEC | Transcript |
| rs13220522 | 6:26316067-26316067 | intron_variant,non_coding_transcript_variant | - | TEC | Transcript |
| rs13220522 | 6:26316067-26316067 | intron_variant,non_coding_transcript_variant | - | lncRNA | Transcript |
| rs13220522 | 6:26316067-26316067 | intron_variant,non_coding_transcript_variant | - | lncRNA | Transcript |
| rs13220522 | 6:26316067-26316067 | intron_variant,non_coding_transcript_variant | - | lncRNA | Transcript |
| rs13220522 | 6:26316067-26316067 | intron_variant,non_coding_transcript_variant | - | lncRNA | Transcript |
| ***Major depression and preterm birth*** | | | | |  |
| rs149543464 | 6:30432986-30432986 | intergenic_variant | - | - | - |
| rs149543464 | 6:30432986-30432986 | intergenic_variant | - | - | - |
| rs57440165 | 6:26875738-26875738 | downstream_gene_variant | - | lncRNA | Transcript |
| rs57440165 | 6:26875738-26875738 | intron_variant,non_coding_transcript_variant | - | lncRNA | Transcript |
| rs57440165 | 6:26875738-26875738 | downstream_gene_variant | GUSBP2 | transcribed_unprocessed_pseudogene | Transcript |
| rs57440165 | 6:26875738-26875738 | intron_variant,non_coding_transcript_variant | - | lncRNA | Transcript |
| rs57440165 | 6:26875738-26875738 | intron_variant,non_coding_transcript_variant | - | lncRNA | Transcript |
| rs57440165 | 6:26875738-26875738 | intron_variant,non_coding_transcript_variant | - | lncRNA | Transcript |
| rs57440165 | 6:26875738-26875738 | intron_variant,non_coding_transcript_variant | - | lncRNA | Transcript |
| rs57440165 | 6:26875738-26875738 | intron_variant,non_coding_transcript_variant | - | lncRNA | Transcript |
| rs57440165 | 6:26875738-26875738 | intron_variant,non_coding_transcript_variant | - | lncRNA | Transcript |
| rs57440165 | 6:26875738-26875738 | intron_variant,non_coding_transcript_variant | - | lncRNA | Transcript |
| rs57440165 | 6:26875738-26875738 | intron_variant,non_coding_transcript_variant | - | lncRNA | Transcript |
| rs57440165 | 6:26875738-26875738 | intron_variant,non_coding_transcript_variant | - | lncRNA | Transcript |
| rs57440165 | 6:26875738-26875738 | intron_variant,non_coding_transcript_variant | - | lncRNA | Transcript |
| rs57440165 | 6:26875738-26875738 | intron_variant,non_coding_transcript_variant | - | lncRNA | Transcript |
| rs57440165 | 6:26875738-26875738 | intron_variant,non_coding_transcript_variant | - | lncRNA | Transcript |
| rs57440165 | 6:26875738-26875738 | intron_variant,non_coding_transcript_variant | - | lncRNA | Transcript |
| rs57440165 | 6:26875738-26875738 | intron_variant,non_coding_transcript_variant | - | lncRNA | Transcript |
| rs57440165 | 6:26875738-26875738 | intron_variant,non_coding_transcript_variant | - | lncRNA | Transcript |
| rs57440165 | 6:26875738-26875738 | intron_variant,non_coding_transcript_variant | - | lncRNA | Transcript |
| rs57440165 | 6:26875738-26875738 | intron_variant,non_coding_transcript_variant | - | lncRNA | Transcript |
| rs57440165 | 6:26875738-26875738 | intron_variant,non_coding_transcript_variant | - | lncRNA | Transcript |
| rs57440165 | 6:26875738-26875738 | intron_variant,non_coding_transcript_variant | - | lncRNA | Transcript |
| rs57440165 | 6:26875738-26875738 | intron_variant,non_coding_transcript_variant | - | lncRNA | Transcript |
| rs57440165 | 6:26875738-26875738 | intron_variant,non_coding_transcript_variant | - | lncRNA | Transcript |
| rs57440165 | 6:26875738-26875738 | intron_variant,non_coding_transcript_variant | - | lncRNA | Transcript |
| rs57440165 | 6:26875738-26875738 | intron_variant,non_coding_transcript_variant | - | lncRNA | Transcript |
| rs57440165 | 6:26875738-26875738 | intron_variant,non_coding_transcript_variant | - | lncRNA | Transcript |
| rs57440165 | 6:26875738-26875738 | intron_variant,non_coding_transcript_variant | - | lncRNA | Transcript |
| rs57440165 | 6:26875738-26875738 | intron_variant,non_coding_transcript_variant | - | lncRNA | Transcript |
| rs57440165 | 6:26875738-26875738 | intron_variant,non_coding_transcript_variant | - | lncRNA | Transcript |
| rs57440165 | 6:26875738-26875738 | intron_variant,non_coding_transcript_variant | - | lncRNA | Transcript |
| rs57440165 | 6:26875738-26875738 | intron_variant,non_coding_transcript_variant | - | lncRNA | Transcript |
| rs57440165 | 6:26875738-26875738 | intron_variant,non_coding_transcript_variant | - | lncRNA | Transcript |
| rs57440165 | 6:26875738-26875738 | intron_variant,non_coding_transcript_variant | - | lncRNA | Transcript |
| rs57440165 | 6:26875738-26875738 | intron_variant,non_coding_transcript_variant | - | lncRNA | Transcript |
| rs57440165 | 6:26875738-26875738 | downstream_gene_variant | - | lncRNA | Transcript |
| rs57440165 | 6:26875738-26875738 | downstream_gene_variant | - | lncRNA | Transcript |
| rs57440165 | 6:26875738-26875738 | downstream_gene_variant | - | lncRNA | Transcript |
| rs57440165 | 6:26875738-26875738 | downstream_gene_variant | - | lncRNA | Transcript |
| rs57440165 | 6:26875738-26875738 | downstream_gene_variant | - | lncRNA | Transcript |
| rs57440165 | 6:26875738-26875738 | downstream_gene_variant | - | lncRNA | Transcript |
| rs57440165 | 6:26875738-26875738 | intron_variant,non_coding_transcript_variant | - | lncRNA | Transcript |
| rs57440165 | 6:26875738-26875738 | intron_variant,non_coding_transcript_variant | - | lncRNA | Transcript |
| rs57440165 | 6:26875738-26875738 | intron_variant,non_coding_transcript_variant | - | lncRNA | Transcript |
| rs57440165 | 6:26875738-26875738 | intron_variant,non_coding_transcript_variant | - | lncRNA | Transcript |
| rs57440165 | 6:26875738-26875738 | intron_variant,non_coding_transcript_variant | - | lncRNA | Transcript |
| rs57440165 | 6:26875738-26875738 | intron_variant,non_coding_transcript_variant | - | lncRNA | Transcript |
| rs57440165 | 6:26875738-26875738 | intron_variant,non_coding_transcript_variant | - | lncRNA | Transcript |
| rs57440165 | 6:26875738-26875738 | downstream_gene_variant | - | lncRNA | Transcript |
| rs57440165 | 6:26875738-26875738 | intron_variant,non_coding_transcript_variant | - | lncRNA | Transcript |
| rs57440165 | 6:26875738-26875738 | intron_variant,non_coding_transcript_variant | - | lncRNA | Transcript |
| rs57440165 | 6:26875738-26875738 | intron_variant,non_coding_transcript_variant | - | lncRNA | Transcript |
| rs57440165 | 6:26875738-26875738 | intron_variant,non_coding_transcript_variant | - | lncRNA | Transcript |
| rs57440165 | 6:26875738-26875738 | intron_variant,non_coding_transcript_variant | - | lncRNA | Transcript |
| rs57440165 | 6:26875738-26875738 | intron_variant,non_coding_transcript_variant | - | lncRNA | Transcript |
| rs57440165 | 6:26875738-26875738 | downstream_gene_variant | - | lncRNA | Transcript |
| rs57440165 | 6:26875738-26875738 | intron_variant,non_coding_transcript_variant | - | lncRNA | Transcript |
| rs57440165 | 6:26875738-26875738 | intron_variant,non_coding_transcript_variant | - | lncRNA | Transcript |
| rs57440165 | 6:26875738-26875738 | downstream_gene_variant | - | lncRNA | Transcript |
| rs57440165 | 6:26875738-26875738 | downstream_gene_variant | - | lncRNA | Transcript |
| ***Bipolar disease and preterm birth*** | | | | |  |
| rs60476972 | 16:9058806-9058806 | intron_variant,non_coding_transcript_variant | - | lncRNA | Transcript |
| rs7813444 | 8:64524949-64524949 | intergenic_variant | - | - | - |
| rs7813444 | 8:64524949-64524949 | intergenic_variant | - | - | - |
| rs9273363 | 6:32658495-32658495 | downstream_gene_variant | HLA-DQB1 | protein_coding | Transcript |
| rs9273363 | 6:32658495-32658495 | downstream_gene_variant | HLA-DQB1 | protein_coding | Transcript |
| rs9273363 | 6:32658495-32658495 | downstream_gene_variant | HLA-DQB1 | protein_coding | Transcript |
| rs9273363 | 6:32658495-32658495 | downstream_gene_variant | HLA-DQB1 | protein_coding | Transcript |
| rs9273363 | 6:32658495-32658495 | downstream_gene_variant | HLA-DQB1 | protein_coding | Transcript |
| rs9273363 | 6:32658495-32658495 | downstream_gene_variant | HLA-DQB1 | protein_coding | Transcript |
| rs9273363 | 6:32658495-32658495 | downstream_gene_variant | HLA-DQB1 | protein_coding | Transcript |
| rs9273363 | 6:32658495-32658495 | downstream_gene_variant | HLA-DQB1 | protein_coding | Transcript |
| rs9273363 | 6:32658495-32658495 | upstream_gene_variant | HLA-DQB1 | lncRNA | Transcript |
| rs9273363 | 6:32658495-32658495 | upstream_gene_variant | HLA-DQB1 | lncRNA | Transcript |
| rs9273363 | 6:32658495-32658495 | downstream_gene_variant | HLA-DQB1 | protein_coding | Transcript |
| rs9273363 | 6:32658495-32658495 | downstream_gene_variant | HLA-DQB1 | protein_coding | Transcript |
| rs9273363 | 6:32658495-32658495 | downstream_gene_variant | HLA-DQB1 | protein_coding_CDS_not_defined | Transcript |
| rs9273363 | 6:32658495-32658495 | downstream_gene_variant | HLA-DQB1 | protein_coding_CDS_not_defined | Transcript |
| rs9273363 | 6:32658495-32658495 | downstream_gene_variant | HLA-DQB1 | protein_coding_CDS_not_defined | Transcript |
| rs9273363 | 6:32658495-32658495 | downstream_gene_variant | HLA-DQB1 | protein_coding_CDS_not_defined | Transcript |
| rs9273363 | 6:32658495-32658495 | downstream_gene_variant | HLA-DQB1 | nonsense_mediated_decay | Transcript |
| rs9273363 | 6:32658495-32658495 | downstream_gene_variant | HLA-DQB1 | nonsense_mediated_decay | Transcript |
| rs9273363 | 6:32658495-32658495 | downstream_gene_variant | HLA-DQB1 | retained_intron | Transcript |
| rs9273363 | 6:32658495-32658495 | downstream_gene_variant | HLA-DQB1 | retained_intron | Transcript |
| rs1264349 | 6:30828882-30828882 | intron_variant,non_coding_transcript_variant | LINC00243 | lncRNA | Transcript |
| rs1264349 | 6:30828882-30828882 | intron_variant,non_coding_transcript_variant | LINC00243 | lncRNA | Transcript |
| rs1264349 | 6:30828882-30828882 | intron_variant,non_coding_transcript_variant | LINC00243 | lncRNA | Transcript |
| rs1264349 | 6:30828882-30828882 | intron_variant,non_coding_transcript_variant | LINC00243 | lncRNA | Transcript |
| rs1264349 | 6:30828882-30828882 | downstream_gene_variant | LINC00243 | lncRNA | Transcript |
| rs1264349 | 6:30828882-30828882 | regulatory_region_variant | - | enhancer | RegulatoryFeature |
| rs3132948 | 6:32229442-32229442 | intergenic_variant | - | - | - |
| rs3132948 | 6:32229442-32229442 | intergenic_variant | - | - | - |

**Table S8. Genes that interact with the shared SNPs for broad depression and PTB through 3D chromatin loops in different cell types.**

| **Gene** | **Loop type** | **Loop start** | **Loop end** | **Cell type** | **Tissue** |
| --- | --- | --- | --- | --- | --- |
| ***Index SNP: rs2734837*** | | | | | |
| ANKK1 | Within loop | chr11:113190722-113200722 | chr11:113340722-113350722 | NHEK | Skin |
| ANKK1 | Within loop | chr11:113190722-113200722 | chr11:113340722-113350722 | HUVEC | Blood vessel |
| ANKK1 | Within loop | chr11:113220722-113230722 | chr11:113400722-113410722 | HUVEC | Blood vessel |
| ANKK1 | Within loop | chr11:113230722-113240722 | chr11:113390722-113400722 | HMEC | Breast |
| ANKK1 | Within loop | chr11:113230722-113240722 | chr11:113400722-113410722 | NHEK | Skin |
| ANKK1 | Within loop | chr11:113200722-113210722 | chr11:113290722-113300722 | Psoas |  |
| ANKK1 | Within loop | chr11:113170000-113180000 | chr11:113360000-113370000 | K562 | Blood |
| ANKK1 | Within loop | chr11:113190722-113200722 | chr11:113310722-113320722 | NHEK | Skin |
| ANKK1 | Within loop | chr11:113170722-113180722 | chr11:113370722-113380722 | T47D |  |
| ANKK1 | Within loop | chr11:113170722-113180722 | chr11:113370722-113380722 | RPMI7951 |  |
| DRD2 | Within loop | chr11:113190722-113200722 | chr11:113340722-113350722 | NHEK | Skin |
| DRD2 | Within loop | chr11:113190722-113200722 | chr11:113340722-113350722 | HUVEC | Blood vessel |
| DRD2 | Within loop | chr11:113220722-113230722 | chr11:113400722-113410722 | HUVEC | Blood vessel |
| DRD2 | Within loop | chr11:113230722-113240722 | chr11:113390722-113400722 | HMEC | Breast |
| DRD2 | Within loop | chr11:113230722-113240722 | chr11:113400722-113410722 | NHEK | Skin |
| DRD2 | Within loop | chr11:113170000-113180000 | chr11:113360000-113370000 | K562 | Blood |
| DRD2 | Within loop | chr11:113170722-113180722 | chr11:113370722-113380722 | T47D |  |
| DRD2 | Within loop | chr11:113170722-113180722 | chr11:113370722-113380722 | RPMI7951 |  |
| MIR4301 | Within loop | chr11:113190722-113200722 | chr11:113340722-113350722 | NHEK | Skin |
| MIR4301 | Within loop | chr11:113190722-113200722 | chr11:113340722-113350722 | HUVEC | Blood vessel |
| MIR4301 | Within loop | chr11:113220722-113230722 | chr11:113400722-113410722 | HUVEC | Blood vessel |
| MIR4301 | Within loop | chr11:113230722-113240722 | chr11:113390722-113400722 | HMEC | Breast |
| MIR4301 | Within loop | chr11:113230722-113240722 | chr11:113400722-113410722 | NHEK | Skin |
| MIR4301 | Within loop | chr11:113170000-113180000 | chr11:113360000-113370000 | K562 | Blood |
| MIR4301 | Within loop | chr11:113170722-113180722 | chr11:113370722-113380722 | T47D |  |
| MIR4301 | Within loop | chr11:113170722-113180722 | chr11:113370722-113380722 | RPMI7951 |  |
| TTC12 | Within loop | chr11:113170000-113180000 | chr11:113360000-113370000 | K562 | Blood |
| TTC12 | Within loop | chr11:113170722-113180722 | chr11:113370722-113380722 | T47D |  |
| TTC12 | Within loop | chr11:113170722-113180722 | chr11:113370722-113380722 | RPMI7951 |  |
| ***Index SNP: rs13220522*** | | | | | |
| BTN2A1 | Within loop | chr6:26310228-26320228 | chr6:26460228-26470228 | NCIH460 |  |
| BTN2A1 | Within loop | chr6:26300228-26310228 | chr6:26480228-26490228 | Aorta |  |
| BTN2A2 | Within loop | chr6:26310228-26320228 | chr6:26460228-26470228 | NCIH460 |  |
| BTN2A2 | Within loop | chr6:26270228-26280228 | chr6:26440228-26450228 | Hippocampus |  |
| BTN2A2 | Within loop | chr6:26290228-26300228 | chr6:26430228-26440228 | Spleen |  |
| BTN2A2 | Within loop | chr6:26300228-26310228 | chr6:26480228-26490228 | Aorta |  |
| BTN2A3P | Within loop | chr6:26310228-26320228 | chr6:26460228-26470228 | NCIH460 |  |
| BTN2A3P | Within loop | chr6:26270228-26280228 | chr6:26440228-26450228 | Hippocampus |  |
| BTN2A3P | Within loop | chr6:26290228-26300228 | chr6:26430228-26440228 | Spleen |  |
| BTN2A3P | Within loop | chr6:26300228-26310228 | chr6:26480228-26490228 | Aorta |  |
| BTN3A1 | Within loop | chr6:26310228-26320228 | chr6:26460228-26470228 | NCIH460 |  |
| BTN3A1 | Within loop | chr6:26270228-26280228 | chr6:26440228-26450228 | Hippocampus |  |
| BTN3A1 | Within loop | chr6:26290228-26300228 | chr6:26430228-26440228 | Spleen |  |
| BTN3A1 | Within loop | chr6:26300228-26310228 | chr6:26480228-26490228 | Aorta |  |
| BTN3A2 | Within loop | chr6:26310228-26320228 | chr6:26460228-26470228 | NCIH460 |  |
| BTN3A2 | Within loop | chr6:26270228-26280228 | chr6:26440228-26450228 | Hippocampus |  |
| BTN3A2 | Within loop | chr6:26290228-26300228 | chr6:26430228-26440228 | Spleen |  |
| BTN3A2 | Within loop | chr6:26300228-26310228 | chr6:26480228-26490228 | Aorta |  |
| BTN3A2 | Within loop | chr6:26250228-26260228 | chr6:26370228-26380228 | Adrenal |  |
| BTN3A3 | Within loop | chr6:26310228-26320228 | chr6:26460228-26470228 | NCIH460 |  |
| BTN3A3 | Within loop | chr6:26270228-26280228 | chr6:26440228-26450228 | Hippocampus |  |
| BTN3A3 | Within loop | chr6:26300228-26310228 | chr6:26480228-26490228 | Aorta |  |
| HIST1H1D | Within loop | chr6:26220228-26230228 | chr6:26310228-26320228 | HepG2 | Liver |
| HIST1H1D | Within loop | chr6:26220228-26230228 | chr6:26320228-26330228 | G401 |  |
| HIST1H1D | Within loop | chr6:26210228-26220228 | chr6:26310228-26320228 | Pancreas |  |
| HIST1H1D | Within loop | chr6:26210228-26220228 | chr6:26310228-26320228 | Hippocampus |  |
| HIST1H1D | Within loop | chr6:26210228-26220228 | chr6:26310228-26320228 | RPMI7951 |  |
| HIST1H1D | Within loop | chr6:26210228-26220228 | chr6:26310228-26320228 | PANC1 |  |
| HIST1H1D | Within loop | chr6:26210228-26220228 | chr6:26310228-26320228 | Caki2 |  |
| HIST1H1D | Within loop | chr6:26230228-26240228 | chr6:26320228-26330228 | SKMEL5 |  |
| HIST1H1D | Within loop | chr6:26190228-26200228 | chr6:26310228-26320228 | SKMEL5 |  |
| HIST1H1D | Within loop | chr6:26210000-26220000 | chr6:26320000-26330000 | K562 | Blood |
| HIST1H1D | Within loop | chr6:26190228-26200228 | chr6:26330228-26340228 | G401 |  |
| HIST1H1D | Within loop | chr6:26200228-26210228 | chr6:26310228-26320228 | NHEK | Skin |
| HIST1H1D | Within loop | chr6:26200228-26210228 | chr6:26310228-26320228 | HMEC | Breast |
| HIST1H1D | Within loop | chr6:26180228-26190228 | chr6:26310228-26320228 | Bowel_Small |  |
| HIST1H2AD | Within loop | chr6:26190228-26200228 | chr6:26310228-26320228 | SKMEL5 |  |
| HIST1H2AD | Within loop | chr6:26190228-26200228 | chr6:26330228-26340228 | G401 |  |
| HIST1H2AD | Within loop | chr6:26180228-26190228 | chr6:26310228-26320228 | Bowel_Small |  |
| HIST1H2AE | Within loop | chr6:26210228-26220228 | chr6:26310228-26320228 | Pancreas |  |
| HIST1H2AE | Within loop | chr6:26210228-26220228 | chr6:26310228-26320228 | Hippocampus |  |
| HIST1H2AE | Within loop | chr6:26210228-26220228 | chr6:26310228-26320228 | RPMI7951 |  |
| HIST1H2AE | Within loop | chr6:26210228-26220228 | chr6:26310228-26320228 | PANC1 |  |
| HIST1H2AE | Within loop | chr6:26210228-26220228 | chr6:26310228-26320228 | Caki2 |  |
| HIST1H2AE | Within loop | chr6:26190228-26200228 | chr6:26310228-26320228 | SKMEL5 |  |
| HIST1H2AE | Within loop | chr6:26210000-26220000 | chr6:26320000-26330000 | K562 | Blood |
| HIST1H2AE | Within loop | chr6:26190228-26200228 | chr6:26330228-26340228 | G401 |  |
| HIST1H2AE | Within loop | chr6:26200228-26210228 | chr6:26310228-26320228 | NHEK | Skin |
| HIST1H2AE | Within loop | chr6:26200228-26210228 | chr6:26310228-26320228 | HMEC | Breast |
| HIST1H2AE | Within loop | chr6:26180228-26190228 | chr6:26310228-26320228 | Bowel_Small |  |
| HIST1H2AH | Anchor to anchor | chr6:26310228-26320228 | chr6:27107779-27117779 | HCT116-untreated |  |
| HIST1H2BE | Within loop | chr6:26180228-26190228 | chr6:26310228-26320228 | Bowel_Small |  |
| HIST1H2BF | Within loop | chr6:26190228-26200228 | chr6:26310228-26320228 | SKMEL5 |  |
| HIST1H2BF | Within loop | chr6:26190228-26200228 | chr6:26330228-26340228 | G401 |  |
| HIST1H2BF | Within loop | chr6:26180228-26190228 | chr6:26310228-26320228 | Bowel_Small |  |
| HIST1H2BG | Within loop | chr6:26210228-26220228 | chr6:26310228-26320228 | Pancreas |  |
| HIST1H2BG | Within loop | chr6:26210228-26220228 | chr6:26310228-26320228 | Hippocampus |  |
| HIST1H2BG | Within loop | chr6:26210228-26220228 | chr6:26310228-26320228 | RPMI7951 |  |
| HIST1H2BG | Within loop | chr6:26210228-26220228 | chr6:26310228-26320228 | PANC1 |  |
| HIST1H2BG | Within loop | chr6:26210228-26220228 | chr6:26310228-26320228 | Caki2 |  |
| HIST1H2BG | Within loop | chr6:26190228-26200228 | chr6:26310228-26320228 | SKMEL5 |  |
| HIST1H2BG | Within loop | chr6:26210000-26220000 | chr6:26320000-26330000 | K562 | Blood |
| HIST1H2BG | Within loop | chr6:26190228-26200228 | chr6:26330228-26340228 | G401 |  |
| HIST1H2BG | Within loop | chr6:26200228-26210228 | chr6:26310228-26320228 | NHEK | Skin |
| HIST1H2BG | Within loop | chr6:26200228-26210228 | chr6:26310228-26320228 | HMEC | Breast |
| HIST1H2BG | Within loop | chr6:26180228-26190228 | chr6:26310228-26320228 | Bowel_Small |  |
| HIST1H2BH | Within loop | chr6:26220228-26230228 | chr6:26310228-26320228 | HepG2 | Liver |
| HIST1H2BH | Within loop | chr6:26220228-26230228 | chr6:26320228-26330228 | G401 |  |
| HIST1H2BH | Within loop | chr6:26250228-26260228 | chr6:26330228-26340228 | RPMI7951 |  |
| HIST1H2BH | Within loop | chr6:26240228-26250228 | chr6:26320228-26330228 | Pancreas |  |
| HIST1H2BH | Within loop | chr6:26240228-26250228 | chr6:26320228-26330228 | NCIH460 |  |
| HIST1H2BH | Within loop | chr6:26210228-26220228 | chr6:26310228-26320228 | Pancreas |  |
| HIST1H2BH | Within loop | chr6:26210228-26220228 | chr6:26310228-26320228 | Hippocampus |  |
| HIST1H2BH | Within loop | chr6:26210228-26220228 | chr6:26310228-26320228 | RPMI7951 |  |
| HIST1H2BH | Within loop | chr6:26210228-26220228 | chr6:26310228-26320228 | PANC1 |  |
| HIST1H2BH | Within loop | chr6:26210228-26220228 | chr6:26310228-26320228 | Caki2 |  |
| HIST1H2BH | Within loop | chr6:26230228-26240228 | chr6:26320228-26330228 | SKMEL5 |  |
| HIST1H2BH | Within loop | chr6:26190228-26200228 | chr6:26310228-26320228 | SKMEL5 |  |
| HIST1H2BH | Within loop | chr6:26250228-26260228 | chr6:26370228-26380228 | Adrenal |  |
| HIST1H2BH | Within loop | chr6:26210000-26220000 | chr6:26320000-26330000 | K562 | Blood |
| HIST1H2BH | Within loop | chr6:26190228-26200228 | chr6:26330228-26340228 | G401 |  |
| HIST1H2BH | Within loop | chr6:26200228-26210228 | chr6:26310228-26320228 | NHEK | Skin |
| HIST1H2BH | Within loop | chr6:26200228-26210228 | chr6:26310228-26320228 | HMEC | Breast |
| HIST1H2BH | Within loop | chr6:26180228-26190228 | chr6:26310228-26320228 | Bowel_Small |  |
| HIST1H2BI | Within loop | chr6:26220228-26230228 | chr6:26310228-26320228 | HepG2 | Liver |
| HIST1H2BI | Within loop | chr6:26220228-26230228 | chr6:26320228-26330228 | G401 |  |
| HIST1H2BI | Within loop | chr6:26250228-26260228 | chr6:26330228-26340228 | RPMI7951 |  |
| HIST1H2BI | Within loop | chr6:26240228-26250228 | chr6:26320228-26330228 | Pancreas |  |
| HIST1H2BI | Within loop | chr6:26240228-26250228 | chr6:26320228-26330228 | NCIH460 |  |
| HIST1H2BI | Within loop | chr6:26210228-26220228 | chr6:26310228-26320228 | Pancreas |  |
| HIST1H2BI | Within loop | chr6:26210228-26220228 | chr6:26310228-26320228 | Hippocampus |  |
| HIST1H2BI | Within loop | chr6:26210228-26220228 | chr6:26310228-26320228 | RPMI7951 |  |
| HIST1H2BI | Within loop | chr6:26210228-26220228 | chr6:26310228-26320228 | PANC1 |  |
| HIST1H2BI | Within loop | chr6:26210228-26220228 | chr6:26310228-26320228 | Caki2 |  |
| HIST1H2BI | Within loop | chr6:26270228-26280228 | chr6:26440228-26450228 | Hippocampus |  |
| HIST1H2BI | Within loop | chr6:26230228-26240228 | chr6:26320228-26330228 | SKMEL5 |  |
| HIST1H2BI | Within loop | chr6:26190228-26200228 | chr6:26310228-26320228 | SKMEL5 |  |
| HIST1H2BI | Within loop | chr6:26250228-26260228 | chr6:26370228-26380228 | Adrenal |  |
| HIST1H2BI | Within loop | chr6:26210000-26220000 | chr6:26320000-26330000 | K562 | Blood |
| HIST1H2BI | Within loop | chr6:26190228-26200228 | chr6:26330228-26340228 | G401 |  |
| HIST1H2BI | Within loop | chr6:26200228-26210228 | chr6:26310228-26320228 | NHEK | Skin |
| HIST1H2BI | Within loop | chr6:26200228-26210228 | chr6:26310228-26320228 | HMEC | Breast |
| HIST1H2BI | Within loop | chr6:26180228-26190228 | chr6:26310228-26320228 | Bowel_Small |  |
| HIST1H2BK | Anchor to anchor | chr6:26310228-26320228 | chr6:27107779-27117779 | HCT116-untreated |  |
| HIST1H3D | Within loop | chr6:26190228-26200228 | chr6:26310228-26320228 | SKMEL5 |  |
| HIST1H3D | Within loop | chr6:26190228-26200228 | chr6:26330228-26340228 | G401 |  |
| HIST1H3D | Within loop | chr6:26180228-26190228 | chr6:26310228-26320228 | Bowel_Small |  |
| HIST1H3E | Within loop | chr6:26220228-26230228 | chr6:26310228-26320228 | HepG2 | Liver |
| HIST1H3E | Within loop | chr6:26220228-26230228 | chr6:26320228-26330228 | G401 |  |
| HIST1H3E | Within loop | chr6:26210228-26220228 | chr6:26310228-26320228 | Pancreas |  |
| HIST1H3E | Within loop | chr6:26210228-26220228 | chr6:26310228-26320228 | Hippocampus |  |
| HIST1H3E | Within loop | chr6:26210228-26220228 | chr6:26310228-26320228 | RPMI7951 |  |
| HIST1H3E | Within loop | chr6:26210228-26220228 | chr6:26310228-26320228 | PANC1 |  |
| HIST1H3E | Within loop | chr6:26210228-26220228 | chr6:26310228-26320228 | Caki2 |  |
| HIST1H3E | Within loop | chr6:26190228-26200228 | chr6:26310228-26320228 | SKMEL5 |  |
| HIST1H3E | Within loop | chr6:26210000-26220000 | chr6:26320000-26330000 | K562 | Blood |
| HIST1H3E | Within loop | chr6:26190228-26200228 | chr6:26330228-26340228 | G401 |  |
| HIST1H3E | Within loop | chr6:26200228-26210228 | chr6:26310228-26320228 | NHEK | Skin |
| HIST1H3E | Within loop | chr6:26200228-26210228 | chr6:26310228-26320228 | HMEC | Breast |
| HIST1H3E | Within loop | chr6:26180228-26190228 | chr6:26310228-26320228 | Bowel_Small |  |
| HIST1H3F | Within loop | chr6:26220228-26230228 | chr6:26310228-26320228 | HepG2 | Liver |
| HIST1H3F | Within loop | chr6:26220228-26230228 | chr6:26320228-26330228 | G401 |  |
| HIST1H3F | Within loop | chr6:26250228-26260228 | chr6:26330228-26340228 | RPMI7951 |  |
| HIST1H3F | Within loop | chr6:26240228-26250228 | chr6:26320228-26330228 | Pancreas |  |
| HIST1H3F | Within loop | chr6:26240228-26250228 | chr6:26320228-26330228 | NCIH460 |  |
| HIST1H3F | Within loop | chr6:26210228-26220228 | chr6:26310228-26320228 | Pancreas |  |
| HIST1H3F | Within loop | chr6:26210228-26220228 | chr6:26310228-26320228 | Hippocampus |  |
| HIST1H3F | Within loop | chr6:26210228-26220228 | chr6:26310228-26320228 | RPMI7951 |  |
| HIST1H3F | Within loop | chr6:26210228-26220228 | chr6:26310228-26320228 | PANC1 |  |
| HIST1H3F | Within loop | chr6:26210228-26220228 | chr6:26310228-26320228 | Caki2 |  |
| HIST1H3F | Within loop | chr6:26230228-26240228 | chr6:26320228-26330228 | SKMEL5 |  |
| HIST1H3F | Within loop | chr6:26190228-26200228 | chr6:26310228-26320228 | SKMEL5 |  |
| HIST1H3F | Within loop | chr6:26250228-26260228 | chr6:26370228-26380228 | Adrenal |  |
| HIST1H3F | Within loop | chr6:26210000-26220000 | chr6:26320000-26330000 | K562 | Blood |
| HIST1H3F | Within loop | chr6:26190228-26200228 | chr6:26330228-26340228 | G401 |  |
| HIST1H3F | Within loop | chr6:26200228-26210228 | chr6:26310228-26320228 | NHEK | Skin |
| HIST1H3F | Within loop | chr6:26200228-26210228 | chr6:26310228-26320228 | HMEC | Breast |
| HIST1H3F | Within loop | chr6:26180228-26190228 | chr6:26310228-26320228 | Bowel_Small |  |
| HIST1H3G | Within loop | chr6:26220228-26230228 | chr6:26310228-26320228 | HepG2 | Liver |
| HIST1H3G | Within loop | chr6:26220228-26230228 | chr6:26320228-26330228 | G401 |  |
| HIST1H3G | Within loop | chr6:26250228-26260228 | chr6:26330228-26340228 | RPMI7951 |  |
| HIST1H3G | Within loop | chr6:26240228-26250228 | chr6:26320228-26330228 | Pancreas |  |
| HIST1H3G | Within loop | chr6:26240228-26250228 | chr6:26320228-26330228 | NCIH460 |  |
| HIST1H3G | Within loop | chr6:26210228-26220228 | chr6:26310228-26320228 | Pancreas |  |
| HIST1H3G | Within loop | chr6:26210228-26220228 | chr6:26310228-26320228 | Hippocampus |  |
| HIST1H3G | Within loop | chr6:26210228-26220228 | chr6:26310228-26320228 | RPMI7951 |  |
| HIST1H3G | Within loop | chr6:26210228-26220228 | chr6:26310228-26320228 | PANC1 |  |
| HIST1H3G | Within loop | chr6:26210228-26220228 | chr6:26310228-26320228 | Caki2 |  |
| HIST1H3G | Within loop | chr6:26270228-26280228 | chr6:26440228-26450228 | Hippocampus |  |
| HIST1H3G | Within loop | chr6:26230228-26240228 | chr6:26320228-26330228 | SKMEL5 |  |
| HIST1H3G | Within loop | chr6:26190228-26200228 | chr6:26310228-26320228 | SKMEL5 |  |
| HIST1H3G | Within loop | chr6:26250228-26260228 | chr6:26370228-26380228 | Adrenal |  |
| HIST1H3G | Within loop | chr6:26210000-26220000 | chr6:26320000-26330000 | K562 | Blood |
| HIST1H3G | Within loop | chr6:26190228-26200228 | chr6:26330228-26340228 | G401 |  |
| HIST1H3G | Within loop | chr6:26200228-26210228 | chr6:26310228-26320228 | NHEK | Skin |
| HIST1H3G | Within loop | chr6:26200228-26210228 | chr6:26310228-26320228 | HMEC | Breast |
| HIST1H3G | Within loop | chr6:26180228-26190228 | chr6:26310228-26320228 | Bowel_Small |  |
| HIST1H4D | Within loop | chr6:26180228-26190228 | chr6:26310228-26320228 | Bowel_Small |  |
| HIST1H4E | Within loop | chr6:26190228-26200228 | chr6:26310228-26320228 | SKMEL5 |  |
| HIST1H4E | Within loop | chr6:26190228-26200228 | chr6:26330228-26340228 | G401 |  |
| HIST1H4E | Within loop | chr6:26200228-26210228 | chr6:26310228-26320228 | NHEK | Skin |
| HIST1H4E | Within loop | chr6:26200228-26210228 | chr6:26310228-26320228 | HMEC | Breast |
| HIST1H4E | Within loop | chr6:26180228-26190228 | chr6:26310228-26320228 | Bowel_Small |  |
| HIST1H4F | Within loop | chr6:26220228-26230228 | chr6:26310228-26320228 | HepG2 | Liver |
| HIST1H4F | Within loop | chr6:26220228-26230228 | chr6:26320228-26330228 | G401 |  |
| HIST1H4F | Within loop | chr6:26240228-26250228 | chr6:26320228-26330228 | Pancreas |  |
| HIST1H4F | Within loop | chr6:26240228-26250228 | chr6:26320228-26330228 | NCIH460 |  |
| HIST1H4F | Within loop | chr6:26210228-26220228 | chr6:26310228-26320228 | Pancreas |  |
| HIST1H4F | Within loop | chr6:26210228-26220228 | chr6:26310228-26320228 | Hippocampus |  |
| HIST1H4F | Within loop | chr6:26210228-26220228 | chr6:26310228-26320228 | RPMI7951 |  |
| HIST1H4F | Within loop | chr6:26210228-26220228 | chr6:26310228-26320228 | PANC1 |  |
| HIST1H4F | Within loop | chr6:26210228-26220228 | chr6:26310228-26320228 | Caki2 |  |
| HIST1H4F | Within loop | chr6:26230228-26240228 | chr6:26320228-26330228 | SKMEL5 |  |
| HIST1H4F | Within loop | chr6:26190228-26200228 | chr6:26310228-26320228 | SKMEL5 |  |
| HIST1H4F | Within loop | chr6:26210000-26220000 | chr6:26320000-26330000 | K562 | Blood |
| HIST1H4F | Within loop | chr6:26190228-26200228 | chr6:26330228-26340228 | G401 |  |
| HIST1H4F | Within loop | chr6:26200228-26210228 | chr6:26310228-26320228 | NHEK | Skin |
| HIST1H4F | Within loop | chr6:26200228-26210228 | chr6:26310228-26320228 | HMEC | Breast |
| HIST1H4F | Within loop | chr6:26180228-26190228 | chr6:26310228-26320228 | Bowel_Small |  |
| HIST1H4G | Within loop | chr6:26220228-26230228 | chr6:26310228-26320228 | HepG2 | Liver |
| HIST1H4G | Within loop | chr6:26220228-26230228 | chr6:26320228-26330228 | G401 |  |
| HIST1H4G | Within loop | chr6:26240228-26250228 | chr6:26320228-26330228 | Pancreas |  |
| HIST1H4G | Within loop | chr6:26240228-26250228 | chr6:26320228-26330228 | NCIH460 |  |
| HIST1H4G | Within loop | chr6:26210228-26220228 | chr6:26310228-26320228 | Pancreas |  |
| HIST1H4G | Within loop | chr6:26210228-26220228 | chr6:26310228-26320228 | Hippocampus |  |
| HIST1H4G | Within loop | chr6:26210228-26220228 | chr6:26310228-26320228 | RPMI7951 |  |
| HIST1H4G | Within loop | chr6:26210228-26220228 | chr6:26310228-26320228 | PANC1 |  |
| HIST1H4G | Within loop | chr6:26210228-26220228 | chr6:26310228-26320228 | Caki2 |  |
| HIST1H4G | Within loop | chr6:26230228-26240228 | chr6:26320228-26330228 | SKMEL5 |  |
| HIST1H4G | Within loop | chr6:26190228-26200228 | chr6:26310228-26320228 | SKMEL5 |  |
| HIST1H4G | Within loop | chr6:26210000-26220000 | chr6:26320000-26330000 | K562 | Blood |
| HIST1H4G | Within loop | chr6:26190228-26200228 | chr6:26330228-26340228 | G401 |  |
| HIST1H4G | Within loop | chr6:26200228-26210228 | chr6:26310228-26320228 | NHEK | Skin |
| HIST1H4G | Within loop | chr6:26200228-26210228 | chr6:26310228-26320228 | HMEC | Breast |
| HIST1H4G | Within loop | chr6:26180228-26190228 | chr6:26310228-26320228 | Bowel_Small |  |
| HIST1H4H | Within loop | chr6:26220228-26230228 | chr6:26310228-26320228 | HepG2 | Liver |
| HIST1H4H | Within loop | chr6:26220228-26230228 | chr6:26320228-26330228 | G401 |  |
| HIST1H4H | Within loop | chr6:26250228-26260228 | chr6:26330228-26340228 | RPMI7951 |  |
| HIST1H4H | Within loop | chr6:26240228-26250228 | chr6:26320228-26330228 | Pancreas |  |
| HIST1H4H | Within loop | chr6:26240228-26250228 | chr6:26320228-26330228 | NCIH460 |  |
| HIST1H4H | Within loop | chr6:26210228-26220228 | chr6:26310228-26320228 | Pancreas |  |
| HIST1H4H | Within loop | chr6:26210228-26220228 | chr6:26310228-26320228 | Hippocampus |  |
| HIST1H4H | Within loop | chr6:26210228-26220228 | chr6:26310228-26320228 | RPMI7951 |  |
| HIST1H4H | Within loop | chr6:26210228-26220228 | chr6:26310228-26320228 | PANC1 |  |
| HIST1H4H | Within loop | chr6:26210228-26220228 | chr6:26310228-26320228 | Caki2 |  |
| HIST1H4H | Within loop | chr6:26270228-26280228 | chr6:26440228-26450228 | Hippocampus |  |
| HIST1H4H | Within loop | chr6:26230228-26240228 | chr6:26320228-26330228 | SKMEL5 |  |
| HIST1H4H | Within loop | chr6:26190228-26200228 | chr6:26310228-26320228 | SKMEL5 |  |
| HIST1H4H | Within loop | chr6:26250228-26260228 | chr6:26370228-26380228 | Adrenal |  |
| HIST1H4H | Within loop | chr6:26210000-26220000 | chr6:26320000-26330000 | K562 | Blood |
| HIST1H4H | Within loop | chr6:26190228-26200228 | chr6:26330228-26340228 | G401 |  |
| HIST1H4H | Within loop | chr6:26200228-26210228 | chr6:26310228-26320228 | NHEK | Skin |
| HIST1H4H | Within loop | chr6:26200228-26210228 | chr6:26310228-26320228 | HMEC | Breast |
| HIST1H4H | Within loop | chr6:26180228-26190228 | chr6:26310228-26320228 | Bowel_Small |  |
| LOC285819 | Within loop | chr6:26300228-26310228 | chr6:26480228-26490228 | Aorta |  |
| MIR3143 | Anchor to anchor | chr6:26310228-26320228 | chr6:27107779-27117779 | HCT116-untreated |  |

**Table S9. Genes that interact with the shared SNPs for major depression and PTB through 3D chromatin loops in different cell types.**

| **Gene** | **Loop type** | **Loop start** | **Loop end** | **Cell type** | **Tissue** |
| --- | --- | --- | --- | --- | --- |
| ***Index SNP: rs149543464*** | | | | | |
| GNL1 | Within loop | chr6:30327777-30337777 | chr6:30517777-30527777 | NHEK | Skin |
| HLA-E | Within loop | chr6:30327777-30337777 | chr6:30517777-30527777 | NHEK | Skin |
| PRR3 | Within loop | chr6:30327777-30337777 | chr6:30517777-30527777 | NHEK | Skin |

**Table S10. Genes that interact with the shared SNPs for bipolar disease and PTB through 3D chromatin loops in different cell types.**

| **Gene** | **Loop type** | **Loop start** | **Loop end** | **Cell type** | **Tissue** |
| --- | --- | --- | --- | --- | --- |
| ***Index SNP: rs60476972*** | | | | | |
| C16orf72 | Within loop | chr16:9130000-9140000 | chr16:9210000-9220000 | K562 | Blood |
| C16orf72 | Within loop | chr16:9033857-9043857 | chr16:9193857-9203857 | Spleen |  |
| C16orf72 | Within loop | chr16:9033857-9043857 | chr16:9193857-9203857 | Epidermal_Keratinocyte_day3 |  |
| C16orf72 | Within loop | chr16:9003857-9013857 | chr16:9203857-9213857 | HUVEC | Blood vessel |
| C16orf72 | Within loop | chr16:9113857-9123857 | chr16:9213857-9223857 | HAP1 |  |
| C16orf72 | Within loop | chr16:9123857-9133857 | chr16:9213857-9223857 | HepG2 | Liver |
| C16orf72 | Within loop | chr16:9043857-9053857 | chr16:9213857-9223857 | IMR90 | Lung |
| C16orf72 | Within loop | chr16:9043857-9053857 | chr16:9193857-9203857 | KBM7 |  |
| C16orf72 | Within loop | chr16:9043857-9053857 | chr16:9193857-9203857 | HMEC | Breast |
| C16orf72 | Within loop | chr16:9043857-9053857 | chr16:9193857-9203857 | THP1_PMA |  |
| C16orf72 | Within loop | chr16:9043857-9053857 | chr16:9193857-9203857 | Liver |  |
| C16orf72 | Within loop | chr16:9023857-9033857 | chr16:9203857-9213857 | G401 |  |
| C16orf72 | Within loop | chr16:9063857-9073857 | chr16:9193857-9203857 | Thymus |  |
| C16orf72 | Within loop | chr16:9093857-9103857 | chr16:9203857-9213857 | THP1_PMA |  |
| C16orf72 | Within loop | chr16:8993857-9003857 | chr16:9193857-9203857 | Thymus |  |
| C16orf72 | Within loop | chr16:9123857-9133857 | chr16:9203857-9213857 | HUVEC | Blood vessel |
| C16orf72 | Within loop | chr16:9013857-9023857 | chr16:9203857-9213857 | HepG2 | Liver |
| C16orf72 | Within loop | chr16:9043857-9053857 | chr16:9203857-9213857 | HepG2 | Liver |
| USP7 | Within loop | chr16:9033857-9043857 | chr16:9193857-9203857 | Spleen |  |
| USP7 | Within loop | chr16:9033857-9043857 | chr16:9193857-9203857 | Epidermal_Keratinocyte_day3 |  |
| USP7 | Within loop | chr16:9003857-9013857 | chr16:9203857-9213857 | HUVEC | Blood vessel |
| USP7 | Within loop | chr16:9043857-9053857 | chr16:9213857-9223857 | IMR90 | Lung |
| USP7 | Within loop | chr16:9043857-9053857 | chr16:9193857-9203857 | KBM7 |  |
| USP7 | Within loop | chr16:9043857-9053857 | chr16:9193857-9203857 | HMEC | Breast |
| USP7 | Within loop | chr16:9043857-9053857 | chr16:9193857-9203857 | THP1_PMA |  |
| USP7 | Within loop | chr16:9043857-9053857 | chr16:9193857-9203857 | Liver |  |
| USP7 | Within loop | chr16:9023857-9033857 | chr16:9203857-9213857 | G401 |  |
| USP7 | Within loop | chr16:8993857-9003857 | chr16:9193857-9203857 | Thymus |  |
| USP7 | Within loop | chr16:9013857-9023857 | chr16:9203857-9213857 | HepG2 | Liver |
| USP7 | Within loop | chr16:9043857-9053857 | chr16:9203857-9213857 | HepG2 | Liver |
| ***Index SNP: rs7813444*** | | | | | |
| BHLHE22 | Within loop | chr8:65422557-65432557 | chr8:65602557-65612557 | VentricleLeft |  |
| BHLHE22 | Within loop | chr8:65432557-65442557 | chr8:65522557-65532557 | SKMEL5 |  |
| LOC401463 | Within loop | chr8:65422557-65432557 | chr8:65602557-65612557 | VentricleLeft |  |
| LOC401463 | Within loop | chr8:65432557-65442557 | chr8:65522557-65532557 | SKMEL5 |  |
| ***Index SNP: rs3132948*** | | | | | |
| AGER | Within loop | chr6:32077777-32087777 | chr6:32187777-32197777 | THP1 |  |
| AGER | Within loop | chr6:32077777-32087777 | chr6:32217777-32227777 | KBM7 |  |
| AGER | Within loop | chr6:32087777-32097777 | chr6:32207777-32217777 | THP1_PMA |  |
| AGER | Within loop | chr6:32037777-32047777 | chr6:32197777-32207777 | G401 |  |
| AGER | Within loop | chr6:32080000-32090000 | chr6:32210000-32220000 | K562 | Blood |
| AGPAT1 | Within loop | chr6:32077777-32087777 | chr6:32187777-32197777 | THP1 |  |
| AGPAT1 | Within loop | chr6:32077777-32087777 | chr6:32217777-32227777 | KBM7 |  |
| AGPAT1 | Within loop | chr6:32087777-32097777 | chr6:32207777-32217777 | THP1_PMA |  |
| AGPAT1 | Within loop | chr6:32037777-32047777 | chr6:32197777-32207777 | G401 |  |
| AGPAT1 | Within loop | chr6:32080000-32090000 | chr6:32210000-32220000 | K562 | Blood |
| ATF6B | Within loop | chr6:32077777-32087777 | chr6:32187777-32197777 | THP1 |  |
| ATF6B | Within loop | chr6:32077777-32087777 | chr6:32217777-32227777 | KBM7 |  |
| ATF6B | Within loop | chr6:32087777-32097777 | chr6:32207777-32217777 | THP1_PMA |  |
| ATF6B | Within loop | chr6:32037777-32047777 | chr6:32197777-32207777 | G401 |  |
| ATF6B | Within loop | chr6:32080000-32090000 | chr6:32210000-32220000 | K562 | Blood |
| BTNL2 | Within loop | chr6:32187777-32197777 | chr6:32377777-32387777 | PANC1 |  |
| BTNL2 | Within loop | chr6:32187777-32197777 | chr6:32367777-32377777 | Ventricle_Right |  |
| C6orf10 | Within loop | chr6:32187777-32197777 | chr6:32357777-32367777 | KBM7 |  |
| C6orf10 | Within loop | chr6:32170000-32180000 | chr6:32360000-32370000 | K562 | Blood |
| C6orf10 | Within loop | chr6:32187777-32197777 | chr6:32377777-32387777 | PANC1 |  |
| C6orf10 | Within loop | chr6:32187777-32197777 | chr6:32367777-32377777 | Ventricle_Right |  |
| EGFL8 | Within loop | chr6:32077777-32087777 | chr6:32187777-32197777 | THP1 |  |
| EGFL8 | Within loop | chr6:32077777-32087777 | chr6:32217777-32227777 | KBM7 |  |
| EGFL8 | Within loop | chr6:32087777-32097777 | chr6:32207777-32217777 | THP1_PMA |  |
| EGFL8 | Within loop | chr6:32037777-32047777 | chr6:32197777-32207777 | G401 |  |
| EGFL8 | Within loop | chr6:32080000-32090000 | chr6:32210000-32220000 | K562 | Blood |
| FKBPL | Within loop | chr6:32077777-32087777 | chr6:32187777-32197777 | THP1 |  |
| FKBPL | Within loop | chr6:32077777-32087777 | chr6:32217777-32227777 | KBM7 |  |
| FKBPL | Within loop | chr6:32087777-32097777 | chr6:32207777-32217777 | THP1_PMA |  |
| FKBPL | Within loop | chr6:32037777-32047777 | chr6:32197777-32207777 | G401 |  |
| FKBPL | Within loop | chr6:32080000-32090000 | chr6:32210000-32220000 | K562 | Blood |
| GPSM3 | Within loop | chr6:32077777-32087777 | chr6:32187777-32197777 | THP1 |  |
| GPSM3 | Within loop | chr6:32077777-32087777 | chr6:32217777-32227777 | KBM7 |  |
| GPSM3 | Within loop | chr6:32087777-32097777 | chr6:32207777-32217777 | THP1_PMA |  |
| GPSM3 | Within loop | chr6:32037777-32047777 | chr6:32197777-32207777 | G401 |  |
| GPSM3 | Within loop | chr6:32080000-32090000 | chr6:32210000-32220000 | K562 | Blood |
| HCG23 | Within loop | chr6:32187777-32197777 | chr6:32357777-32367777 | KBM7 |  |
| HCG23 | Within loop | chr6:32170000-32180000 | chr6:32360000-32370000 | K562 | Blood |
| HCG23 | Within loop | chr6:32187777-32197777 | chr6:32377777-32387777 | PANC1 |  |
| HCG23 | Within loop | chr6:32187777-32197777 | chr6:32367777-32377777 | Ventricle_Right |  |
| LOC100507547 | Within loop | chr6:32077777-32087777 | chr6:32187777-32197777 | THP1 |  |
| LOC100507547 | Within loop | chr6:32077777-32087777 | chr6:32217777-32227777 | KBM7 |  |
| LOC100507547 | Within loop | chr6:32087777-32097777 | chr6:32207777-32217777 | THP1_PMA |  |
| LOC100507547 | Within loop | chr6:32037777-32047777 | chr6:32197777-32207777 | G401 |  |
| LOC100507547 | Within loop | chr6:32080000-32090000 | chr6:32210000-32220000 | K562 | Blood |
| NOTCH4 | Within loop | chr6:32077777-32087777 | chr6:32187777-32197777 | THP1 |  |
| NOTCH4 | Within loop | chr6:32077777-32087777 | chr6:32217777-32227777 | KBM7 |  |
| NOTCH4 | Within loop | chr6:32087777-32097777 | chr6:32207777-32217777 | THP1_PMA |  |
| NOTCH4 | Within loop | chr6:32187777-32197777 | chr6:32357777-32367777 | KBM7 |  |
| NOTCH4 | Within loop | chr6:32177777-32187777 | chr6:32317777-32327777 | Spleen |  |
| NOTCH4 | Within loop | chr6:32037777-32047777 | chr6:32197777-32207777 | G401 |  |
| NOTCH4 | Within loop | chr6:32170000-32180000 | chr6:32360000-32370000 | K562 | Blood |
| NOTCH4 | Within loop | chr6:32080000-32090000 | chr6:32210000-32220000 | K562 | Blood |
| NOTCH4 | Within loop | chr6:32187777-32197777 | chr6:32327777-32337777 | PANC1 |  |
| NOTCH4 | Within loop | chr6:32187777-32197777 | chr6:32377777-32387777 | PANC1 |  |
| NOTCH4 | Within loop | chr6:32187777-32197777 | chr6:32367777-32377777 | Ventricle_Right |  |
| NOTCH4 | Anchor to anchor | chr6:32187777-32197777 | chr6:32657777-32667777 | Ventricle_Right |  |
| NOTCH4 | Anchor to anchor | chr6:32187777-32197777 | chr6:32767777-32777777 | Spleen |  |
| PBX2 | Within loop | chr6:32077777-32087777 | chr6:32187777-32197777 | THP1 |  |
| PBX2 | Within loop | chr6:32077777-32087777 | chr6:32217777-32227777 | KBM7 |  |
| PBX2 | Within loop | chr6:32087777-32097777 | chr6:32207777-32217777 | THP1_PMA |  |
| PBX2 | Within loop | chr6:32037777-32047777 | chr6:32197777-32207777 | G401 |  |
| PBX2 | Within loop | chr6:32080000-32090000 | chr6:32210000-32220000 | K562 | Blood |
| PPT2 | Within loop | chr6:32077777-32087777 | chr6:32187777-32197777 | THP1 |  |
| PPT2 | Within loop | chr6:32077777-32087777 | chr6:32217777-32227777 | KBM7 |  |
| PPT2 | Within loop | chr6:32087777-32097777 | chr6:32207777-32217777 | THP1_PMA |  |
| PPT2 | Within loop | chr6:32037777-32047777 | chr6:32197777-32207777 | G401 |  |
| PPT2 | Within loop | chr6:32080000-32090000 | chr6:32210000-32220000 | K562 | Blood |
| PPT2-EGFL8 | Within loop | chr6:32077777-32087777 | chr6:32187777-32197777 | THP1 |  |
| PPT2-EGFL8 | Within loop | chr6:32077777-32087777 | chr6:32217777-32227777 | KBM7 |  |
| PPT2-EGFL8 | Within loop | chr6:32087777-32097777 | chr6:32207777-32217777 | THP1_PMA |  |
| PPT2-EGFL8 | Within loop | chr6:32037777-32047777 | chr6:32197777-32207777 | G401 |  |
| PPT2-EGFL8 | Within loop | chr6:32080000-32090000 | chr6:32210000-32220000 | K562 | Blood |
| PRRT1 | Within loop | chr6:32077777-32087777 | chr6:32187777-32197777 | THP1 |  |
| PRRT1 | Within loop | chr6:32077777-32087777 | chr6:32217777-32227777 | KBM7 |  |
| PRRT1 | Within loop | chr6:32087777-32097777 | chr6:32207777-32217777 | THP1_PMA |  |
| PRRT1 | Within loop | chr6:32037777-32047777 | chr6:32197777-32207777 | G401 |  |
| PRRT1 | Within loop | chr6:32080000-32090000 | chr6:32210000-32220000 | K562 | Blood |
| RNF5 | Within loop | chr6:32077777-32087777 | chr6:32187777-32197777 | THP1 |  |
| RNF5 | Within loop | chr6:32077777-32087777 | chr6:32217777-32227777 | KBM7 |  |
| RNF5 | Within loop | chr6:32087777-32097777 | chr6:32207777-32217777 | THP1_PMA |  |
| RNF5 | Within loop | chr6:32037777-32047777 | chr6:32197777-32207777 | G401 |  |
| RNF5 | Within loop | chr6:32080000-32090000 | chr6:32210000-32220000 | K562 | Blood |
| RNF5P1 | Within loop | chr6:32077777-32087777 | chr6:32187777-32197777 | THP1 |  |
| RNF5P1 | Within loop | chr6:32077777-32087777 | chr6:32217777-32227777 | KBM7 |  |
| RNF5P1 | Within loop | chr6:32087777-32097777 | chr6:32207777-32217777 | THP1_PMA |  |
| RNF5P1 | Within loop | chr6:32037777-32047777 | chr6:32197777-32207777 | G401 |  |
| RNF5P1 | Within loop | chr6:32080000-32090000 | chr6:32210000-32220000 | K562 | Blood |
| TNXB | Within loop | chr6:32037777-32047777 | chr6:32197777-32207777 | G401 |  |
| ***Index SNP: rs9273363*** | | | | | |
| HLA-DQA1 | Within loop | chr6:32537777-32547777 | chr6:32647777-32657777 | THP1_PMA |  |
| HLA-DQA1 | Within loop | chr6:32567777-32577777 | chr6:32707777-32717777 | RPMI7951 |  |
| HLA-DQA1 | Within loop | chr6:32550000-32560000 | chr6:32660000-32670000 | GM12878 | Blood |
| HLA-DQA1 | Within loop | chr6:32577777-32587777 | chr6:32697777-32707777 | T47D |  |
| HLA-DQA1 | Within loop | chr6:32587777-32597777 | chr6:32717777-32727777 | H1-NPC |  |
| HLA-DQA1 | Within loop | chr6:32587777-32597777 | chr6:32757777-32767777 | Epidermal_Keratinocyte_day0 |  |
| HLA-DQA1 | Within loop | chr6:32587777-32597777 | chr6:32757777-32767777 | THP1 |  |
| HLA-DQA1 | Within loop | chr6:32577777-32587777 | chr6:32657777-32667777 | PANC1 |  |
| HLA-DQA2 | Within loop | chr6:32567777-32577777 | chr6:32707777-32717777 | RPMI7951 |  |
| HLA-DQA2 | Within loop | chr6:32617777-32627777 | chr6:32767777-32777777 | Spleen |  |
| HLA-DQA2 | Within loop | chr6:32587777-32597777 | chr6:32717777-32727777 | H1-NPC |  |
| HLA-DQA2 | Within loop | chr6:32607777-32617777 | chr6:32717777-32727777 | Aorta |  |
| HLA-DQA2 | Within loop | chr6:32587777-32597777 | chr6:32757777-32767777 | Epidermal_Keratinocyte_day0 |  |
| HLA-DQA2 | Within loop | chr6:32587777-32597777 | chr6:32757777-32767777 | THP1 |  |
| HLA-DQB1 | Within loop | chr6:32537777-32547777 | chr6:32647777-32657777 | THP1_PMA |  |
| HLA-DQB1 | Within loop | chr6:32567777-32577777 | chr6:32707777-32717777 | RPMI7951 |  |
| HLA-DQB1 | Within loop | chr6:32617777-32627777 | chr6:32767777-32777777 | Spleen |  |
| HLA-DQB1 | Within loop | chr6:32550000-32560000 | chr6:32660000-32670000 | GM12878 | Blood |
| HLA-DQB1 | Within loop | chr6:32577777-32587777 | chr6:32697777-32707777 | T47D |  |
| HLA-DQB1 | Within loop | chr6:32587777-32597777 | chr6:32717777-32727777 | H1-NPC |  |
| HLA-DQB1 | Within loop | chr6:32607777-32617777 | chr6:32717777-32727777 | Aorta |  |
| HLA-DQB1 | Within loop | chr6:32587777-32597777 | chr6:32757777-32767777 | Epidermal_Keratinocyte_day0 |  |
| HLA-DQB1 | Within loop | chr6:32587777-32597777 | chr6:32757777-32767777 | THP1 |  |
| HLA-DQB1 | Within loop | chr6:32577777-32587777 | chr6:32657777-32667777 | PANC1 |  |
| HLA-DQB2 | Within loop | chr6:32617777-32627777 | chr6:32767777-32777777 | Spleen |  |
| HLA-DQB2 | Within loop | chr6:32587777-32597777 | chr6:32757777-32767777 | Epidermal_Keratinocyte_day0 |  |
| HLA-DQB2 | Within loop | chr6:32587777-32597777 | chr6:32757777-32767777 | THP1 |  |
| HLA-DRB1 | Within loop | chr6:32537777-32547777 | chr6:32647777-32657777 | THP1_PMA |  |
| HLA-DRB1 | Within loop | chr6:32550000-32560000 | chr6:32660000-32670000 | GM12878 | Blood |
| ***Index SNP: rs1264349*** | | | | | |
| C6orf15 | Anchor to anchor | chr6:30787777-30797777 | chr6:31077777-31087777 | HAP1_DKO |  |
| DDR1 | Within loop | chr6:30757777-30767777 | chr6:30847777-30857777 | Spleen |  |
| DDR1 | Within loop | chr6:30757777-30767777 | chr6:30847777-30857777 | T47D |  |
| DDR1 | Within loop | chr6:30757777-30767777 | chr6:30847777-30857777 | A549 | Epithelium |
| DDR1 | Within loop | chr6:30647777-30657777 | chr6:30847777-30857777 | HMEC | Breast |
| DDR1 | Within loop | chr6:30647777-30657777 | chr6:30847777-30857777 | HAP1_DKO |  |
| DDR1 | Within loop | chr6:30647777-30657777 | chr6:30847777-30857777 | HepG2 | Liver |
| DDR1 | Within loop | chr6:30787777-30797777 | chr6:30867777-30877777 | HCT116-untreated |  |
| DDR1 | Within loop | chr6:30787777-30797777 | chr6:30867777-30877777 | HepG2 | Liver |
| DDR1 | Within loop | chr6:30707777-30717777 | chr6:30847777-30857777 | HCT116-untreated |  |
| DDR1 | Within loop | chr6:30707777-30717777 | chr6:30847777-30857777 | KBM7 |  |
| DDR1 | Within loop | chr6:30707777-30717777 | chr6:30847777-30857777 | HMEC | Breast |
| DDR1 | Within loop | chr6:30707777-30717777 | chr6:30847777-30857777 | THP1 |  |
| DDR1 | Within loop | chr6:30707777-30717777 | chr6:30847777-30857777 | HepG2 | Liver |
| DDR1 | Within loop | chr6:30790000-30800000 | chr6:30950000-30960000 | K562 | Blood |
| DDR1 | Within loop | chr6:30687777-30697777 | chr6:30857777-30867777 | HAP1 |  |
| DDR1 | Within loop | chr6:30787777-30797777 | chr6:30877777-30887777 | HMEC | Breast |
| DDR1 | Within loop | chr6:30687777-30697777 | chr6:30847777-30857777 | HAP1_DKO |  |
| DDR1 | Within loop | chr6:30687777-30697777 | chr6:30847777-30857777 | Caki2 |  |
| DDR1 | Within loop | chr6:30790000-30800000 | chr6:30870000-30880000 | K562 | Blood |
| DDR1 | Within loop | chr6:30790000-30800000 | chr6:30870000-30880000 | GM12878 | Blood |
| DDR1 | Within loop | chr6:30777777-30787777 | chr6:30857777-30867777 | LNCAP |  |
| DDR1 | Within loop | chr6:30697777-30707777 | chr6:30857777-30867777 | HAP1_WAPL |  |
| DDR1 | Within loop | chr6:30697777-30707777 | chr6:30847777-30857777 | HUVEC | Blood vessel |
| DDR1 | Within loop | chr6:30697777-30707777 | chr6:30847777-30857777 | HAP1_SCC4_KO |  |
| DDR1 | Within loop | chr6:30697777-30707777 | chr6:30847777-30857777 | G401 |  |
| DPCR1 | Within loop | chr6:30790000-30800000 | chr6:30950000-30960000 | K562 | Blood |
| FLOT1 | Within loop | chr6:30700000-30710000 | chr6:30790000-30800000 | K562 | Blood |
| FLOT1 | Within loop | chr6:30647777-30657777 | chr6:30847777-30857777 | HMEC | Breast |
| FLOT1 | Within loop | chr6:30647777-30657777 | chr6:30847777-30857777 | HAP1_DKO |  |
| FLOT1 | Within loop | chr6:30647777-30657777 | chr6:30847777-30857777 | HepG2 | Liver |
| FLOT1 | Within loop | chr6:30710000-30720000 | chr6:30840000-30850000 | GM12878 | Blood |
| FLOT1 | Within loop | chr6:30697777-30707777 | chr6:30837777-30847777 | Hippocampus |  |
| FLOT1 | Within loop | chr6:30697777-30707777 | chr6:30837777-30847777 | IMR90 | Lung |
| FLOT1 | Within loop | chr6:30697777-30707777 | chr6:30837777-30847777 | T47D |  |
| FLOT1 | Within loop | chr6:30707777-30717777 | chr6:30847777-30857777 | HCT116-untreated |  |
| FLOT1 | Within loop | chr6:30707777-30717777 | chr6:30847777-30857777 | KBM7 |  |
| FLOT1 | Within loop | chr6:30707777-30717777 | chr6:30847777-30857777 | HMEC | Breast |
| FLOT1 | Within loop | chr6:30707777-30717777 | chr6:30847777-30857777 | THP1 |  |
| FLOT1 | Within loop | chr6:30707777-30717777 | chr6:30847777-30857777 | HepG2 | Liver |
| FLOT1 | Within loop | chr6:30687777-30697777 | chr6:30857777-30867777 | HAP1 |  |
| FLOT1 | Within loop | chr6:30687777-30697777 | chr6:30837777-30847777 | Astrocyte |  |
| FLOT1 | Within loop | chr6:30687777-30697777 | chr6:30847777-30857777 | HAP1_DKO |  |
| FLOT1 | Within loop | chr6:30687777-30697777 | chr6:30847777-30857777 | Caki2 |  |
| FLOT1 | Within loop | chr6:30697777-30707777 | chr6:30857777-30867777 | HAP1_WAPL |  |
| FLOT1 | Within loop | chr6:30707777-30717777 | chr6:30797777-30807777 | HAP1_WAPL |  |
| FLOT1 | Within loop | chr6:30700000-30710000 | chr6:30840000-30850000 | K562 | Blood |
| FLOT1 | Within loop | chr6:30707777-30717777 | chr6:30787777-30797777 | THP1 |  |
| FLOT1 | Within loop | chr6:30707777-30717777 | chr6:30787777-30797777 | HepG2 | Liver |
| FLOT1 | Within loop | chr6:30697777-30707777 | chr6:30847777-30857777 | HUVEC | Blood vessel |
| FLOT1 | Within loop | chr6:30697777-30707777 | chr6:30847777-30857777 | HAP1_SCC4_KO |  |
| FLOT1 | Within loop | chr6:30697777-30707777 | chr6:30847777-30857777 | G401 |  |
| FLOT1 | Within loop | chr6:30677777-30687777 | chr6:30837777-30847777 | PANC1 |  |
| GTF2H4 | Within loop | chr6:30787777-30797777 | chr6:30867777-30877777 | HCT116-untreated |  |
| GTF2H4 | Within loop | chr6:30787777-30797777 | chr6:30867777-30877777 | HepG2 | Liver |
| GTF2H4 | Within loop | chr6:30790000-30800000 | chr6:30950000-30960000 | K562 | Blood |
| GTF2H4 | Within loop | chr6:30787777-30797777 | chr6:30877777-30887777 | HMEC | Breast |
| GTF2H4 | Within loop | chr6:30790000-30800000 | chr6:30870000-30880000 | K562 | Blood |
| GTF2H4 | Within loop | chr6:30790000-30800000 | chr6:30870000-30880000 | GM12878 | Blood |
| IER3 | Within loop | chr6:30700000-30710000 | chr6:30790000-30800000 | K562 | Blood |
| IER3 | Within loop | chr6:30647777-30657777 | chr6:30847777-30857777 | HMEC | Breast |
| IER3 | Within loop | chr6:30647777-30657777 | chr6:30847777-30857777 | HAP1_DKO |  |
| IER3 | Within loop | chr6:30647777-30657777 | chr6:30847777-30857777 | HepG2 | Liver |
| IER3 | Within loop | chr6:30710000-30720000 | chr6:30840000-30850000 | GM12878 | Blood |
| IER3 | Within loop | chr6:30697777-30707777 | chr6:30837777-30847777 | Hippocampus |  |
| IER3 | Within loop | chr6:30697777-30707777 | chr6:30837777-30847777 | IMR90 | Lung |
| IER3 | Within loop | chr6:30697777-30707777 | chr6:30837777-30847777 | T47D |  |
| IER3 | Within loop | chr6:30707777-30717777 | chr6:30847777-30857777 | HCT116-untreated |  |
| IER3 | Within loop | chr6:30707777-30717777 | chr6:30847777-30857777 | KBM7 |  |
| IER3 | Within loop | chr6:30707777-30717777 | chr6:30847777-30857777 | HMEC | Breast |
| IER3 | Within loop | chr6:30707777-30717777 | chr6:30847777-30857777 | THP1 |  |
| IER3 | Within loop | chr6:30707777-30717777 | chr6:30847777-30857777 | HepG2 | Liver |
| IER3 | Within loop | chr6:30687777-30697777 | chr6:30857777-30867777 | HAP1 |  |
| IER3 | Within loop | chr6:30687777-30697777 | chr6:30837777-30847777 | Astrocyte |  |
| IER3 | Within loop | chr6:30687777-30697777 | chr6:30847777-30857777 | HAP1_DKO |  |
| IER3 | Within loop | chr6:30687777-30697777 | chr6:30847777-30857777 | Caki2 |  |
| IER3 | Within loop | chr6:30697777-30707777 | chr6:30857777-30867777 | HAP1_WAPL |  |
| IER3 | Within loop | chr6:30707777-30717777 | chr6:30797777-30807777 | HAP1_WAPL |  |
| IER3 | Within loop | chr6:30700000-30710000 | chr6:30840000-30850000 | K562 | Blood |
| IER3 | Within loop | chr6:30707777-30717777 | chr6:30787777-30797777 | THP1 |  |
| IER3 | Within loop | chr6:30707777-30717777 | chr6:30787777-30797777 | HepG2 | Liver |
| IER3 | Within loop | chr6:30697777-30707777 | chr6:30847777-30857777 | HUVEC | Blood vessel |
| IER3 | Within loop | chr6:30697777-30707777 | chr6:30847777-30857777 | HAP1_SCC4_KO |  |
| IER3 | Within loop | chr6:30697777-30707777 | chr6:30847777-30857777 | G401 |  |
| IER3 | Within loop | chr6:30677777-30687777 | chr6:30837777-30847777 | PANC1 |  |
| MDC1 | Within loop | chr6:30647777-30657777 | chr6:30847777-30857777 | HMEC | Breast |
| MDC1 | Within loop | chr6:30647777-30657777 | chr6:30847777-30857777 | HAP1_DKO |  |
| MDC1 | Within loop | chr6:30647777-30657777 | chr6:30847777-30857777 | HepG2 | Liver |
| MDC1 | Within loop | chr6:30677777-30687777 | chr6:30837777-30847777 | PANC1 |  |
| MIR4640 | Within loop | chr6:30787777-30797777 | chr6:30867777-30877777 | HCT116-untreated |  |
| MIR4640 | Within loop | chr6:30787777-30797777 | chr6:30867777-30877777 | HepG2 | Liver |
| MIR4640 | Within loop | chr6:30790000-30800000 | chr6:30950000-30960000 | K562 | Blood |
| MIR4640 | Within loop | chr6:30687777-30697777 | chr6:30857777-30867777 | HAP1 |  |
| MIR4640 | Within loop | chr6:30787777-30797777 | chr6:30877777-30887777 | HMEC | Breast |
| MIR4640 | Within loop | chr6:30790000-30800000 | chr6:30870000-30880000 | K562 | Blood |
| MIR4640 | Within loop | chr6:30790000-30800000 | chr6:30870000-30880000 | GM12878 | Blood |
| MIR4640 | Within loop | chr6:30777777-30787777 | chr6:30857777-30867777 | LNCAP |  |
| MIR4640 | Within loop | chr6:30697777-30707777 | chr6:30857777-30867777 | HAP1_WAPL |  |
| MUC21 | Within loop | chr6:30790000-30800000 | chr6:30950000-30960000 | K562 | Blood |
| NRM | Within loop | chr6:30647777-30657777 | chr6:30847777-30857777 | HMEC | Breast |
| NRM | Within loop | chr6:30647777-30657777 | chr6:30847777-30857777 | HAP1_DKO |  |
| NRM | Within loop | chr6:30647777-30657777 | chr6:30847777-30857777 | HepG2 | Liver |
| PPP1R18 | Within loop | chr6:30647777-30657777 | chr6:30847777-30857777 | HMEC | Breast |
| PPP1R18 | Within loop | chr6:30647777-30657777 | chr6:30847777-30857777 | HAP1_DKO |  |
| PPP1R18 | Within loop | chr6:30647777-30657777 | chr6:30847777-30857777 | HepG2 | Liver |
| PSORS1C1 | Anchor to anchor | chr6:30787777-30797777 | chr6:31077777-31087777 | HAP1_DKO |  |
| SFTA2 | Within loop | chr6:30790000-30800000 | chr6:30950000-30960000 | K562 | Blood |
| TUBB | Within loop | chr6:30647777-30657777 | chr6:30847777-30857777 | HMEC | Breast |
| TUBB | Within loop | chr6:30647777-30657777 | chr6:30847777-30857777 | HAP1_DKO |  |
| TUBB | Within loop | chr6:30647777-30657777 | chr6:30847777-30857777 | HepG2 | Liver |
| TUBB | Within loop | chr6:30687777-30697777 | chr6:30857777-30867777 | HAP1 |  |
| TUBB | Within loop | chr6:30687777-30697777 | chr6:30837777-30847777 | Astrocyte |  |
| TUBB | Within loop | chr6:30687777-30697777 | chr6:30847777-30857777 | HAP1_DKO |  |
| TUBB | Within loop | chr6:30687777-30697777 | chr6:30847777-30857777 | Caki2 |  |
| TUBB | Within loop | chr6:30677777-30687777 | chr6:30837777-30847777 | PANC1 |  |
| VARS2 | Within loop | chr6:30790000-30800000 | chr6:30950000-30960000 | K562 | Blood |
| VARS2 | Within loop | chr6:30787777-30797777 | chr6:30877777-30887777 | HMEC | Breast |

**Table S11. Significant SNP-gene pairs (FDR < 0.05) associated with the shared SNP for broad depression and PTB in 44 human tissues obtained from GTEx Portal**

| **Index SNP** | **Gene** | **P-value** | **Effect size** | **Tissue** |
| --- | --- | --- | --- | --- |
| rs2734837 | ANKK1 | 5.95643e-07 | 0.240 | Adipose Subcutaneous |
|  | ANKK1 | 2.66074e-07 | 0.239 | Adipose Visceral Omentum |
|  | ANKK1 | 4.10353e-10 | 0.341 | Artery Aorta |
|  | ANKK1 | 6.00316e-06 | 0.135 | Breast Mammary Tissue |
|  | ANKK1 | 2.0978e-07 | 0.290 | Colon Sigmoid |
|  | ANKK1 | 1.49224e-09 | 0.321 | Colon Transverse |
|  | ANKK1 | 7.78641e-06 | -0.201 | Esophagus Mucosa |
|  | ANKK1 | 7.52962e-08 | 0.290 | Heart Atrial Appendage |
|  | ANKK1 | 4.6245e-07 | 0.273 | Heart Left Ventricle |
|  | ANKK1 | 2.63088e-07 | 0.371 | Liver |
|  | ANKK1 | 2.47118e-14 | 0.242 | Lung |
|  | ANKK1 | 5.00658e-13 | 0.311 | Muscle Skeletal |
|  | ANKK1 | 4.49916e-23 | 0.483 | Nerve Tibial |
|  | ANKK1 | 3.0243e-08 | 0.374 | Pancreas |
|  | ANKK1 | 3.00625e-05 | 0.228 | Small Intestine Terminal Ileum |
|  | ANKK1 | 2.62177e-06 | 0.312 | Spleen |
|  | ANKK1 | 1.70514e-07 | 0.340 | Stomach |
|  | ANKK1 | 2.37668e-14 | 0.367 | Testis |
|  | ANKK1 | 9.57784e-05 | 0.138 | Thyroid |
|  | ANKK1 | 2.75997e-14 | 0.266 | Whole Blood |
|  | TTC12 | 2.86519e-21 | -0.421 | Adipose Subcutaneous |
|  | TTC12 | 5.49308e-21 | -0.432 | Adipose Visceral Omentum |
|  | TTC12 | 5.6122e-10 | -0.507 | Adrenal Gland |
|  | TTC12 | 7.2973e-12 | -0.333 | Artery Aorta |
|  | TTC12 | 1.30881e-06 | -0.345 | Artery Coronary |
|  | TTC12 | 4.62054e-23 | -0.436 | Artery Tibial |
|  | TTC12 | 5.38566e-06 | -0.402 | Brain Amygdala |
|  | TTC12 | 8.54755e-10 | -0.477 | Brain Anterior cingulate cortex BA24 |
|  | TTC12 | 5.5959e-10 | -0.494 | Brain Caudate basal ganglia |
|  | TTC12 | 3.04833e-06 | -0.328 | Brain Cerebellar Hemisphere |
|  | TTC12 | 6.00782e-06 | -0.338 | Brain Cerebellum |
|  | TTC12 | 4.28848e-11 | -0.463 | Brain Cortex |
|  | TTC12 | 4.08807e-10 | -0.472 | Brain Frontal Cortex BA9 |
|  | TTC12 | 2.54037e-06 | -0.358 | Brain Hippocampus |
|  | TTC12 | 4.81654e-07 | -0.385 | Brain Hypothalamus |
|  | TTC12 | 2.57781e-06 | -0.341 | Brain Nucleus accumbens basal ganglia |
|  | TTC12 | 2.82895e-06 | -0.404 | Brain Putamen basal ganglia |
|  | TTC12 | 9.06906e-19 | -0.327 | Breast Mammary Tissue |
|  | TTC12 | 1.24401e-05 | -0.254 | Cells Cultured fibroblasts |
|  | TTC12 | 4.7389e-17 | -0.455 | Colon Sigmoid |
|  | TTC12 | 1.0061e-11 | -0.370 | Colon Transverse |
|  | TTC12 | 2.43174e-23 | -0.545 | Esophagus Gastroesophageal Junction |
|  | TTC12 | 5.50655e-19 | -0.384 | Esophagus Mucosa |
|  | TTC12 | 1.75598e-24 | -0.528 | Esophagus Muscularis |
|  | TTC12 | 7.39398e-26 | -0.569 | Heart Atrial Appendage |
|  | TTC12 | 7.1907e-24 | -0.528 | Heart Left Ventricle |
|  | TTC12 | 2.75479e-06 | -0.617 | Kidney Cortex |
|  | TTC12 | 5.21026e-06 | -0.351 | Liver |
|  | TTC12 | 1.72985e-21 | -0.341 | Lung |
|  | TTC12 | 4.76479e-37 | -0.479 | Muscle Skeletal |
|  | TTC12 | 1.31554e-17 | -0.408 | Nerve Tibial |
|  | TTC12 | 6.19863e-12 | -0.471 | Pancreas |
|  | TTC12 | 1.85677e-12 | -0.563 | Pituitary |
|  | TTC12 | 4.66883e-05 | -0.320 | Prostate |
|  | TTC12 | 6.0897e-22 | -0.410 | Skin Not Sun Exposed Suprapubic |
|  | TTC12 | 7.26111e-22 | -0.395 | Skin Sun Exposed Lower leg |
|  | TTC12 | 1.90466e-06 | -0.377 | Small Intestine Terminal Ileum |
|  | TTC12 | 1.87208e-09 | -0.361 | Stomach |
|  | TTC12 | 5.28272e-05 | -0.133 | Testis |
|  | TTC12 | 1.29902e-18 | -0.413 | Thyroid |
|  | TTC12 | 4.79123e-13 | -0.218 | Whole Blood |
| rs13220522 | BTN2A2 | 3.01984e-06 | -0.709 | Pancreas |
|  | BTN2A2 | 2.01366e-07 | -0.321 | Skin Sun Exposed Lower leg |
|  | BTN3A2 | 6.59069e-42 | -1.012 | Adipose Subcutaneous |
|  | BTN3A2 | 1.28606e-37 | -1.006 | Adipose Visceral Omentum |
|  | BTN3A2 | 2.27295e-15 | -1.342 | Adrenal Gland |
|  | BTN3A2 | 2.54133e-40 | -1.127 | Artery Aorta |
|  | BTN3A2 | 2.50696e-22 | -1.030 | Artery Coronary |
|  | BTN3A2 | 5.84652e-56 | -0.962 | Artery Tibial |
|  | BTN3A2 | 5.43197e-08 | -0.954 | Brain Amygdala |
|  | BTN3A2 | 1.23788e-10 | -1.093 | Brain Anterior cingulate cortex BA24 |
|  | BTN3A2 | 1.07891e-12 | -1.055 | Brain Caudate basal ganglia |
|  | BTN3A2 | 9.98335e-10 | -1.103 | Brain Cerebellar Hemisphere |
|  | BTN3A2 | 1.22058e-11 | -1.103 | Brain Cerebellum |
|  | BTN3A2 | 7.11369e-15 | -1.051 | Brain Cortex |
|  | BTN3A2 | 3.08429e-10 | -0.946 | Brain Frontal Cortex BA9 |
|  | BTN3A2 | 4.99402e-09 | -1.045 | Brain Hippocampus |
|  | BTN3A2 | 7.15216e-09 | -1.020 | Brain Hypothalamus |
|  | BTN3A2 | 2.50227e-10 | -0.968 | Brain Nucleus accumbens basal ganglia |
|  | BTN3A2 | 1.44434e-08 | -1.045 | Brain Putamen basal ganglia |
|  | BTN3A2 | 3.23659e-11 | -1.236 | Brain Spinal cord cervical c-1 |
|  | BTN3A2 | 3.85487e-36 | -1.095 | Breast Mammary Tissue |
|  | BTN3A2 | 3.46016e-42 | -1.248 | Cells Cultured fibroblasts |
|  | BTN3A2 | 1.12279e-07 | -1.070 | Cells EBV-transformed lymphocytes |
|  | BTN3A2 | 4.19438e-21 | -0.957 | Colon Sigmoid |
|  | BTN3A2 | 1.8566e-36 | -0.978 | Colon Transverse |
|  | BTN3A2 | 8.09857e-35 | -1.096 | Esophagus Gastroesophageal Junction |
|  | BTN3A2 | 2.31038e-51 | -1.186 | Esophagus Mucosa |
|  | BTN3A2 | 7.86883e-53 | -1.187 | Esophagus Muscularis |
|  | BTN3A2 | 9.94628e-36 | -1.165 | Heart Atrial Appendage |
|  | BTN3A2 | 1.54465e-44 | -1.112 | Heart Left Ventricle |
|  | BTN3A2 | 1.36684e-14 | -1.052 | Liver |
|  | BTN3A2 | 2.03081e-49 | -1.158 | Lung |
|  | BTN3A2 | 2.52614e-08 | -1.073 | Minor Salivary Gland |
|  | BTN3A2 | 8.12138e-51 | -1.164 | Muscle Skeletal |
|  | BTN3A2 | 2.67561e-47 | -1.064 | Nerve Tibial |
|  | BTN3A2 | 1.52017e-11 | -1.204 | Ovary |
|  | BTN3A2 | 6.42314e-23 | -1.255 | Pancreas |
|  | BTN3A2 | 4.63311e-15 | -1.210 | Pituitary |
|  | BTN3A2 | 5.74806e-14 | -0.956 | Prostate |
|  | BTN3A2 | 2.9862e-57 | -1.335 | Skin Sun Exposed Lower leg |
|  | BTN3A2 | 1.03211e-07 | -0.628 | Small Intestine Terminal Ileum |
|  | BTN3A2 | 2.01074e-24 | -1.332 | Spleen |
|  | BTN3A2 | 1.38465e-37 | -1.087 | Stomach |
|  | BTN3A2 | 4.6197e-28 | -1.240 | Testis |
|  | BTN3A2 | 2.13029e-50 | -1.113 | Thyroid |
|  | BTN3A2 | 7.46229e-12 | -1.257 | Uterus |
|  | BTN3A2 | 2.69732e-15 | -1.258 | Vagina |
|  | BTN3A2 | 4.90743e-71 | -1.021 | Whole Blood |
|  | GUSBP2 | 1.16779e-4 | 0.419 | Esophagus Mucosa |
|  | HIST1H2BK | 1.91622e-4 | 0.315 | Adipose Subcutaneous |
|  | HIST1H2BK | 2.58799e-4 | 0.300 | Nerve Tibial |
|  | LINC00240 | 5.40454e-4 | 0.320 | Esophagus Mucosa |
|  | LINC00240 | 7.45209e-05 | 0.312 | Muscle Skeletal |
|  | LINC00240 | 1.92379e-06 | 0.467 | Skin Sun Exposed Lower leg |
|  | PRSS16 | 8.00214e-06 | 0.689 | Adrenal Gland |
|  | PRSS16 | 4.46665e-05 | 0.437 | Artery Aorta |
|  | PRSS16 | 1.42026e-09 | 0.644 | Artery Tibial |
|  | PRSS16 | 5.95448e-09 | 1.010 | Brain Cerebellar Hemisphere |
|  | PRSS16 | 1.40761e-09 | 0.947 | Brain Cerebellum |
|  | PRSS16 | 6.99854e-06 | 0.514 | Esophagus Muscularis |
|  | PRSS16 | 1.03761e-05 | 0.376 | Prostate |
|  | PRSS16 | 1.48928e-08 | 0.329 | Skin Sun Exposed Lower leg |
|  | PRSS16 | 3.95983e-06 | 0.670 | Spleen |
|  | PRSS16 | 1.43878e-4 | 0.303 | Testis |
|  | PRSS16 | 1.12864e-07 | 0.254 | Thyroid |
|  | TRIM38 | 3.88734e-05 | -0.194 | Whole Blood |
|  | U91328.19 | 7.5527e-07 | 0.336 | Whole Blood |

**Table S12. Significant SNP-gene pairs (FDR < 0.05) associated with the shared SNP for major depression and PTB in 44 human tissues obtained from GTEx Portal**

| **Index SNP** | **Gene** | **P-value** | **Effect size** | **Tissue** |
| --- | --- | --- | --- | --- |
| rs149543464 | CCHCR1 | 1.02404e-11 | 0.439 | Adipose Subcutaneous |
|  | CCHCR1 | 8.38222e-09 | 0.321 | Adipose Visceral Omentum |
|  | CCHCR1 | 3.49748e-07 | 0.384 | Artery Aorta |
|  | CCHCR1 | 9.54248e-07 | 0.286 | Artery Tibial |
|  | CCHCR1 | 5.49862e-06 | 0.482 | Brain Cerebellar Hemisphere |
|  | CCHCR1 | 2.31306e-08 | 0.741 | Brain Cerebellum |
|  | CCHCR1 | 1.49089e-06 | 0.286 | Breast Mammary Tissue |
|  | CCHCR1 | 6.82669e-08 | 0.281 | Colon Transverse |
|  | CCHCR1 | 7.72293e-05 | 0.284 | Esophagus Gastroesophageal Junction |
|  | CCHCR1 | 1.17553e-06 | 0.276 | Esophagus Muscularis |
|  | CCHCR1 | 3.50329e-06 | 0.353 | Heart Atrial Appendage |
|  | CCHCR1 | 9.46577e-12 | 0.402 | Lung |
|  | CCHCR1 | 2.75497e-13 | 0.443 | Nerve Tibial |
|  | CCHCR1 | 6.8742e-05 | 0.266 | Skin Not Sun Exposed Suprapubic |
|  | CCHCR1 | 1.66769e-04 | 0.224 | Skin Sun Exposed Lower leg |
|  | CCHCR1 | 1.21297e-11 | 0.385 | Thyroid |
|  | CCHCR1 | 2.4878e-09 | 0.286 | Whole Blood |
|  | DDR1-AS1 | 2.08611e-07 | 0.644 | Testis |
|  | FLOT1 | 1.38286e-05 | 0.178 | Adipose Subcutaneous |
|  | FLOT1 | 4.96317e-19 | 0.378 | Adipose Visceral Omentum |
|  | FLOT1 | 1.66959e-11 | 0.310 | Artery Aorta |
|  | FLOT1 | 1.62305e-06 | 0.166 | Artery Tibial |
|  | FLOT1 | 3.32258e-06 | 0.364 | Brain Caudate basal ganglia |
|  | FLOT1 | 2.10463e-08 | 0.508 | Brain Cerebellar Hemisphere |
|  | FLOT1 | 1.01969e-11 | 0.700 | Brain Cerebellum |
|  | FLOT1 | 3.53407e-07 | 0.394 | Brain Cortex |
|  | FLOT1 | 1.60748e-05 | 0.303 | Brain Hippocampus |
|  | FLOT1 | 1.78703e-07 | 0.375 | Brain Nucleus accumbens basal ganglia |
|  | FLOT1 | 1.8371e-07 | 0.204 | Breast Mammary Tissue |
|  | FLOT1 | 6.08304e-05 | 0.185 | Colon Transverse |
|  | FLOT1 | 1.13999e-05 | 0.253 | Esophagus Gastroesophageal Junction |
|  | FLOT1 | 1.48102e-07 | 0.239 | Esophagus Muscularis |
|  | FLOT1 | 3.17944e-15 | 0.272 | Lung |
|  | FLOT1 | 3.97249e-16 | 0.327 | Nerve Tibial |
|  | FLOT1 | 1.99616e-08 | 0.482 | Ovary |
|  | FLOT1 | 9.02824e-10 | 0.370 | Pancreas |
|  | FLOT1 | 1.02195e-12 | 0.479 | Pituitary |
|  | FLOT1 | 5.4083e-11 | 0.473 | Prostate |
|  | FLOT1 | 2.29358e-06 | 0.144 | Skin Not Sun Exposed Suprapubic |
|  | FLOT1 | 1.75988e-09 | 0.580 | Small Intestine Terminal Ileum |
|  | FLOT1 | 9.42184e-07 | 0.294 | Spleen |
|  | FLOT1 | 2.48947e-05 | 0.226 | Stomach |
|  | FLOT1 | 2.17286e-28 | 0.485 | Thyroid |
|  | FLOT1 | 7.68929e-05 | 0.077 | Whole Blood |
|  | HCG17 | 1.4909e-04 | -0.405 | Adipose Subcutaneous |
|  | HCG17 | 2.71201e-07 | -0.764 | Brain Caudate basal ganglia |
|  | HCG17 | 1.72058e-05 | -0.678 | Brain Cortex |
|  | HCG17 | 1.93115e-06 | -0.776 | Brain Putamen basal ganglia |
|  | HCG17 | 0.000279984 | -0.538 | Testis |
|  | HCG20 | 0.000154031 | -0.378 | Artery Tibial |
|  | HCG20 | 1.54193e-05 | -0.465 | Cells Cultured fibroblasts |
|  | HCG20 | 1.88865e-05 | -0.401 | Skin Sun Exposed Lower leg |
|  | HCG4 | 1.04888e-05 | -0.567 | Artery Aorta |
|  | HCG4 | 0.000215446 | -0.379 | Cells Cultured fibroblasts |
|  | HCG4 | 2.72476e-06 | -0.476 | Esophagus Mucosa |
|  | HCG4 | 1.35377e-05 | -0.472 | Lung |
|  | HCG4 | 0.000141567 | -0.346 | Skin Sun Exposed Lower leg |
|  | HCG4 | 1.76407e-05 | -0.715 | Spleen |
|  | HCG4 | 1.61705e-06 | -0.465 | Thyroid |
|  | HCG4B | 3.28581e-05 | 0.486 | Nerve Tibial |
|  | HCG4P3 | 6.08306e-05 | 0.359 | Adipose Subcutaneous |
|  | HCG4P3 | 1.84152e-05 | 0.365 | Adipose Visceral Omentum |
|  | HCG4P3 | 1.32904e-06 | 0.453 | Artery Tibial |
|  | HCG4P3 | 1.69335e-05 | 0.678 | Brain Frontal Cortex BA9 |
|  | HCG4P3 | 1.34834e-07 | 0.484 | Cells Cultured fibroblasts |
|  | HCG4P3 | 0.000175376 | 0.423 | Colon Transverse |
|  | HCG4P3 | 7.10111e-06 | 0.412 | Esophagus Mucosa |
|  | HCG4P3 | 1.7519e-05 | 0.409 | Esophagus Muscularis |
|  | HCG4P3 | 6.72809e-05 | 0.462 | Heart Atrial Appendage |
|  | HCG4P3 | 5.73013e-06 | 0.552 | Heart Left Ventricle |
|  | HCG4P3 | 5.2674e-09 | 0.523 | Nerve Tibial |
|  | HCG4P3 | 0.000117056 | 0.347 | Skin Not Sun Exposed Suprapubic |
|  | HCG4P3 | 6.15106e-06 | 0.368 | Skin Sun Exposed Lower leg |
|  | HCG4P3 | 0.00029291 | 0.270 | Thyroid |
|  | HCG4P5 | 0.000186429 | -0.302 | Skin Not Sun Exposed Suprapubic |
|  | HCG4P5 | 9.78308e-05 | -0.243 | Whole Blood |
|  | HCG4P7 | 0.000261578 | 0.412 | Esophagus Muscularis |
|  | HCG4P7 | 6.31182e-05 | 0.440 | Nerve Tibial |
|  | HCG4P7 | 0.000118459 | 0.705 | Prostate |
|  | HCP5B | 1.42644e-14 | 0.804 | Adipose Subcutaneous |
|  | HCP5B | 4.89096e-09 | 0.644 | Adipose Visceral Omentum |
|  | HCP5B | 8.19588e-10 | 1.023 | Adrenal Gland |
|  | HCP5B | 2.20412e-05 | 0.545 | Artery Aorta |
|  | HCP5B | 2.8483e-10 | 0.615 | Artery Tibial |
|  | HCP5B | 5.44921e-05 | 0.703 | Brain Putamen basal ganglia |
|  | HCP5B | 8.54115e-05 | 0.491 | Breast Mammary Tissue |
|  | HCP5B | 4.69326e-05 | 0.567 | Colon Sigmoid |
|  | HCP5B | 4.92178e-11 | 0.784 | Colon Transverse |
|  | HCP5B | 1.10214e-09 | 0.766 | Esophagus Gastroesophageal Junction |
|  | HCP5B | 3.79164e-10 | 0.612 | Esophagus Mucosa |
|  | HCP5B | 4.61743e-07 | 0.558 | Esophagus Muscularis |
|  | HCP5B | 5.72653e-10 | 0.906 | Liver |
|  | HCP5B | 1.49761e-14 | 0.761 | Lung |
|  | HCP5B | 7.79147e-07 | 0.427 | Muscle Skeletal |
|  | HCP5B | 3.51932e-13 | 0.763 | Nerve Tibial |
|  | HCP5B | 2.68145e-06 | 0.763 | Pancreas |
|  | HCP5B | 1.7837e-08 | 0.808 | Pituitary |
|  | HCP5B | 1.7261e-07 | 0.913 | Prostate |
|  | HCP5B | 3.45671e-07 | 0.515 | Skin Not Sun Exposed Suprapubic |
|  | HCP5B | 5.04979e-12 | 0.648 | Skin Sun Exposed Lower leg |
|  | HCP5B | 0.000100367 | 0.932 | Small Intestine Terminal Ileum |
|  | HCP5B | 2.04327e-05 | 0.712 | Spleen |
|  | HCP5B | 3.0375e-07 | 0.719 | Stomach |
|  | HCP5B | 1.31352e-08 | 0.546 | Thyroid |
|  | HCP5B | 5.30755e-18 | 0.775 | Whole Blood |
|  | HLA-A | 0.000112085 | -0.226 | Adipose Subcutaneous |
|  | HLA-A | 5.45276e-06 | 0.457 | Artery Aorta |
|  | HLA-A | 0.000391537 | 0.199 | Artery Tibial |
|  | HLA-A | 0.000123035 | -0.245 | Muscle Skeletal |
|  | HLA-A | 3.5882e-07 | -0.290 | Skin Not Sun Exposed Suprapubic |
|  | HLA-A | 3.84283e-12 | -0.398 | Skin Sun Exposed Lower leg |
|  | HLA-B | 7.07652e-07 | 0.514 | Testis |
|  | HLA-C | 2.04941e-06 | 0.539 | Artery Aorta |
|  | HLA-C | 1.76681e-07 | 0.313 | Artery Tibial |
|  | HLA-C | 9.57782e-05 | 0.307 | Breast Mammary Tissue |
|  | HLA-C | 2.17729e-08 | 0.566 | Cells Cultured fibroblasts |
|  | HLA-C | 0.000141231 | 0.228 | Colon Transverse |
|  | HLA-C | 2.27287e-06 | 0.522 | Esophagus Gastroesophageal Junction |
|  | HLA-C | 3.75928e-07 | 0.394 | Esophagus Mucosa |
|  | HLA-C | 2.84711e-07 | 0.484 | Esophagus Muscularis |
|  | HLA-C | 0.000198938 | 0.288 | Lung |
|  | HLA-C | 1.36213e-14 | 0.567 | Muscle Skeletal |
|  | HLA-C | 8.56529e-05 | 0.308 | Nerve Tibial |
|  | HLA-C | 1.97489e-07 | 0.735 | Pancreas |
|  | HLA-C | 4.22911e-05 | 0.302 | Skin Sun Exposed Lower leg |
|  | HLA-C | 2.65624e-06 | 0.422 | Stomach |
|  | HLA-C | 6.02191e-05 | 0.379 | Testis |
|  | HLA-C | 9.60319e-05 | 0.296 | Thyroid |
|  | HLA-C | 0.000172415 | 0.190 | Whole Blood |
|  | HLA-F-AS1 | 0.000391993 | 0.228 | Whole Blood |
|  | HLA-G | 4.25238e-07 | -0.520 | Adipose Subcutaneous |
|  | HLA-G | 9.98606e-06 | -0.502 | Adipose Visceral Omentum |
|  | HLA-G | 2.0096e-07 | -0.494 | Artery Tibial |
|  | HLA-G | 0.000121754 | -0.561 | Brain Nucleus accumbens basal ganglia |
|  | HLA-G | 0.000105932 | -0.506 | Breast Mammary Tissue |
|  | HLA-G | 1.0588e-09 | -0.665 | Cells Cultured fibroblasts |
|  | HLA-G | 8.57823e-05 | -0.536 | Esophagus Gastroesophageal Junction |
|  | HLA-G | 6.69649e-05 | -0.399 | Esophagus Mucosa |
|  | HLA-G | 1.86163e-06 | -0.581 | Esophagus Muscularis |
|  | HLA-G | 1.67337e-05 | -0.563 | Heart Atrial Appendage |
|  | HLA-G | 1.97565e-05 | -0.502 | Heart Left Ventricle |
|  | HLA-G | 1.08076e-08 | -0.523 | Lung |
|  | HLA-G | 1.81086e-06 | -0.429 | Muscle Skeletal |
|  | HLA-G | 4.21549e-06 | -0.532 | Nerve Tibial |
|  | HLA-G | 2.64576e-07 | -0.527 | Skin Not Sun Exposed Suprapubic |
|  | HLA-G | 1.12871e-08 | -0.516 | Skin Sun Exposed Lower leg |
|  | HLA-G | 0.000227074 | -0.430 | Testis |
|  | HLA-G | 3.07781e-06 | -0.433 | Thyroid |
|  | HLA-G | 7.8411e-07 | -0.427 | Whole Blood |
|  | HLA-J | 9.9107e-08 | -0.530 | Adipose Subcutaneous |
|  | HLA-J | 2.80971e-05 | -0.467 | Artery Aorta |
|  | HLA-J | 3.56817e-06 | -0.395 | Artery Tibial |
|  | HLA-J | 7.4744e-08 | -0.555 | Breast Mammary Tissue |
|  | HLA-J | 4.22406e-06 | -0.501 | Cells Cultured fibroblasts |
|  | HLA-J | 8.19959e-08 | -0.419 | Colon Transverse |
|  | HLA-J | 5.334e-05 | -0.381 | Esophagus Mucosa |
|  | HLA-J | 0.000105039 | -0.403 | Heart Atrial Appendage |
|  | HLA-J | 1.17448e-05 | -0.390 | Heart Left Ventricle |
|  | HLA-J | 8.48062e-07 | -0.415 | Lung |
|  | HLA-J | 4.49735e-07 | -0.423 | Muscle Skeletal |
|  | HLA-J | 7.80809e-06 | -0.451 | Skin Not Sun Exposed Suprapubic |
|  | HLA-J | 1.67107e-11 | -0.620 | Skin Sun Exposed Lower leg |
|  | HLA-J | 3.26348e-06 | -0.329 | Thyroid |
|  | HLA-J | 9.1242e-05 | -0.330 | Whole Blood |
|  | HLA-K | 4.06615e-05 | -0.449 | Adipose Visceral Omentum |
|  | HLA-K | 6.78426e-05 | -0.514 | Artery Aorta |
|  | HLA-K | 7.03428e-06 | -0.463 | Artery Tibial |
|  | HLA-K | 0.000145135 | -0.706 | Brain Cerebellar Hemisphere |
|  | HLA-K | 1.87617e-07 | -0.918 | Brain Cerebellum |
|  | HLA-K | 2.28475e-05 | -0.729 | Brain Cortex |
|  | HLA-K | 1.06843e-05 | -0.695 | Brain Nucleus accumbens basal ganglia |
|  | HLA-K | 4.13813e-05 | -0.790 | Brain Spinal cord cervical c-1 |
|  | HLA-K | 1.00203e-13 | -0.778 | Cells Cultured fibroblasts |
|  | HLA-K | 0.000155142 | -0.481 | Esophagus Gastroesophageal Junction |
|  | HLA-K | 2.52993e-08 | -0.618 | Esophagus Mucosa |
|  | HLA-K | 0.000197258 | -0.451 | Esophagus Muscularis |
|  | HLA-K | 9.13016e-08 | -0.530 | Lung |
|  | HLA-K | 3.05402e-09 | -0.489 | Muscle Skeletal |
|  | HLA-K | 2.66466e-06 | -0.552 | Nerve Tibial |
|  | HLA-K | 1.25133e-10 | -0.671 | Skin Not Sun Exposed Suprapubic |
|  | HLA-K | 5.66213e-13 | -0.695 | Skin Sun Exposed Lower leg |
|  | HLA-K | 6.28692e-08 | -0.537 | Thyroid |
|  | HLA-K | 1.37366e-05 | -0.385 | Whole Blood |
|  | HLA-L | 1.14312e-05 | -0.421 | Whole Blood |
|  | HLA-S | 5.8258e-05 | -0.399 | Artery Tibial |
|  | HLA-T | 0.000145003 | -0.441 | Lung |
|  | HLA-T | 6.77213e-05 | -0.385 | Whole Blood |
|  | HLA-U | 1.30652e-06 | 0.578 | Cells Cultured fibroblasts |
|  | HLA-U | 8.70929e-06 | 0.657 | Esophagus Gastroesophageal Junction |
|  | HLA-U | 4.97026e-05 | 0.534 | Esophagus Muscularis |
|  | HLA-U | 1.43392e-05 | 0.540 | Nerve Tibial |
|  | HLA-U | 3.58656e-05 | 0.638 | Stomach |
|  | HLA-V | 2.69355e-05 | -0.690 | Brain Cerebellum |
|  | HLA-V | 2.16159e-05 | -0.346 | Lung |
|  | HLA-V | 3.84854e-06 | 0.353 | Testis |
|  | HLA-W | 0.000300839 | -0.321 | Whole Blood |
|  | IER3 | 9.09672e-05 | 0.255 | Artery Aorta |
|  | IER3 | 8.16389e-08 | 0.331 | Esophagus Mucosa |
|  | IER3 | 0.000122678 | 0.226 | Whole Blood |
|  | IFITM4P | 0.000582089 | -0.294 | Skin Sun Exposed Lower leg |
|  | LINC00243 | 4.69318e-06 | 0.456 | Adipose Visceral Omentum |
|  | LINC00243 | 0.000252639 | 0.389 | Cells Cultured fibroblasts |
|  | LINC00243 | 8.14103e-06 | 0.457 | Esophagus Mucosa |
|  | LINC00243 | 1.97216e-06 | 0.447 | Lung |
|  | LINC00243 | 4.94499e-05 | 0.400 | Skin Sun Exposed Lower leg |
|  | LINC00243 | 3.20113e-13 | 0.453 | Whole Blood |
|  | MIR6891 | 7.41266e-08 | -0.550 | Adipose Visceral Omentum |
|  | MIR6891 | 1.06815e-05 | -0.407 | Artery Tibial |
|  | MIR6891 | 1.09514e-05 | -0.522 | Colon Transverse |
|  | MIR6891 | 1.85543e-05 | -0.429 | Esophagus Mucosa |
|  | MIR6891 | 5.12623e-08 | -0.480 | Lung |
|  | MIR6891 | 1.81044e-05 | -0.493 | Nerve Tibial |
|  | MIR6891 | 2.91919e-05 | -0.597 | Pituitary |
|  | MIR6891 | 6.4027e-06 | -0.418 | Skin Not Sun Exposed Suprapubic |
|  | MIR6891 | 5.46562e-07 | -0.467 | Skin Sun Exposed Lower leg |
|  | MIR6891 | 5.83041e-05 | -0.367 | Thyroid |
|  | NRM | 0.000292949 | -0.185 | Artery Tibial |
|  | OR2H2 | 2.56071e-05 | 0.462 | Heart Atrial Appendage |
|  | OR2H2 | 2.14982e-08 | 0.691 | Heart Left Ventricle |
|  | POU5F1 | 4.96399e-06 | 0.417 | Adipose Subcutaneous |
|  | POU5F1 | 7.04651e-05 | 0.415 | Adipose Visceral Omentum |
|  | POU5F1 | 1.44768e-05 | 0.469 | Artery Aorta |
|  | POU5F1 | 2.73298e-06 | 0.418 | Artery Tibial |
|  | POU5F1 | 2.82495e-05 | 0.357 | Thyroid |
|  | PPP1R18 | 3.94742e-07 | -0.208 | Adipose Visceral Omentum |
|  | PPP1R18 | 1.49414e-07 | -0.440 | Adrenal Gland |
|  | PPP1R18 | 5.30272e-22 | -0.515 | Artery Aorta |
|  | PPP1R18 | 2.59884e-05 | -0.344 | Artery Coronary |
|  | PPP1R18 | 4.62322e-15 | -0.323 | Artery Tibial |
|  | PPP1R18 | 7.24851e-06 | -0.203 | Breast Mammary Tissue |
|  | PPP1R18 | 5.07217e-12 | -0.250 | Cells Cultured fibroblasts |
|  | PPP1R18 | 3.81657e-06 | -0.312 | Esophagus Gastroesophageal Junction |
|  | PPP1R18 | 3.35633e-13 | -0.340 | Esophagus Muscularis |
|  | PPP1R18 | 3.1433e-07 | -0.189 | Lung |
|  | PPP1R18 | 8.7342e-08 | -0.241 | Nerve Tibial |
|  | PPP1R18 | 1.65812e-06 | -0.278 | Prostate |
|  | PPP1R18 | 5.56675e-07 | -0.193 | Thyroid |
|  | PSORS1C1 | 8.59878e-07 | -0.438 | Adipose Subcutaneous |
|  | PSORS1C1 | 7.58841e-08 | -0.465 | Adipose Visceral Omentum |
|  | PSORS1C1 | 8.1278e-06 | -0.518 | Artery Aorta |
|  | PSORS1C1 | 7.69171e-07 | -0.482 | Artery Tibial |
|  | PSORS1C1 | 3.14605e-06 | -0.714 | Brain Hypothalamus |
|  | PSORS1C1 | 4.01147e-05 | -0.373 | Breast Mammary Tissue |
|  | PSORS1C1 | 2.09536e-05 | -0.329 | Cells Cultured fibroblasts |
|  | PSORS1C1 | 8.31992e-06 | -0.574 | Colon Sigmoid |
|  | PSORS1C1 | 0.000178958 | -0.431 | Colon Transverse |
|  | PSORS1C1 | 2.49897e-05 | -0.519 | Esophagus Gastroesophageal Junction |
|  | PSORS1C1 | 1.47189e-05 | -0.482 | Esophagus Mucosa |
|  | PSORS1C1 | 7.34259e-06 | -0.438 | Esophagus Muscularis |
|  | PSORS1C1 | 4.70467e-12 | -0.820 | Heart Atrial Appendage |
|  | PSORS1C1 | 4.41894e-12 | -0.860 | Heart Left Ventricle |
|  | PSORS1C1 | 1.29782e-06 | -0.473 | Lung |
|  | PSORS1C1 | 6.42555e-08 | -0.604 | Nerve Tibial |
|  | PSORS1C1 | 3.74934e-05 | -0.471 | Stomach |
|  | PSORS1C1 | 1.60379e-16 | -0.805 | Thyroid |
|  | PSORS1C2 | 8.17352e-05 | -0.416 | Adipose Visceral Omentum |
|  | PSORS1C2 | 0.000100298 | -0.410 | Artery Tibial |
|  | PSORS1C2 | 7.24424e-07 | -0.527 | Thyroid |
|  | RNF39 | 3.10442e-05 | 0.534 | Pancreas |
|  | RNF39 | 0.000233871 | -0.319 | Thyroid |
|  | RPL23AP1 | 2.27964e-06 | 0.456 | Adipose Subcutaneous |
|  | RPL23AP1 | 7.29925e-05 | 0.340 | Adipose Visceral Omentum |
|  | RPL23AP1 | 0.000457088 | 0.359 | Artery Tibial |
|  | RPL23AP1 | 0.00019126 | -0.610 | Brain Cerebellum |
|  | RPL23AP1 | 0.000105603 | 0.515 | Esophagus Gastroesophageal Junction |
|  | RPL23AP1 | 5.04316e-05 | 0.329 | Lung |
|  | RPL23AP1 | 6.99796e-05 | 0.422 | Nerve Tibial |
|  | RPL23AP1 | 0.000100031 | 0.343 | Skin Sun Exposed Lower leg |
|  | RPL23AP1 | 7.03355e-05 | 0.486 | Spleen |
|  | RPL23AP1 | 8.74422e-07 | 0.422 | Whole Blood |
|  | SFTA2 | 6.24969e-06 | 0.175 | Lung |
|  | TRIM26 | 7.05074e-05 | 0.394 | Brain Cerebellar Hemisphere |
|  | TRIM26 | 8.41105e-06 | -0.207 | Testis |
|  | TRIM31 | 5.06256e-06 | -0.562 | Liver |
|  | TRIM31 | 2.14142e-07 | -0.421 | Lung |
|  | TRIM31 | 6.82386e-05 | -0.308 | Skin Not Sun Exposed Suprapubic |
|  | VARS2 | 2.52586e-06 | -0.360 | Adipose Subcutaneous |
|  | VARS2 | 1.81642e-06 | -0.314 | Artery Tibial |
|  | VARS2 | 7.45674e-07 | -0.525 | Brain Frontal Cortex BA9 |
|  | VARS2 | 2.33688e-05 | -0.382 | Colon Transverse |
|  | VARS2 | 0.000117008 | -0.423 | Heart Atrial Appendage |
|  | VARS2 | 4.04872e-05 | -0.409 | Heart Left Ventricle |
|  | VARS2 | 5.76125e-05 | -0.336 | Nerve Tibial |
|  | VARS2 | 0.000102586 | -0.525 | Pancreas |
|  | VARS2 | 1.15991e-05 | -0.338 | Skin Not Sun Exposed Suprapubic |
|  | VARS2 | 5.36709e-11 | -0.520 | Skin Sun Exposed Lower leg |
|  | VARS2 | 4.11353e-08 | -0.214 | Whole Blood |
|  | WASF5P | 1.61435e-07 | 0.873 | Pancreas |
|  | WASF5P | 1.92537e-06 | 0.731 | Pituitary |
|  | ZDHHC20P1 | 2.84422e-11 | 0.794 | Testis |
|  | ZFP57 | 4.96904e-15 | 0.798 | Adipose Subcutaneous |
|  | ZFP57 | 4.80959e-17 | 0.941 | Adipose Visceral Omentum |
|  | ZFP57 | 3.49029e-08 | 0.901 | Adrenal Gland |
|  | ZFP57 | 1.34262e-10 | 0.826 | Artery Aorta |
|  | ZFP57 | 1.64142e-07 | 0.974 | Artery Coronary |
|  | ZFP57 | 2.87299e-19 | 0.937 | Artery Tibial |
|  | ZFP57 | 6.24856e-08 | 0.673 | Breast Mammary Tissue |
|  | ZFP57 | 5.74106e-13 | 0.730 | Cells Cultured fibroblasts |
|  | ZFP57 | 3.33788e-07 | 0.781 | Colon Sigmoid |
|  | ZFP57 | 1.29166e-09 | 0.730 | Colon Transverse |
|  | ZFP57 | 2.16087e-08 | 0.813 | Esophagus Gastroesophageal Junction |
|  | ZFP57 | 2.68222e-14 | 0.833 | Esophagus Mucosa |
|  | ZFP57 | 3.43668e-11 | 0.843 | Esophagus Muscularis |
|  | ZFP57 | 1.58123e-09 | 0.816 | Heart Atrial Appendage |
|  | ZFP57 | 4.49442e-09 | 0.783 | Heart Left Ventricle |
|  | ZFP57 | 9.11674e-09 | 0.888 | Liver |
|  | ZFP57 | 1.18317e-11 | 0.759 | Lung |
|  | ZFP57 | 4.25677e-12 | 0.753 | Nerve Tibial |
|  | ZFP57 | 0.000119674 | 0.605 | Pancreas |
|  | ZFP57 | 1.76987e-06 | 0.715 | Pituitary |
|  | ZFP57 | 4.3441e-12 | 0.750 | Skin Not Sun Exposed Suprapubic |
|  | ZFP57 | 4.30178e-18 | 0.848 | Skin Sun Exposed Lower leg |
|  | ZFP57 | 8.60592e-05 | 0.973 | Small Intestine Terminal Ileum |
|  | ZFP57 | 1.66978e-08 | 0.998 | Spleen |
|  | ZFP57 | 1.24578e-09 | 0.854 | Stomach |
|  | ZFP57 | 1.82968e-16 | 0.821 | Thyroid |
|  | ZFP57 | 1.07782e-05 | 0.828 | Vagina |
|  | ZFP57 | 1.2604e-21 | 0.852 | Whole Blood |
|  | ZNRD1 | 0.000160373 | -0.217 | Adipose Subcutaneous |
|  | ZNRD1 | 1.0778e-05 | -0.290 | Esophagus Mucosa |
|  | ZNRD1 | 6.11977e-06 | -0.282 | Nerve Tibial |
|  | ZNRD1 | 1.65142e-06 | -0.340 | Thyroid |
| rs57440165 | BTN2A2 | 1.63509e-06 | -0.714133 | Pancreas |
|  | BTN2A2 | 2.02132e-09 | -0.36093 | Skin Sun Exposed Lower leg |
|  | BTN2A2 | 0.000143256 | 0.325227 | Testis |
|  | BTN3A2 | 1.81389e-48 | -1.04199 | Adipose Subcutaneous |
|  | BTN3A2 | 1.0013e-38 | -1.03655 | Adipose Visceral Omentum |
|  | BTN3A2 | 5.41425e-16 | -1.29168 | Adrenal Gland |
|  | BTN3A2 | 3.65707e-38 | -1.12442 | Artery Aorta |
|  | BTN3A2 | 1.49394e-19 | -0.991283 | Artery Coronary |
|  | BTN3A2 | 3.32934e-60 | -0.961344 | Artery Tibial |
|  | BTN3A2 | 7.93651e-08 | -0.862062 | Brain Amygdala |
|  | BTN3A2 | 9.21787e-11 | -1.11945 | Brain Anterior cingulate cortex BA24 |
|  | BTN3A2 | 1.10293e-12 | -1.00853 | Brain Caudate basal ganglia |
|  | BTN3A2 | 5.01909e-09 | -1.00579 | Brain Cerebellar Hemisphere |
|  | BTN3A2 | 9.75452e-12 | -1.05101 | Brain Cerebellum |
|  | BTN3A2 | 1.03985e-13 | -0.973505 | Brain Cortex |
|  | BTN3A2 | 6.67791e-11 | -0.929489 | Brain Frontal Cortex BA9 |
|  | BTN3A2 | 8.08471e-10 | -0.99574 | Brain Hippocampus |
|  | BTN3A2 | 7.4309e-10 | -1.02145 | Brain Hypothalamus |
|  | BTN3A2 | 5.01779e-10 | -0.902199 | Brain Nucleus accumbens basal ganglia |
|  | BTN3A2 | 5.5421e-09 | -0.987789 | Brain Putamen basal ganglia |
|  | BTN3A2 | 4.17197e-12 | -1.19758 | Brain Spinal cord cervical c-1 |
|  | BTN3A2 | 8.2313e-39 | -1.18076 | Breast Mammary Tissue |
|  | BTN3A2 | 1.50592e-43 | -1.232 | Cells Cultured fibroblasts |
|  | BTN3A2 | 1.04107e-10 | -1.11803 | Cells EBV-transformed lymphocytes |
|  | BTN3A2 | 1.33333e-18 | -0.951182 | Colon Sigmoid |
|  | BTN3A2 | 1.43691e-36 | -0.97496 | Colon Transverse |
|  | BTN3A2 | 5.56889e-37 | -1.16121 | Esophagus Gastroesophageal Junction |
|  | BTN3A2 | 2.8426e-64 | -1.23575 | Esophagus Mucosa |
|  | BTN3A2 | 5.75778e-54 | -1.19999 | Esophagus Muscularis |
|  | BTN3A2 | 2.5939e-39 | -1.26476 | Heart Atrial Appendage |
|  | BTN3A2 | 4.16947e-46 | -1.14197 | Heart Left Ventricle |
|  | BTN3A2 | 6.5545e-17 | -1.18581 | Liver |
|  | BTN3A2 | 6.57737e-59 | -1.20031 | Lung |
|  | BTN3A2 | 2.02097e-09 | -1.19824 | Minor Salivary Gland |
|  | BTN3A2 | 4.75397e-54 | -1.18601 | Muscle Skeletal |
|  | BTN3A2 | 9.42452e-51 | -1.09158 | Nerve Tibial |
|  | BTN3A2 | 3.12225e-12 | -1.07482 | Ovary |
|  | BTN3A2 | 2.14639e-23 | -1.24413 | Pancreas |
|  | BTN3A2 | 4.04035e-15 | -1.14647 | Pituitary |
|  | BTN3A2 | 4.23424e-14 | -0.966953 | Prostate |
|  | BTN3A2 | 6.82526e-63 | -1.35737 | Skin Sun Exposed Lower leg |
|  | BTN3A2 | 9.26899e-07 | -0.656779 | Small Intestine Terminal Ileum |
|  | BTN3A2 | 1.19796e-26 | -1.30391 | Spleen |
|  | BTN3A2 | 1.44366e-38 | -1.12289 | Stomach |
|  | BTN3A2 | 1.02269e-25 | -1.21418 | Testis |
|  | BTN3A2 | 1.16029e-57 | -1.11581 | Thyroid |
|  | BTN3A2 | 1.38304e-13 | -1.17712 | Uterus |
|  | BTN3A2 | 7.52727e-19 | -1.15467 | Vagina |
|  | BTN3A2 | 7.32293e-76 | -1.01286 | Whole Blood |
|  | GUSBP2 | 0.000734044 | 0.357344 | Cells Cultured fibroblasts |
|  | GUSBP2 | 0.000623773 | 0.356808 | Esophagus Mucosa |
|  | HIST1H2BK | 0.000211362 | 0.302462 | Adipose Subcutaneous |
|  | HIST1H2BK | 0.000622081 | 0.230766 | Artery Tibial |
|  | HIST1H2BN | 0.000440413 | -0.332079 | Artery Aorta |
|  | HIST1H3H | 8.77278e-05 | -0.431156 | Esophagus Muscularis |
|  | HMGN4 | 0.000234774 | -0.183949 | Muscle Skeletal |
|  | LINC00240 | 0.000108808 | 0.411439 | Adipose Subcutaneous |
|  | LINC00240 | 0.00033378 | 0.424756 | Adipose Visceral Omentum |
|  | LINC00240 | 0.000422218 | 0.345334 | Nerve Tibial |
|  | LINC00240 | 1.48968e-05 | 0.416329 | Skin Sun Exposed Lower leg |
|  | MCFD2P1 | 0.000324526 | 0.492575 | Testis |
|  | PRSS16 | 0.000134977 | 0.409701 | Adipose Subcutaneous |
|  | PRSS16 | 7.73565e-07 | 0.716607 | Adrenal Gland |
|  | PRSS16 | 5.72144e-06 | 0.493672 | Artery Aorta |
|  | PRSS16 | 1.52422e-09 | 0.623674 | Artery Tibial |
|  | PRSS16 | 3.28229e-09 | 0.971681 | Brain Cerebellar Hemisphere |
|  | PRSS16 | 5.08444e-10 | 0.920263 | Brain Cerebellum |
|  | PRSS16 | 0.000345619 | 0.230346 | Esophagus Mucosa |
|  | PRSS16 | 1.94595e-06 | 0.544988 | Esophagus Muscularis |
|  | PRSS16 | 8.96341e-05 | 0.506318 | Ovary |
|  | PRSS16 | 1.55339e-06 | 0.410011 | Prostate |
|  | PRSS16 | 7.50445e-08 | 0.306238 | Skin Sun Exposed Lower leg |
|  | PRSS16 | 6.20834e-07 | 0.681471 | Spleen |
|  | PRSS16 | 3.66729e-08 | 0.249633 | Thyroid |
|  | TRIM38 | 1.49289e-05 | -0.196827 | Whole Blood |
|  | U91328.19 | 3.43947e-05 | 0.272733 | Whole Blood |
|  | ZNF204P | 0.000194031 | 0.221219 | Artery Aorta |
|  | ZNF204P | 0.00063911 | 0.150212 | Thyroid |
|  | ZNF391 | 2.59498e-05 | 0.331677 | Adipose Subcutaneous |
|  | ZNF391 | 5.82283e-06 | 0.483418 | Artery Aorta |
|  | ZNF391 | 1.07668e-05 | 0.398479 | Artery Tibial |
|  | ZNF391 | 2.70235e-07 | 0.46812 | Cells Cultured fibroblasts |
|  | ZNF391 | 2.23505e-05 | 0.52299 | Colon Sigmoid |
|  | ZNF391 | 0.00012123 | 0.377297 | Heart Atrial Appendage |
|  | ZNF391 | 4.13308e-05 | 0.592011 | Spleen |
|  | ZNF391 | 6.27073e-05 | 0.274514 | Thyroid |

**Table S13. Significant SNP-gene pairs (FDR < 0.05) associated with the shared SNP for bipolar disease and PTB in 44 human tissues obtained from GTEx Portal**

| **Index SNP** | **Gene** | **P-value** | **Effect size** | **Tissue** |
| --- | --- | --- | --- | --- |
| rs60476972 | C16orf72 | 1.74519e-05 | -0.095 | Artery Tibial |
| rs7813444 | RP11-1D12.2 | 1.60168E-04 | -0.106 | Whole Blood |
| rs3132948 | BAG6 | 1.24192E-04 | 0.123 | Artery Tibial |
|  | BTNL2 | 1.20211E-04 | 0.202 | Nerve Tibial |
|  | CSNK2B | 6.66389e-05 | -0.133 | Colon Sigmoid |
|  | CYP21A1P | 1.39805e-05 | 0.227 | Adipose Subcutaneous |
|  | CYP21A1P | 8.15302e-06 | 0.182 | Thyroid |
|  | CYP21A2 | 1.08074E-04 | 0.190 | Heart Atrial Appendage |
|  | CYP21A2 | 2.73625E-04 | 0.140 | Thyroid |
|  | GPANK1 | 1.71167e-07 | 0.148 | Adipose Subcutaneous |
|  | GPANK1 | 2.68e-05 | 0.101 | Skin Sun Exposed Lower leg |
|  | GPANK1 | 3.76702E-04 | 0.113 | Thyroid |
|  | HLA-C | 5.01016e-07 | -0.163 | Artery Tibial |
|  | HLA-C | 3.82021e-05 | -0.379 | Brain Cerebellar Hemisphere |
|  | HLA-C | 5.73976e-08 | -0.315 | Cells Cultured fibroblasts |
|  | HLA-C | 6.29684e-05 | -0.244 | Esophagus Gastroesophageal Junction |
|  | HLA-C | 1.60989E-04 | -0.168 | Esophagus Mucosa |
|  | HLA-C | 1.37361e-05 | -0.226 | Esophagus Muscularis |
|  | HLA-C | 1.01183E-04 | -0.185 | Heart Left Ventricle |
|  | HLA-C | 3.40118e-06 | -0.202 | Muscle Skeletal |
|  | HLA-C | 2.30446e-06 | -0.331 | Pancreas |
|  | HLA-C | 4.36723e-05 | -0.185 | Skin Not Sun Exposed Suprapubic |
|  | HLA-C | 1.04554e-08 | -0.240 | Skin Sun Exposed Lower leg |
|  | HLA-C | 1.10708E-04 | -0.193 | Stomach |
|  | HLA-C | 2.70088e-05 | -0.177 | Thyroid |
|  | HLA-DRB1 | 4.06166e-05 | -0.191 | Esophagus Gastroesophageal Junction |
|  | HLA-DRB1 | 1.00379e-05 | -0.250 | Heart Atrial Appendage |
|  | HLA-DRB5 | 6.19696e-06 | -0.236 | Adipose Subcutaneous |
|  | HLA-DRB5 | 6.38731e-05 | -0.248 | Adipose Visceral Omentum |
|  | HLA-DRB5 | 5.62531e-05 | -0.393 | Adrenal Gland |
|  | HLA-DRB5 | 1.3713e-07 | -0.257 | Artery Tibial |
|  | HLA-DRB5 | 3.87216e-07 | -0.305 | Breast Mammary Tissue |
|  | HLA-DRB5 | 6.27002e-06 | -0.352 | Colon Sigmoid |
|  | HLA-DRB5 | 2.35204e-05 | -0.282 | Colon Transverse |
|  | HLA-DRB5 | 5.95697e-06 | -0.248 | Esophagus Mucosa |
|  | HLA-DRB5 | 6.60232e-05 | -0.268 | Esophagus Muscularis |
|  | HLA-DRB5 | 4.12249e-07 | -0.375 | Heart Atrial Appendage |
|  | HLA-DRB5 | 1.71684e-05 | -0.308 | Heart Left Ventricle |
|  | HLA-DRB5 | 1.17797E-04 | -0.207 | Lung |
|  | HLA-DRB5 | 2.16295e-06 | -0.234 | Muscle Skeletal |
|  | HLA-DRB5 | 4.18194e-08 | -0.312 | Nerve Tibial |
|  | HLA-DRB5 | 5.3885e-05 | -0.222 | Skin Not Sun Exposed Suprapubic |
|  | HLA-DRB5 | 2.53679e-08 | -0.307 | Skin Sun Exposed Lower leg |
|  | HLA-DRB5 | 9.3867e-07 | -0.267 | Thyroid |
|  | HLA-DRB5 | 3.89254e-05 | -0.147 | Whole Blood |
|  | HLA-DRB9 | 1.24200E-04 | 0.213 | Adipose Subcutaneous |
|  | HLA-DRB9 | 1.64766E-04 | 0.164 | Whole Blood |
|  | HLA-S | 5.05411e-06 | 0.262 | Adipose Subcutaneous |
|  | HLA-S | 6.41656e-05 | 0.261 | Adipose Visceral Omentum |
|  | HLA-S | 1.23858E-04 | 0.275 | Artery Aorta |
|  | HLA-S | 8.2424e-05 | 0.275 | Breast Mammary Tissue |
|  | HLA-S | 4.06126e-06 | 0.284 | Lung |
|  | HLA-S | 5.72727e-06 | 0.278 | Skin Not Sun Exposed Suprapubic |
|  | HLA-S | 1.27437E-04 | 0.275 | Stomach |
|  | HLA-S | 4.28519e-05 | 0.223 | Thyroid |
|  | HLA-S | 1.14033e-08 | 0.303 | Whole Blood |
|  | MICA | 2.29405E-04 | -0.130 | Lung |
|  | MICA | 4.5379e-06 | -0.185 | Thyroid |
|  | MIR6891 | 9.67516e-05 | 0.203 | Adipose Subcutaneous |
|  | MIR6891 | 1.63366E-04 | 0.189 | Artery Tibial |
|  | MIR6891 | 1.62865E-04 | 0.187 | Lung |
|  | MIR6891 | 2.28565e-05 | 0.470 | Uterus |
|  | NOTCH4 | 1.80225e-07 | -0.126 | Adipose Subcutaneous |
|  | NOTCH4 | 4.10655e-05 | -0.102 | Artery Tibial |
|  | NOTCH4 | 1.78677e-06 | -0.157 | Colon Transverse |
|  | NOTCH4 | 6.90377e-06 | -0.169 | Esophagus Mucosa |
|  | NOTCH4 | 1.88229e-08 | -0.203 | Esophagus Muscularis |
|  | NOTCH4 | 2.76258e-06 | -0.158 | Heart Atrial Appendage |
|  | NOTCH4 | 1.5306e-05 | -0.123 | Heart Left Ventricle |
|  | NOTCH4 | 2.57576e-05 | -0.135 | Muscle Skeletal |
|  | NOTCH4 | 7.57922e-08 | -0.190 | Nerve Tibial |
|  | NOTCH4 | 2.16807E-04 | -0.092 | Skin Sun Exposed Lower leg |
|  | NOTCH4 | 4.87694e-05 | -0.193 | Stomach |
|  | NOTCH4 | 4.62407e-07 | -0.161 | Thyroid |
|  | PRRT1 | 1.4007e-05 | 0.138 | Breast Mammary Tissue |
|  | PRRT1 | 1.04491E-04 | 0.111 | Esophagus Mucosa |
|  | PRRT1 | 3.41307e-07 | 0.188 | Skin Not Sun Exposed Suprapubic |
|  | PRRT1 | 4.21332e-07 | 0.181 | Skin Sun Exposed Lower leg |
|  | SKIV2L | 9.68542e-05 | 0.138 | Adipose Subcutaneous |
|  | SKIV2L | 1.45088e-05 | 0.112 | Cells Cultured fibroblasts |
|  | SKIV2L | 3.33386e-05 | 0.208 | Stomach |
|  | WASF5P | 2.46851e-05 | -0.374 | Brain Cerebellar Hemisphere |
|  | ZBTB12 | 2.84956E-04 | 0.107 | Adipose Subcutaneous |
| rs9273363 | AGER | 4.82694e-05 | 0.159 | Nerve Tibial |
|  | C4A | 5.00034e-08 | -0.267 | Adipose Subcutaneous |
|  | C4A | 2.42258e-06 | -0.186 | Adipose Visceral Omentum |
|  | C4A | 4.17136e-08 | -0.283 | Artery Aorta |
|  | C4A | 7.22554e-07 | -0.205 | Artery Tibial |
|  | C4A | 3.70173e-06 | -0.340 | Brain Caudate basal ganglia |
|  | C4A | 6.693e-07 | -0.525 | Brain Cerebellar Hemisphere |
|  | C4A | 9.89045e-09 | -0.557 | Brain Cerebellum |
|  | C4A | 2.8654e-05 | -0.196 | Breast Mammary Tissue |
|  | C4A | 2.10493e-10 | -0.233 | Cells Cultured fibroblasts |
|  | C4A | 6.80891e-06 | -0.245 | Colon Sigmoid |
|  | C4A | 7.13686e-06 | -0.236 | Colon Transverse |
|  | C4A | 6.04697e-06 | -0.249 | Esophagus Gastroesophageal Junction |
|  | C4A | 5.36268e-07 | -0.245 | Esophagus Mucosa |
|  | C4A | 1.25546e-06 | -0.182 | Esophagus Muscularis |
|  | C4A | 5.29902e-07 | -0.250 | Heart Atrial Appendage |
|  | C4A | 2.42066e-05 | -0.194 | Heart Left Ventricle |
|  | C4A | 1.15149e-06 | -0.284 | Liver |
|  | C4A | 2.07895e-08 | -0.247 | Lung |
|  | C4A | 1.94018e-07 | -0.221 | Muscle Skeletal |
|  | C4A | 8.35119e-14 | -0.366 | Nerve Tibial |
|  | C4A | 1.95918e-05 | -0.213 | Pancreas |
|  | C4A | 2.46296e-05 | -0.266 | Prostate |
|  | C4A | 1.45807e-07 | -0.315 | Skin Not Sun Exposed Suprapubic |
|  | C4A | 5.00306e-11 | -0.327 | Skin Sun Exposed Lower leg |
|  | C4A | 1.19928e-05 | -0.239 | Stomach |
|  | C4A | 4.32276e-07 | -0.361 | Testis |
|  | C4A | 1.0932e-11 | -0.292 | Thyroid |
|  | C4A | 4.39835e-14 | -0.384 | Whole Blood |
|  | C4B | 9.37599e-10 | 0.219 | Cells Cultured fibroblasts |
|  | C4B | 7.54937e-10 | 0.356 | Skin Not Sun Exposed Suprapubic |
|  | C4B | 2.33909e-07 | 0.261 | Skin Sun Exposed Lower leg |
|  | C4B | 2.14956e-08 | 0.313 | Whole Blood |
|  | CYP21A1P | 1.91981e-05 | -0.247 | Adipose Subcutaneous |
|  | CYP21A1P | 1.29001e-05 | -0.497 | Brain Cerebellum |
|  | CYP21A1P | 4.92431e-05 | -0.314 | Liver |
|  | CYP21A1P | 1.07054E-04 | -0.220 | Lung |
|  | CYP21A1P | 1.06429e-06 | -0.296 | Nerve Tibial |
|  | CYP21A1P | 2.69712e-06 | -0.308 | Skin Not Sun Exposed Suprapubic |
|  | CYP21A1P | 3.2497e-05 | -0.230 | Skin Sun Exposed Lower leg |
|  | CYP21A1P | 3.85106e-05 | -0.187 | Thyroid |
|  | CYP21A2 | 5.16937e-11 | 0.379 | Adipose Subcutaneous |
|  | CYP21A2 | 4.78417e-05 | 0.208 | Artery Tibial |
|  | CYP21A2 | 5.74106e-07 | 0.314 | Skin Not Sun Exposed Suprapubic |
|  | CYP21A2 | 2.30571e-07 | 0.294 | Skin Sun Exposed Lower leg |
|  | CYP21A2 | 5.22135e-07 | 0.278 | Whole Blood |
|  | HCG23 | 5.89647e-05 | 0.488 | Brain Caudate basal ganglia |
|  | HLA-DMA | 4.51871e-06 | 0.220 | Cells Cultured fibroblasts |
|  | HLA-DMA | 3.1615e-05 | 0.150 | Esophagus Muscularis |
|  | HLA-DMA | 4.89496e-06 | 0.170 | Muscle Skeletal |
|  | HLA-DMA | 2.23847e-07 | 0.221 | Thyroid |
|  | HLA-DOB | 2.03282e-05 | 0.225 | Adipose Subcutaneous |
|  | HLA-DOB | 5.54417e-05 | 0.386 | Adrenal Gland |
|  | HLA-DOB | 2.61264e-05 | 0.280 | Artery Aorta |
|  | HLA-DOB | 1.20651E-04 | 0.203 | Artery Tibial |
|  | HLA-DOB | 7.4601e-05 | 0.297 | Colon Sigmoid |
|  | HLA-DOB | 5.4696e-06 | 0.246 | Esophagus Mucosa |
|  | HLA-DOB | 9.73407e-06 | 0.288 | Esophagus Muscularis |
|  | HLA-DOB | 3.68929e-06 | 0.246 | Lung |
|  | HLA-DOB | 1.82813e-07 | 0.278 | Muscle Skeletal |
|  | HLA-DOB | 1.0147e-05 | 0.253 | Skin Sun Exposed Lower leg |
|  | HLA-DQA1 | 2.2876e-09 | -0.261 | Adipose Subcutaneous |
|  | HLA-DQA1 | 9.30983e-10 | -0.307 | Adipose Visceral Omentum |
|  | HLA-DQA1 | 2.50765e-08 | -0.259 | Artery Aorta |
|  | HLA-DQA1 | 1.39654e-14 | -0.259 | Artery Tibial |
|  | HLA-DQA1 | 1.03758E-04 | -0.206 | Breast Mammary Tissue |
|  | HLA-DQA1 | 2.15849e-09 | -0.643 | Cells EBV-transformed lymphocytes |
|  | HLA-DQA1 | 2.27272e-07 | -0.325 | Colon Sigmoid |
|  | HLA-DQA1 | 1.59078e-11 | -0.285 | Colon Transverse |
|  | HLA-DQA1 | 9.75278e-11 | -0.445 | Esophagus Gastroesophageal Junction |
|  | HLA-DQA1 | 4.15879e-15 | -0.319 | Esophagus Mucosa |
|  | HLA-DQA1 | 1.08576e-11 | -0.390 | Esophagus Muscularis |
|  | HLA-DQA1 | 5.49978e-09 | -0.377 | Heart Atrial Appendage |
|  | HLA-DQA1 | 2.9647e-07 | -0.303 | Heart Left Ventricle |
|  | HLA-DQA1 | 4.62654e-06 | -0.287 | Liver |
|  | HLA-DQA1 | 3.17661e-23 | -0.410 | Lung |
|  | HLA-DQA1 | 4.26742e-10 | -0.262 | Muscle Skeletal |
|  | HLA-DQA1 | 4.48019e-13 | -0.312 | Nerve Tibial |
|  | HLA-DQA1 | 3.95316e-10 | -0.378 | Pancreas |
|  | HLA-DQA1 | 8.09638e-06 | -0.288 | Pituitary |
|  | HLA-DQA1 | 5.33179e-21 | -0.404 | Skin Not Sun Exposed Suprapubic |
|  | HLA-DQA1 | 1.33683e-19 | -0.394 | Skin Sun Exposed Lower leg |
|  | HLA-DQA1 | 3.61926e-08 | -0.300 | Spleen |
|  | HLA-DQA1 | 1.0725e-10 | -0.310 | Stomach |
|  | HLA-DQA1 | 3.12007e-07 | -0.323 | Testis |
|  | HLA-DQA1 | 1.96434e-16 | -0.327 | Thyroid |
|  | HLA-DQA1 | 4.61538e-23 | -0.232 | Whole Blood |
|  | HLA-DQA2 | 3.0907e-16 | 0.542 | Adipose Subcutaneous |
|  | HLA-DQA2 | 2.29706e-21 | 0.658 | Adipose Visceral Omentum |
|  | HLA-DQA2 | 2.18346e-10 | 0.635 | Adrenal Gland |
|  | HLA-DQA2 | 3.06459e-16 | 0.579 | Artery Aorta |
|  | HLA-DQA2 | 2.29653e-09 | 0.623 | Artery Coronary |
|  | HLA-DQA2 | 6.40722e-19 | 0.512 | Artery Tibial |
|  | HLA-DQA2 | 7.65726e-06 | 0.584 | Brain Amygdala |
|  | HLA-DQA2 | 2.10738e-05 | 0.441 | Brain Cerebellum |
|  | HLA-DQA2 | 3.41375e-07 | 0.561 | Brain Hippocampus |
|  | HLA-DQA2 | 3.5233e-06 | 0.479 | Brain Nucleus accumbens basal ganglia |
|  | HLA-DQA2 | 5.64089e-16 | 0.649 | Breast Mammary Tissue |
|  | HLA-DQA2 | 1.50687e-06 | 0.575 | Cells EBV-transformed lymphocytes |
|  | HLA-DQA2 | 1.45281e-10 | 0.556 | Colon Sigmoid |
|  | HLA-DQA2 | 1.08682e-11 | 0.508 | Colon Transverse |
|  | HLA-DQA2 | 6.98231e-11 | 0.543 | Esophagus Gastroesophageal Junction |
|  | HLA-DQA2 | 1.84249e-14 | 0.460 | Esophagus Mucosa |
|  | HLA-DQA2 | 7.94611e-18 | 0.607 | Esophagus Muscularis |
|  | HLA-DQA2 | 8.14435e-13 | 0.566 | Heart Atrial Appendage |
|  | HLA-DQA2 | 7.28469e-13 | 0.560 | Heart Left Ventricle |
|  | HLA-DQA2 | 1.08848e-05 | 0.458 | Liver |
|  | HLA-DQA2 | 1.5524e-20 | 0.592 | Lung |
|  | HLA-DQA2 | 4.87092e-20 | 0.525 | Muscle Skeletal |
|  | HLA-DQA2 | 8.65838e-17 | 0.559 | Nerve Tibial |
|  | HLA-DQA2 | 2.35077e-08 | 0.616 | Ovary |
|  | HLA-DQA2 | 1.46954e-10 | 0.513 | Pancreas |
|  | HLA-DQA2 | 2.70047e-09 | 0.567 | Pituitary |
|  | HLA-DQA2 | 2.70952e-09 | 0.614 | Prostate |
|  | HLA-DQA2 | 7.1833e-12 | 0.357 | Skin Not Sun Exposed Suprapubic |
|  | HLA-DQA2 | 7.94613e-20 | 0.481 | Skin Sun Exposed Lower leg |
|  | HLA-DQA2 | 6.29524e-08 | 0.499 | Spleen |
|  | HLA-DQA2 | 3.25671e-16 | 0.567 | Stomach |
|  | HLA-DQA2 | 4.20076e-08 | 0.485 | Testis |
|  | HLA-DQA2 | 1.04348e-16 | 0.504 | Thyroid |
|  | HLA-DQA2 | 9.09783e-07 | 0.612 | Uterus |
|  | HLA-DQA2 | 2.39475e-06 | 0.566 | Vagina |
|  | HLA-DQA2 | 7.40477e-23 | 0.523 | Whole Blood |
|  | HLA-DQB1 | 5.64201e-30 | -0.596 | Adipose Subcutaneous |
|  | HLA-DQB1 | 1.23963e-29 | -0.628 | Adipose Visceral Omentum |
|  | HLA-DQB1 | 7.99454e-19 | -0.479 | Artery Aorta |
|  | HLA-DQB1 | 7.4646e-12 | -0.541 | Artery Coronary |
|  | HLA-DQB1 | 2.00289e-33 | -0.498 | Artery Tibial |
|  | HLA-DQB1 | 1.6422e-06 | -0.505 | Brain Amygdala |
|  | HLA-DQB1 | 2.88391e-15 | -0.870 | Brain Anterior cingulate cortex BA24 |
|  | HLA-DQB1 | 6.86395e-11 | -0.597 | Brain Caudate basal ganglia |
|  | HLA-DQB1 | 3.44423e-09 | -0.551 | Brain Cerebellum |
|  | HLA-DQB1 | 2.08202e-18 | -0.805 | Brain Cortex |
|  | HLA-DQB1 | 6.35246e-15 | -0.765 | Brain Frontal Cortex BA9 |
|  | HLA-DQB1 | 7.62189e-07 | -0.461 | Brain Hippocampus |
|  | HLA-DQB1 | 5.54662e-08 | -0.549 | Brain Hypothalamus |
|  | HLA-DQB1 | 2.02716e-08 | -0.529 | Brain Nucleus accumbens basal ganglia |
|  | HLA-DQB1 | 3.06288e-08 | -0.636 | Brain Putamen basal ganglia |
|  | HLA-DQB1 | 1.88105e-05 | -0.461 | Brain Spinal cord cervical c-1 |
|  | HLA-DQB1 | 4.16608e-21 | -0.554 | Breast Mammary Tissue |
|  | HLA-DQB1 | 6.88352e-16 | -0.800 | Cells EBV-transformed lymphocytes |
|  | HLA-DQB1 | 6.65888e-16 | -0.595 | Colon Sigmoid |
|  | HLA-DQB1 | 9.47351e-20 | -0.528 | Colon Transverse |
|  | HLA-DQB1 | 7.50133e-23 | -0.680 | Esophagus Gastroesophageal Junction |
|  | HLA-DQB1 | 4.58116e-28 | -0.572 | Esophagus Mucosa |
|  | HLA-DQB1 | 7.91635e-23 | -0.617 | Esophagus Muscularis |
|  | HLA-DQB1 | 3.53277e-18 | -0.657 | Heart Atrial Appendage |
|  | HLA-DQB1 | 2.50437e-20 | -0.598 | Heart Left Ventricle |
|  | HLA-DQB1 | 1.20681e-13 | -0.559 | Liver |
|  | HLA-DQB1 | 4.55346e-29 | -0.507 | Lung |
|  | HLA-DQB1 | 7.09351e-07 | -0.613 | Minor Salivary Gland |
|  | HLA-DQB1 | 7.89075e-34 | -0.581 | Muscle Skeletal |
|  | HLA-DQB1 | 6.71639e-31 | -0.613 | Nerve Tibial |
|  | HLA-DQB1 | 7.36457e-07 | -0.364 | Ovary |
|  | HLA-DQB1 | 6.13002e-16 | -0.602 | Pancreas |
|  | HLA-DQB1 | 2.04453e-18 | -0.761 | Pituitary |
|  | HLA-DQB1 | 1.97153e-10 | -0.529 | Prostate |
|  | HLA-DQB1 | 1.21462e-40 | -0.674 | Skin Not Sun Exposed Suprapubic |
|  | HLA-DQB1 | 1.09622e-43 | -0.713 | Skin Sun Exposed Lower leg |
|  | HLA-DQB1 | 2.53609e-09 | -0.455 | Small Intestine Terminal Ileum |
|  | HLA-DQB1 | 8.39593e-15 | -0.589 | Spleen |
|  | HLA-DQB1 | 2.43858e-22 | -0.628 | Stomach |
|  | HLA-DQB1 | 3.2302e-15 | -0.654 | Testis |
|  | HLA-DQB1 | 5.80226e-40 | -0.654 | Thyroid |
|  | HLA-DQB1 | 5.44198e-07 | -0.568 | Uterus |
|  | HLA-DQB1 | 5.35315e-09 | -0.647 | Vagina |
|  | HLA-DQB1 | 4.16047e-38 | -0.462 | Whole Blood |
|  | HLA-DQB1-AS1 | 4.95962e-08 | -0.276 | Adipose Subcutaneous |
|  | HLA-DQB1-AS1 | 2.71215e-09 | -0.296 | Adipose Visceral Omentum |
|  | HLA-DQB1-AS1 | 3.01892e-06 | -0.239 | Artery Aorta |
|  | HLA-DQB1-AS1 | 1.18240E-04 | -0.148 | Artery Tibial |
|  | HLA-DQB1-AS1 | 1.22452e-06 | -0.610 | Brain Anterior cingulate cortex BA24 |
|  | HLA-DQB1-AS1 | 7.27317e-08 | -0.602 | Brain Cortex |
|  | HLA-DQB1-AS1 | 8.81677e-08 | -0.554 | Brain Frontal Cortex BA9 |
|  | HLA-DQB1-AS1 | 2.33079e-05 | -0.440 | Brain Hippocampus |
|  | HLA-DQB1-AS1 | 2.22156e-05 | -0.452 | Brain Hypothalamus |
|  | HLA-DQB1-AS1 | 1.4671e-05 | -0.253 | Breast Mammary Tissue |
|  | HLA-DQB1-AS1 | 1.55236e-05 | -0.239 | Colon Transverse |
|  | HLA-DQB1-AS1 | 5.60743e-05 | -0.217 | Esophagus Gastroesophageal Junction |
|  | HLA-DQB1-AS1 | 6.21256e-09 | -0.274 | Esophagus Mucosa |
|  | HLA-DQB1-AS1 | 1.70458e-05 | -0.256 | Heart Atrial Appendage |
|  | HLA-DQB1-AS1 | 3.26779e-08 | -0.299 | Heart Left Ventricle |
|  | HLA-DQB1-AS1 | 3.19227e-08 | -0.483 | Liver |
|  | HLA-DQB1-AS1 | 1.03028e-09 | -0.251 | Lung |
|  | HLA-DQB1-AS1 | 6.81903e-17 | -0.431 | Muscle Skeletal |
|  | HLA-DQB1-AS1 | 1.22028e-09 | -0.280 | Nerve Tibial |
|  | HLA-DQB1-AS1 | 4.94464e-16 | -0.650 | Pituitary |
|  | HLA-DQB1-AS1 | 1.25945e-05 | -0.316 | Prostate |
|  | HLA-DQB1-AS1 | 1.16927e-06 | -0.212 | Skin Not Sun Exposed Suprapubic |
|  | HLA-DQB1-AS1 | 2.76055e-07 | -0.241 | Skin Sun Exposed Lower leg |
|  | HLA-DQB1-AS1 | 5.77634e-05 | -0.307 | Spleen |
|  | HLA-DQB1-AS1 | 2.31534e-08 | -0.362 | Stomach |
|  | HLA-DQB1-AS1 | 6.28078e-09 | -0.421 | Testis |
|  | HLA-DQB1-AS1 | 3.1782e-18 | -0.367 | Thyroid |
|  | HLA-DQB1-AS1 | 2.56335e-05 | -0.391 | Vagina |
|  | HLA-DQB1-AS1 | 4.75154e-13 | -0.233 | Whole Blood |
|  | HLA-DQB2 | 2.91076e-13 | 0.463 | Adipose Subcutaneous |
|  | HLA-DQB2 | 3.60935e-17 | 0.544 | Adipose Visceral Omentum |
|  | HLA-DQB2 | 6.48573e-10 | 0.614 | Adrenal Gland |
|  | HLA-DQB2 | 1.82306e-09 | 0.402 | Artery Aorta |
|  | HLA-DQB2 | 3.52216e-05 | 0.396 | Artery Coronary |
|  | HLA-DQB2 | 1.39443e-12 | 0.360 | Artery Tibial |
|  | HLA-DQB2 | 2.32592e-10 | 0.495 | Breast Mammary Tissue |
|  | HLA-DQB2 | 3.87921e-06 | 0.390 | Colon Sigmoid |
|  | HLA-DQB2 | 6.22715e-07 | 0.357 | Colon Transverse |
|  | HLA-DQB2 | 1.71167e-06 | 0.385 | Esophagus Gastroesophageal Junction |
|  | HLA-DQB2 | 1.02869e-10 | 0.451 | Esophagus Muscularis |
|  | HLA-DQB2 | 4.18888e-09 | 0.451 | Heart Atrial Appendage |
|  | HLA-DQB2 | 3.16679e-09 | 0.446 | Heart Left Ventricle |
|  | HLA-DQB2 | 1.14005e-05 | 0.435 | Liver |
|  | HLA-DQB2 | 5.0786e-12 | 0.392 | Lung |
|  | HLA-DQB2 | 6.79672e-15 | 0.428 | Muscle Skeletal |
|  | HLA-DQB2 | 2.18307e-11 | 0.427 | Nerve Tibial |
|  | HLA-DQB2 | 5.87725e-07 | 0.517 | Ovary |
|  | HLA-DQB2 | 1.78529e-10 | 0.482 | Pancreas |
|  | HLA-DQB2 | 1.36418e-05 | 0.439 | Prostate |
|  | HLA-DQB2 | 8.19993e-06 | 0.395 | Spleen |
|  | HLA-DQB2 | 3.95699e-09 | 0.396 | Stomach |
|  | HLA-DQB2 | 6.65923e-06 | 0.403 | Testis |
|  | HLA-DQB2 | 3.46769e-12 | 0.386 | Thyroid |
|  | HLA-DQB2 | 7.01466e-21 | 0.443 | Whole Blood |
|  | HLA-DRB1 | 1.84236E-04 | -0.142 | Adipose Subcutaneous |
|  | HLA-DRB1 | 9.33506e-05 | -0.243 | Heart Atrial Appendage |
|  | HLA-DRB1 | 1.83177e-07 | -0.177 | Lung |
|  | HLA-DRB1 | 1.99514e-06 | -0.161 | Muscle Skeletal |
|  | HLA-DRB1 | 3.31646e-06 | -0.168 | Skin Not Sun Exposed Suprapubic |
|  | HLA-DRB1 | 2.73078e-10 | -0.210 | Skin Sun Exposed Lower leg |
|  | HLA-DRB1 | 1.00980E-04 | -0.270 | Testis |
|  | HLA-DRB1 | 7.30217e-07 | -0.183 | Thyroid |
|  | HLA-DRB5 | 3.56151e-07 | -0.293 | Adipose Subcutaneous |
|  | HLA-DRB5 | 9.65322e-05 | -0.248 | Adipose Visceral Omentum |
|  | HLA-DRB5 | 1.43764e-05 | -0.228 | Artery Tibial |
|  | HLA-DRB5 | 4.83585e-06 | -0.495 | Brain Anterior cingulate cortex BA24 |
|  | HLA-DRB5 | 5.27157e-05 | -0.231 | Esophagus Mucosa |
|  | HLA-DRB5 | 1.19312E-04 | -0.297 | Heart Left Ventricle |
|  | HLA-DRB5 | 8.42672e-07 | -0.286 | Lung |
|  | HLA-DRB5 | 2.92599e-07 | -0.269 | Muscle Skeletal |
|  | HLA-DRB5 | 6.05903e-06 | -0.265 | Skin Not Sun Exposed Suprapubic |
|  | HLA-DRB5 | 3.73328e-06 | -0.265 | Skin Sun Exposed Lower leg |
|  | HLA-DRB5 | 5.67873e-08 | -0.328 | Thyroid |
|  | HLA-DRB5 | 3.29195e-10 | -0.238 | Whole Blood |
|  | HLA-DRB6 | 8.75529e-20 | 0.563 | Adipose Subcutaneous |
|  | HLA-DRB6 | 5.02187e-22 | 0.608 | Adipose Visceral Omentum |
|  | HLA-DRB6 | 2.05733e-11 | 0.661 | Adrenal Gland |
|  | HLA-DRB6 | 1.14476e-17 | 0.505 | Artery Aorta |
|  | HLA-DRB6 | 2.07346e-12 | 0.607 | Artery Coronary |
|  | HLA-DRB6 | 7.13504e-21 | 0.418 | Artery Tibial |
|  | HLA-DRB6 | 2.00293e-08 | 0.599 | Brain Amygdala |
|  | HLA-DRB6 | 4.03585e-08 | 0.547 | Brain Anterior cingulate cortex BA24 |
|  | HLA-DRB6 | 3.24334e-06 | 0.447 | Brain Caudate basal ganglia |
|  | HLA-DRB6 | 1.62392e-08 | 0.602 | Brain Cerebellar Hemisphere |
|  | HLA-DRB6 | 2.58434e-09 | 0.594 | Brain Cerebellum |
|  | HLA-DRB6 | 5.31654e-09 | 0.597 | Brain Cortex |
|  | HLA-DRB6 | 2.23534e-08 | 0.459 | Brain Frontal Cortex BA9 |
|  | HLA-DRB6 | 5.41189e-10 | 0.636 | Brain Hippocampus |
|  | HLA-DRB6 | 1.36192e-08 | 0.606 | Brain Hypothalamus |
|  | HLA-DRB6 | 1.10384e-07 | 0.502 | Brain Nucleus accumbens basal ganglia |
|  | HLA-DRB6 | 5.62543e-09 | 0.684 | Brain Putamen basal ganglia |
|  | HLA-DRB6 | 1.7547e-07 | 0.609 | Brain Substantia nigra |
|  | HLA-DRB6 | 3.90405e-18 | 0.582 | Breast Mammary Tissue |
|  | HLA-DRB6 | 1.86167e-11 | 0.565 | Colon Sigmoid |
|  | HLA-DRB6 | 1.40176e-14 | 0.529 | Colon Transverse |
|  | HLA-DRB6 | 5.90737e-13 | 0.528 | Esophagus Gastroesophageal Junction |
|  | HLA-DRB6 | 6.90013e-22 | 0.536 | Esophagus Mucosa |
|  | HLA-DRB6 | 8.9424e-18 | 0.566 | Esophagus Muscularis |
|  | HLA-DRB6 | 9.12119e-18 | 0.640 | Heart Atrial Appendage |
|  | HLA-DRB6 | 1.1456e-17 | 0.589 | Heart Left Ventricle |
|  | HLA-DRB6 | 5.83867e-07 | 0.497 | Liver |
|  | HLA-DRB6 | 6.15321e-24 | 0.591 | Lung |
|  | HLA-DRB6 | 8.95755e-06 | 0.553 | Minor Salivary Gland |
|  | HLA-DRB6 | 2.88735e-24 | 0.543 | Muscle Skeletal |
|  | HLA-DRB6 | 2.82079e-24 | 0.599 | Nerve Tibial |
|  | HLA-DRB6 | 8.64784e-09 | 0.535 | Ovary |
|  | HLA-DRB6 | 7.37795e-15 | 0.550 | Pancreas |
|  | HLA-DRB6 | 9.00763e-11 | 0.528 | Pituitary |
|  | HLA-DRB6 | 9.64051e-11 | 0.626 | Prostate |
|  | HLA-DRB6 | 3.33531e-22 | 0.584 | Skin Not Sun Exposed Suprapubic |
|  | HLA-DRB6 | 3.88979e-27 | 0.591 | Skin Sun Exposed Lower leg |
|  | HLA-DRB6 | 1.41172e-06 | 0.480 | Small Intestine Terminal Ileum |
|  | HLA-DRB6 | 5.49006e-10 | 0.564 | Spleen |
|  | HLA-DRB6 | 6.00529e-18 | 0.481 | Stomach |
|  | HLA-DRB6 | 5.21608e-13 | 0.524 | Testis |
|  | HLA-DRB6 | 8.08883e-19 | 0.502 | Thyroid |
|  | HLA-DRB6 | 3.17599e-07 | 0.550 | Uterus |
|  | HLA-DRB6 | 1.3574e-08 | 0.615 | Vagina |
|  | HLA-DRB6 | 3.18571e-13 | 0.338 | Whole Blood |
|  | HLA-DRB9 | 5.19305e-07 | 0.332 | Adipose Visceral Omentum |
|  | HLA-DRB9 | 1.1061e-05 | 0.257 | Lung |
|  | HLA-DRB9 | 4.66935e-06 | 0.278 | Nerve Tibial |
|  | HLA-DRB9 | 6.51815e-05 | 0.239 | Thyroid |
|  | HLA-DRB9 | 1.49237E-04 | 0.176 | Whole Blood |
|  | NOTCH4 | 7.39294e-09 | -0.153 | Adipose Subcutaneous |
|  | NOTCH4 | 5.0312e-12 | -0.222 | Adipose Visceral Omentum |
|  | NOTCH4 | 6.58528e-06 | -0.364 | Brain Anterior cingulate cortex BA24 |
|  | NOTCH4 | 3.95968e-07 | -0.324 | Brain Cerebellum |
|  | NOTCH4 | 1.9475e-06 | -0.321 | Brain Cortex |
|  | NOTCH4 | 3.63809e-06 | -0.267 | Brain Frontal Cortex BA9 |
|  | NOTCH4 | 7.37929e-05 | -0.270 | Brain Nucleus accumbens basal ganglia |
|  | NOTCH4 | 1.47121e-05 | -0.107 | Breast Mammary Tissue |
|  | NOTCH4 | 1.92784e-05 | -0.169 | Esophagus Gastroesophageal Junction |
|  | NOTCH4 | 1.48469E-04 | -0.150 | Esophagus Mucosa |
|  | NOTCH4 | 1.24891e-05 | -0.168 | Esophagus Muscularis |
|  | NOTCH4 | 8.53567e-08 | -0.175 | Lung |
|  | NOTCH4 | 7.53769e-10 | -0.208 | Muscle Skeletal |
|  | NOTCH4 | 2.48403e-06 | -0.181 | Nerve Tibial |
|  | NOTCH4 | 7.08697e-05 | -0.213 | Pituitary |
|  | NOTCH4 | 6.80935e-09 | -0.170 | Skin Not Sun Exposed Suprapubic |
|  | NOTCH4 | 4.46184e-10 | -0.159 | Skin Sun Exposed Lower leg |
|  | NOTCH4 | 3.26176e-05 | -0.228 | Whole Blood |
|  | PSMB9 | 5.85883e-05 | 0.137 | Adipose Visceral Omentum |
|  | RNF5 | 1.44743e-05 | -0.202 | Adrenal Gland |
|  | RNF5 | 2.48501e-05 | -0.159 | Artery Aorta |
|  | RNF5 | 2.49582E-04 | -0.099 | Artery Tibial |
|  | RNF5 | 2.73924e-05 | -0.095 | Cells Cultured fibroblasts |
|  | RNF5 | 2.38412E-04 | -0.097 | Esophagus Muscularis |
|  | RNF5 | 7.74444e-08 | -0.113 | Muscle Skeletal |
|  | RNF5 | 3.09322e-06 | -0.125 | Skin Not Sun Exposed Suprapubic |
|  | RNF5 | 1.45037E-04 | -0.205 | Testis |
|  | RNF5 | 5.46489e-08 | -0.159 | Thyroid |
|  | SKIV2L | 7.26836e-07 | -0.185 | Artery Tibial |
|  | STK19B | 1.09335E-04 | -0.232 | Adipose Visceral Omentum |
|  | STK19B | 6.77706e-05 | -0.222 | Thyroid |
|  | TAP2 | 1.31088e-06 | -0.126 | Whole Blood |
|  | TNXA | 2.79356e-05 | -0.385 | Liver |
|  | XXbac-BPG154L12.5 | 2.18288e-07 | 0.402 | Testis |
| rs1264349 | ABHD16A | 6.42645e-05 | 0.285 | Heart Atrial Appendage |
|  | APOM | 4.36753e-06 | -0.211 | Muscle Skeletal |
|  | APOM | 3.66494E-04 | -0.177 | Skin Sun Exposed Lower leg |
|  | CCHCR1 | 4.26137e-18 | 0.539 | Adipose Subcutaneous |
|  | CCHCR1 | 4.6522e-12 | 0.368 | Adipose Visceral Omentum |
|  | CCHCR1 | 6.57833e-07 | 0.363 | Artery Aorta |
|  | CCHCR1 | 8.78088e-08 | 0.301 | Artery Tibial |
|  | CCHCR1 | 4.85545e-07 | 0.521 | Brain Cerebellar Hemisphere |
|  | CCHCR1 | 1.92158e-11 | 0.870 | Brain Cerebellum |
|  | CCHCR1 | 2.64167e-09 | 0.347 | Breast Mammary Tissue |
|  | CCHCR1 | 2.19522e-05 | 0.262 | Colon Sigmoid |
|  | CCHCR1 | 1.4046e-10 | 0.324 | Colon Transverse |
|  | CCHCR1 | 1.86169e-06 | 0.325 | Esophagus Gastroesophageal Junction |
|  | CCHCR1 | 7.62168e-10 | 0.335 | Esophagus Muscularis |
|  | CCHCR1 | 4.20709e-06 | 0.338 | Heart Atrial Appendage |
|  | CCHCR1 | 1.54696e-13 | 0.422 | Lung |
|  | CCHCR1 | 8.78836e-12 | 0.403 | Nerve Tibial |
|  | CCHCR1 | 5.16991e-05 | 0.414 | Pancreas |
|  | CCHCR1 | 2.98647e-07 | 0.335 | Skin Not Sun Exposed Suprapubic |
|  | CCHCR1 | 1.45177e-06 | 0.284 | Skin Sun Exposed Lower leg |
|  | CCHCR1 | 1.03381e-05 | 0.620 | Small Intestine Terminal Ileum |
|  | CCHCR1 | 4.84436e-06 | 0.487 | Spleen |
|  | CCHCR1 | 7.46505e-05 | 0.265 | Stomach |
|  | CCHCR1 | 1.88369E-04 | -0.337 | Testis |
|  | CCHCR1 | 8.98721e-16 | 0.441 | Thyroid |
|  | CCHCR1 | 1.79118e-12 | 0.327 | Whole Blood |
|  | CLIC1 | 6.53581e-05 | -0.074 | Whole Blood |
|  | DDAH2 | 1.98520E-04 | -0.112 | Muscle Skeletal |
|  | DDR1 | 1.15711E-04 | -0.252 | Esophagus Muscularis |
|  | DDR1-AS1 | 5.33049e-07 | 0.604 | Testis |
|  | FLOT1 | 1.24302e-07 | 0.211 | Adipose Subcutaneous |
|  | FLOT1 | 8.532e-19 | 0.361 | Adipose Visceral Omentum |
|  | FLOT1 | 3.43498e-11 | 0.296 | Artery Aorta |
|  | FLOT1 | 1.00232e-06 | 0.163 | Artery Tibial |
|  | FLOT1 | 1.28923e-06 | 0.369 | Brain Caudate basal ganglia |
|  | FLOT1 | 3.07294e-08 | 0.495 | Brain Cerebellar Hemisphere |
|  | FLOT1 | 1.30382e-13 | 0.750 | Brain Cerebellum |
|  | FLOT1 | 1.12264e-06 | 0.376 | Brain Cortex |
|  | FLOT1 | 2.57534e-05 | 0.307 | Brain Nucleus accumbens basal ganglia |
|  | FLOT1 | 2.96295e-08 | 0.215 | Breast Mammary Tissue |
|  | FLOT1 | 9.05424e-06 | 0.200 | Colon Transverse |
|  | FLOT1 | 2.01704e-07 | 0.285 | Esophagus Gastroesophageal Junction |
|  | FLOT1 | 2.17206e-08 | 0.246 | Esophagus Muscularis |
|  | FLOT1 | 5.69171e-16 | 0.271 | Lung |
|  | FLOT1 | 2.54975e-17 | 0.329 | Nerve Tibial |
|  | FLOT1 | 1.64081e-08 | 0.483 | Ovary |
|  | FLOT1 | 5.27743e-09 | 0.319 | Pancreas |
|  | FLOT1 | 1.26594e-14 | 0.517 | Pituitary |
|  | FLOT1 | 3.57732e-12 | 0.512 | Prostate |
|  | FLOT1 | 1.14461e-06 | 0.146 | Skin Not Sun Exposed Suprapubic |
|  | FLOT1 | 1.34105e-05 | 0.105 | Skin Sun Exposed Lower leg |
|  | FLOT1 | 3.00863e-08 | 0.476 | Small Intestine Terminal Ileum |
|  | FLOT1 | 2.95666e-08 | 0.324 | Spleen |
|  | FLOT1 | 2.05308e-07 | 0.267 | Stomach |
|  | FLOT1 | 8.60611e-30 | 0.484 | Thyroid |
|  | FLOT1 | 2.00581e-09 | 0.112 | Whole Blood |
|  | GPANK1 | 1.34076E-04 | -0.180 | Heart Left Ventricle |
|  | HCG17 | 1.95198E-04 | -0.388 | Adipose Subcutaneous |
|  | HCG17 | 3.51893E-04 | -0.338 | Artery Tibial |
|  | HCG17 | 3.42705e-07 | -0.740 | Brain Caudate basal ganglia |
|  | HCG17 | 2.88406e-05 | -0.658 | Brain Cortex |
|  | HCG17 | 1.35367e-05 | -0.673 | Brain Putamen basal ganglia |
|  | HCG18 | 8.70351e-05 | -0.165 | Whole Blood |
|  | HCG20 | 3.85912e-06 | -0.469 | Cells Cultured fibroblasts |
|  | HCG20 | 3.44931E-04 | -0.336 | Skin Sun Exposed Lower leg |
|  | HCG22 | 7.97545e-05 | -0.503 | Heart Atrial Appendage |
|  | HCG4B | 3.12688E-04 | 0.410 | Nerve Tibial |
|  | HCG4B | 3.89770E-04 | -0.366 | Skin Not Sun Exposed Suprapubic |
|  | HCG4P3 | 1.26699E-04 | 0.336 | Adipose Subcutaneous |
|  | HCG4P3 | 2.79644e-05 | 0.343 | Adipose Visceral Omentum |
|  | HCG4P3 | 1.83614E-04 | 0.340 | Artery Tibial |
|  | HCG4P3 | 1.19011E-04 | 0.599 | Brain Frontal Cortex BA9 |
|  | HCG4P3 | 4.77834e-06 | 0.399 | Cells Cultured fibroblasts |
|  | HCG4P3 | 4.90747e-05 | 0.363 | Esophagus Mucosa |
|  | HCG4P3 | 1.47014e-08 | 0.493 | Nerve Tibial |
|  | HCG4P3 | 2.61140E-04 | 0.297 | Skin Sun Exposed Lower leg |
|  | HCG4P5 | 2.88567E-04 | -0.289 | Skin Not Sun Exposed Suprapubic |
|  | HCG4P5 | 9.51544e-05 | -0.236 | Whole Blood |
|  | HCG4P7 | 8.01223e-05 | 0.408 | Artery Aorta |
|  | HCG4P7 | 6.46954e-05 | 0.436 | Esophagus Muscularis |
|  | HCG4P7 | 1.26748e-05 | 0.465 | Nerve Tibial |
|  | HCP5 | 2.38663E-04 | -0.206 | Artery Aorta |
|  | HCP5 | 2.22061E-04 | -0.170 | Skin Not Sun Exposed Suprapubic |
|  | HCP5 | 4.6025e-05 | -0.221 | Skin Sun Exposed Lower leg |
|  | HCP5 | 4.27135e-07 | -0.308 | Thyroid |
|  | HCP5B | 2.76239e-15 | 0.805 | Adipose Subcutaneous |
|  | HCP5B | 6.57154e-10 | 0.652 | Adipose Visceral Omentum |
|  | HCP5B | 1.13252e-06 | 0.758 | Adrenal Gland |
|  | HCP5B | 2.01066e-05 | 0.531 | Artery Aorta |
|  | HCP5B | 4.88452e-11 | 0.618 | Artery Tibial |
|  | HCP5B | 1.43289E-04 | 0.473 | Breast Mammary Tissue |
|  | HCP5B | 7.56766e-06 | 0.845 | Cells EBV-transformed lymphocytes |
|  | HCP5B | 2.28317E-04 | 0.500 | Colon Sigmoid |
|  | HCP5B | 4.55373e-10 | 0.732 | Colon Transverse |
|  | HCP5B | 3.17462e-08 | 0.669 | Esophagus Gastroesophageal Junction |
|  | HCP5B | 1.87464e-08 | 0.537 | Esophagus Mucosa |
|  | HCP5B | 1.10502e-06 | 0.523 | Esophagus Muscularis |
|  | HCP5B | 1.86294e-09 | 0.817 | Liver |
|  | HCP5B | 1.39952e-14 | 0.742 | Lung |
|  | HCP5B | 1.512e-06 | 0.410 | Muscle Skeletal |
|  | HCP5B | 2.84748e-14 | 0.772 | Nerve Tibial |
|  | HCP5B | 1.58712e-06 | 0.703 | Pituitary |
|  | HCP5B | 8.06871e-07 | 0.890 | Prostate |
|  | HCP5B | 4.26513e-06 | 0.459 | Skin Not Sun Exposed Suprapubic |
|  | HCP5B | 7.11866e-13 | 0.670 | Skin Sun Exposed Lower leg |
|  | HCP5B | 2.18113e-05 | 0.697 | Spleen |
|  | HCP5B | 1.60568E-04 | 0.518 | Stomach |
|  | HCP5B | 8.49132e-07 | 0.463 | Thyroid |
|  | HCP5B | 5.61541e-18 | 0.752 | Whole Blood |
|  | HLA-A | 1.31817e-05 | 0.424 | Artery Aorta |
|  | HLA-A | 2.10096E-04 | 0.201 | Artery Tibial |
|  | HLA-A | 2.49696e-05 | -0.266 | Muscle Skeletal |
|  | HLA-A | 9.31089e-07 | -0.275 | Skin Not Sun Exposed Suprapubic |
|  | HLA-A | 6.85191e-12 | -0.393 | Skin Sun Exposed Lower leg |
|  | HLA-A | 3.18891E-04 | -0.181 | Whole Blood |
|  | HLA-B | 2.65905e-06 | 0.473 | Testis |
|  | HLA-C | 2.01097e-06 | 0.359 | Adipose Visceral Omentum |
|  | HLA-C | 2.41535e-08 | 0.609 | Artery Aorta |
|  | HLA-C | 5.16316e-11 | 0.378 | Artery Tibial |
|  | HLA-C | 6.87865e-05 | 0.481 | Brain Cortex |
|  | HLA-C | 9.53692e-07 | 0.381 | Breast Mammary Tissue |
|  | HLA-C | 8.53828e-12 | 0.648 | Cells Cultured fibroblasts |
|  | HLA-C | 5.65088e-07 | 0.517 | Colon Sigmoid |
|  | HLA-C | 4.81318e-07 | 0.293 | Colon Transverse |
|  | HLA-C | 2.24815e-08 | 0.586 | Esophagus Gastroesophageal Junction |
|  | HLA-C | 8.96347e-10 | 0.459 | Esophagus Mucosa |
|  | HLA-C | 1.7466e-09 | 0.547 | Esophagus Muscularis |
|  | HLA-C | 8.40321e-05 | 0.541 | Liver |
|  | HLA-C | 5.95164e-07 | 0.374 | Lung |
|  | HLA-C | 1.09475e-19 | 0.654 | Muscle Skeletal |
|  | HLA-C | 1.51638e-05 | 0.329 | Nerve Tibial |
|  | HLA-C | 1.33793e-10 | 0.806 | Pancreas |
|  | HLA-C | 8.95937e-06 | 0.349 | Skin Not Sun Exposed Suprapubic |
|  | HLA-C | 5.05378e-07 | 0.368 | Skin Sun Exposed Lower leg |
|  | HLA-C | 1.63755e-06 | 0.416 | Stomach |
|  | HLA-C | 3.64502e-05 | 0.377 | Testis |
|  | HLA-C | 6.42243e-07 | 0.367 | Thyroid |
|  | HLA-C | 1.99858e-05 | 0.724 | Uterus |
|  | HLA-C | 2.29931e-05 | 0.207 | Whole Blood |
|  | HLA-H | 7.41965e-05 | -0.402 | Skin Not Sun Exposed Suprapubic |
|  | HLA-H | 4.81819e-06 | -0.426 | Skin Sun Exposed Lower leg |
|  | HLA-J | 7.78581e-07 | -0.481 | Adipose Subcutaneous |
|  | HLA-J | 2.11050E-04 | -0.401 | Artery Aorta |
|  | HLA-J | 1.41997e-05 | -0.358 | Artery Tibial |
|  | HLA-J | 9.75958e-05 | -0.533 | Brain Nucleus accumbens basal ganglia |
|  | HLA-J | 6.46734e-08 | -0.554 | Breast Mammary Tissue |
|  | HLA-J | 4.63375e-05 | -0.421 | Cells Cultured fibroblasts |
|  | HLA-J | 1.85672e-06 | -0.367 | Colon Transverse |
|  | HLA-J | 2.42959E-04 | -0.337 | Esophagus Mucosa |
|  | HLA-J | 1.25462e-05 | -0.372 | Heart Left Ventricle |
|  | HLA-J | 2.28917E-04 | -0.304 | Lung |
|  | HLA-J | 1.0844e-07 | -0.439 | Muscle Skeletal |
|  | HLA-J | 1.21005e-05 | -0.435 | Skin Not Sun Exposed Suprapubic |
|  | HLA-J | 1.61797e-11 | -0.619 | Skin Sun Exposed Lower leg |
|  | HLA-J | 5.88561e-06 | -0.312 | Thyroid |
|  | HLA-J | 1.80005e-05 | -0.351 | Whole Blood |
|  | HLA-K | 3.06695E-04 | -0.366 | Adipose Subcutaneous |
|  | HLA-K | 1.66148e-06 | -0.502 | Adipose Visceral Omentum |
|  | HLA-K | 9.64964e-05 | -0.487 | Artery Aorta |
|  | HLA-K | 2.61075e-07 | -0.511 | Artery Tibial |
|  | HLA-K | 8.25448e-07 | -0.870 | Brain Cerebellum |
|  | HLA-K | 1.58771e-05 | -0.740 | Brain Cortex |
|  | HLA-K | 6.08031e-05 | -0.664 | Brain Frontal Cortex BA9 |
|  | HLA-K | 5.43506e-06 | -0.718 | Brain Nucleus accumbens basal ganglia |
|  | HLA-K | 3.22286e-09 | -0.593 | Cells Cultured fibroblasts |
|  | HLA-K | 1.6829e-05 | -0.586 | Colon Sigmoid |
|  | HLA-K | 8.61116e-05 | -0.476 | Esophagus Gastroesophageal Junction |
|  | HLA-K | 2.39097e-08 | -0.602 | Esophagus Mucosa |
|  | HLA-K | 1.00178E-04 | -0.456 | Esophagus Muscularis |
|  | HLA-K | 6.63389e-09 | -0.559 | Lung |
|  | HLA-K | 1.67003e-09 | -0.491 | Muscle Skeletal |
|  | HLA-K | 1.06544e-06 | -0.556 | Nerve Tibial |
|  | HLA-K | 1.18173e-10 | -0.662 | Skin Not Sun Exposed Suprapubic |
|  | HLA-K | 2.67667e-13 | -0.703 | Skin Sun Exposed Lower leg |
|  | HLA-K | 1.47673E-04 | -0.505 | Stomach |
|  | HLA-K | 6.10972e-08 | -0.525 | Thyroid |
|  | HLA-K | 2.5224e-08 | -0.477 | Whole Blood |
|  | HLA-L | 1.39414E-04 | -0.300 | Muscle Skeletal |
|  | HLA-L | 1.19903e-09 | -0.563 | Whole Blood |
|  | HLA-S | 1.26358E-04 | -0.420 | Adipose Visceral Omentum |
|  | HLA-S | 2.36268e-06 | -0.451 | Artery Tibial |
|  | HLA-S | 2.26502e-06 | -0.508 | Lung |
|  | HLA-S | 1.34331E-04 | -0.369 | Skin Sun Exposed Lower leg |
|  | HLA-S | 3.07373e-06 | -0.445 | Thyroid |
|  | HLA-S | 1.23687e-05 | -0.397 | Whole Blood |
|  | HLA-T | 1.14076E-04 | -0.531 | Breast Mammary Tissue |
|  | HLA-T | 2.69029e-05 | -0.474 | Lung |
|  | HLA-T | 9.43039e-06 | -0.415 | Whole Blood |
|  | HLA-U | 2.94103E-04 | 0.424 | Adipose Visceral Omentum |
|  | HLA-U | 7.53678e-05 | 0.542 | Artery Aorta |
|  | HLA-U | 2.54161e-05 | 0.477 | Cells Cultured fibroblasts |
|  | HLA-U | 6.00692e-05 | 0.569 | Esophagus Gastroesophageal Junction |
|  | HLA-U | 3.20493E-04 | 0.460 | Esophagus Muscularis |
|  | HLA-W | 1.46148E-04 | -0.414 | Lung |
|  | HLA-W | 3.9226e-05 | -0.354 | Whole Blood |
|  | IER3 | 1.18509E-04 | 0.243 | Artery Aorta |
|  | IER3 | 3.22567E-04 | 0.141 | Cells Cultured fibroblasts |
|  | IER3 | 1.10787e-07 | 0.318 | Esophagus Mucosa |
|  | IER3 | 5.21096e-08 | 0.309 | Whole Blood |
|  | LINC00243 | 5.45784e-06 | 0.436 | Adipose Visceral Omentum |
|  | LINC00243 | 6.64121e-08 | 0.538 | Cells Cultured fibroblasts |
|  | LINC00243 | 5.69432e-07 | 0.497 | Esophagus Mucosa |
|  | LINC00243 | 5.89255e-09 | 0.529 | Lung |
|  | LINC00243 | 3.64902e-05 | 0.436 | Skin Not Sun Exposed Suprapubic |
|  | LINC00243 | 9.30517e-06 | 0.435 | Skin Sun Exposed Lower leg |
|  | LINC00243 | 2.92866e-23 | 0.589 | Whole Blood |
|  | LINC01149 | 1.92484E-04 | 0.197 | Testis |
|  | LY6G5B | 3.5741e-05 | 0.168 | Adipose Visceral Omentum |
|  | LY6G5B | 9.48929e-08 | 0.245 | Artery Aorta |
|  | LY6G5B | 1.07477E-04 | 0.127 | Artery Tibial |
|  | LY6G5B | 7.67299e-07 | 0.258 | Cells Cultured fibroblasts |
|  | LY6G5B | 1.01619e-05 | 0.217 | Heart Atrial Appendage |
|  | LY6G5B | 2.65933e-05 | 0.177 | Heart Left Ventricle |
|  | LY6G5B | 9.41081e-09 | 0.186 | Lung |
|  | LY6G5B | 1.41241E-04 | 0.139 | Muscle Skeletal |
|  | LY6G5B | 2.19196e-06 | 0.326 | Testis |
|  | LY6G5B | 5.99025e-07 | 0.192 | Thyroid |
|  | LY6G5B | 9.1833e-05 | 0.119 | Whole Blood |
|  | LY6G6C | 5.89938e-07 | 0.373 | Esophagus Mucosa |
|  | MICB | 4.54249e-05 | 0.345 | Adipose Subcutaneous |
|  | MICB | 1.44377e-08 | 0.391 | Adipose Visceral Omentum |
|  | MICB | 3.47503e-12 | 0.698 | Artery Aorta |
|  | MICB | 1.22279e-05 | 0.503 | Artery Coronary |
|  | MICB | 4.55526e-10 | 0.526 | Artery Tibial |
|  | MICB | 7.42307e-08 | 0.859 | Brain Cerebellar Hemisphere |
|  | MICB | 2.39444e-07 | 0.900 | Brain Cerebellum |
|  | MICB | 1.07131e-05 | 0.586 | Brain Cortex |
|  | MICB | 4.49877e-05 | 0.515 | Brain Frontal Cortex BA9 |
|  | MICB | 1.90138e-08 | 0.393 | Cells Cultured fibroblasts |
|  | MICB | 7.4885e-05 | 0.351 | Colon Sigmoid |
|  | MICB | 1.3358e-06 | 0.432 | Esophagus Gastroesophageal Junction |
|  | MICB | 4.15417e-12 | 0.573 | Esophagus Mucosa |
|  | MICB | 2.44375e-07 | 0.438 | Esophagus Muscularis |
|  | MICB | 3.32859e-05 | 0.317 | Muscle Skeletal |
|  | MICB | 3.98458e-06 | 0.448 | Nerve Tibial |
|  | MICB | 1.30764e-10 | 0.553 | Skin Not Sun Exposed Suprapubic |
|  | MICB | 3.41488e-09 | 0.448 | Skin Sun Exposed Lower leg |
|  | MICB | 1.53274e-05 | 0.367 | Stomach |
|  | MICB | 6.64515e-05 | 0.389 | Testis |
|  | MICB | 5.06936e-06 | 0.287 | Thyroid |
|  | MICB | 1.43866e-05 | 0.725 | Vagina |
|  | MIR6891 | 8.78848e-07 | -0.485 | Adipose Visceral Omentum |
|  | MIR6891 | 1.67015e-05 | -0.384 | Artery Tibial |
|  | MIR6891 | 4.06235e-05 | -0.478 | Colon Transverse |
|  | MIR6891 | 1.32081e-05 | -0.424 | Esophagus Mucosa |
|  | MIR6891 | 2.02669e-09 | -0.512 | Lung |
|  | MIR6891 | 4.65844e-06 | -0.510 | Nerve Tibial |
|  | MIR6891 | 2.16996e-05 | -0.612 | Pituitary |
|  | MIR6891 | 4.16996e-05 | -0.375 | Skin Not Sun Exposed Suprapubic |
|  | MIR6891 | 1.66302e-06 | -0.446 | Skin Sun Exposed Lower leg |
|  | MIR6891 | 6.23879e-05 | -0.357 | Thyroid |
|  | MIR6891 | 2.05848E-04 | -0.276 | Whole Blood |
|  | MSH5 | 2.34195e-05 | -0.158 | Thyroid |
|  | NRM | 2.81009E-04 | -0.220 | Esophagus Muscularis |
|  | POU5F1 | 1.28506e-08 | 0.504 | Adipose Subcutaneous |
|  | POU5F1 | 1.09085e-06 | 0.487 | Adipose Visceral Omentum |
|  | POU5F1 | 1.39378e-05 | 0.455 | Artery Aorta |
|  | POU5F1 | 6.31098e-08 | 0.464 | Artery Tibial |
|  | POU5F1 | 1.4801e-05 | 0.470 | Breast Mammary Tissue |
|  | POU5F1 | 1.55407E-04 | 0.316 | Thyroid |
|  | PPP1R18 | 2.9536e-05 | -0.165 | Adipose Visceral Omentum |
|  | PPP1R18 | 1.19939e-07 | -0.406 | Adrenal Gland |
|  | PPP1R18 | 4.79918e-19 | -0.466 | Artery Aorta |
|  | PPP1R18 | 3.78686e-14 | -0.302 | Artery Tibial |
|  | PPP1R18 | 1.40587e-09 | -0.209 | Cells Cultured fibroblasts |
|  | PPP1R18 | 9.89191e-06 | -0.286 | Esophagus Gastroesophageal Junction |
|  | PPP1R18 | 1.63085e-14 | -0.346 | Esophagus Muscularis |
|  | PPP1R18 | 1.44392e-06 | -0.173 | Lung |
|  | PPP1R18 | 8.29546e-08 | -0.234 | Nerve Tibial |
|  | PPP1R18 | 1.56087e-06 | -0.287 | Prostate |
|  | PPP1R18 | 2.30923e-06 | -0.178 | Thyroid |
|  | PSORS1C1 | 2.03782e-12 | -0.604 | Adipose Subcutaneous |
|  | PSORS1C1 | 6.4007e-08 | -0.450 | Adipose Visceral Omentum |
|  | PSORS1C1 | 2.30142e-05 | -0.637 | Adrenal Gland |
|  | PSORS1C1 | 1.70193e-09 | -0.668 | Artery Aorta |
|  | PSORS1C1 | 6.11054e-05 | -0.554 | Artery Coronary |
|  | PSORS1C1 | 9.59187e-11 | -0.604 | Artery Tibial |
|  | PSORS1C1 | 4.34972e-07 | -0.751 | Brain Hypothalamus |
|  | PSORS1C1 | 2.01998e-07 | -0.465 | Breast Mammary Tissue |
|  | PSORS1C1 | 6.89706e-09 | -0.420 | Cells Cultured fibroblasts |
|  | PSORS1C1 | 1.16065e-07 | -0.657 | Colon Sigmoid |
|  | PSORS1C1 | 6.24761e-09 | -0.644 | Colon Transverse |
|  | PSORS1C1 | 2.01338e-06 | -0.557 | Esophagus Gastroesophageal Junction |
|  | PSORS1C1 | 2.95927e-08 | -0.595 | Esophagus Mucosa |
|  | PSORS1C1 | 3.53946e-10 | -0.586 | Esophagus Muscularis |
|  | PSORS1C1 | 1.34135e-15 | -0.902 | Heart Atrial Appendage |
|  | PSORS1C1 | 1.66122e-14 | -0.905 | Heart Left Ventricle |
|  | PSORS1C1 | 1.14885e-09 | -0.575 | Lung |
|  | PSORS1C1 | 9.33446e-05 | -0.330 | Muscle Skeletal |
|  | PSORS1C1 | 1.57489e-10 | -0.689 | Nerve Tibial |
|  | PSORS1C1 | 5.14207e-05 | -0.542 | Pancreas |
|  | PSORS1C1 | 1.83094E-04 | -0.262 | Skin Sun Exposed Lower leg |
|  | PSORS1C1 | 2.71362e-05 | -0.642 | Small Intestine Terminal Ileum |
|  | PSORS1C1 | 1.42093e-05 | -0.709 | Spleen |
|  | PSORS1C1 | 1.08976e-06 | -0.534 | Stomach |
|  | PSORS1C1 | 1.8552e-25 | -0.974 | Thyroid |
|  | PSORS1C2 | 1.30014E-04 | -0.390 | Adipose Visceral Omentum |
|  | PSORS1C2 | 4.9805e-06 | -0.463 | Artery Tibial |
|  | PSORS1C2 | 6.24585e-05 | -0.424 | Lung |
|  | PSORS1C2 | 3.53878e-06 | -0.513 | Nerve Tibial |
|  | PSORS1C2 | 2.20699e-09 | -0.618 | Thyroid |
|  | RNF39 | 9.63089e-05 | 0.451 | Pancreas |
|  | RNU6-850P | 2.9199e-05 | -0.407 | Thyroid |
|  | SFTA2 | 2.223e-08 | 0.209 | Lung |
|  | TRIM26 | 1.26849E-04 | 0.375 | Brain Cerebellar Hemisphere |
|  | TRIM26 | 1.083e-05 | -0.199 | Testis |
|  | TRIM31 | 2.28543e-07 | -0.585 | Liver |
|  | TRIM31 | 1.49403e-09 | -0.476 | Lung |
|  | TRIM31 | 4.65996e-07 | -0.382 | Skin Not Sun Exposed Suprapubic |
|  | VARS2 | 3.42076e-08 | -0.410 | Adipose Subcutaneous |
|  | VARS2 | 8.05231e-05 | -0.354 | Artery Aorta |
|  | VARS2 | 1.64675e-08 | -0.358 | Artery Tibial |
|  | VARS2 | 1.78836e-05 | -0.519 | Brain Cortex |
|  | VARS2 | 2.15489e-06 | -0.495 | Brain Frontal Cortex BA9 |
|  | VARS2 | 2.49756e-06 | -0.272 | Cells Cultured fibroblasts |
|  | VARS2 | 3.8139e-05 | -0.509 | Cells EBV-transformed lymphocytes |
|  | VARS2 | 1.06254E-04 | -0.325 | Colon Sigmoid |
|  | VARS2 | 3.71792e-08 | -0.483 | Colon Transverse |
|  | VARS2 | 4.02231e-07 | -0.414 | Esophagus Gastroesophageal Junction |
|  | VARS2 | 1.30839E-04 | -0.270 | Esophagus Muscularis |
|  | VARS2 | 8.04753e-06 | -0.471 | Heart Atrial Appendage |
|  | VARS2 | 9.13854e-05 | -0.374 | Heart Left Ventricle |
|  | VARS2 | 3.48278e-07 | -0.344 | Lung |
|  | VARS2 | 1.47833e-07 | -0.423 | Nerve Tibial |
|  | VARS2 | 1.69682e-05 | -0.522 | Pancreas |
|  | VARS2 | 8.36339e-06 | -0.449 | Pituitary |
|  | VARS2 | 9.36037e-11 | -0.486 | Skin Not Sun Exposed Suprapubic |
|  | VARS2 | 7.63436e-17 | -0.651 | Skin Sun Exposed Lower leg |
|  | VARS2 | 1.84085e-06 | -0.335 | Thyroid |
|  | VARS2 | 2.56671e-11 | -0.251 | Whole Blood |
|  | WASF5P | 9.88459e-12 | 1.004 | Pancreas |
|  | WASF5P | 6.30362e-06 | 0.702 | Pituitary |
|  | ZNRD1 | 1.64641E-04 | -0.262 | Thyroid |
|  | ZNRD1ASP | 7.90075e-05 | -0.151 | Artery Tibial |
|  | ZNRD1ASP | 1.80905E-04 | -0.124 | Muscle Skeletal |

**Table S14. List of SNPs in the 99% credible set identified from fine-mapping analysis for each CPASSOC-identified locus shared between broad depression and PTB.**

| **Index SNP** | **99%**  **credible-set**  **SNPs** | **CHR** | **BP** | **R2** | ***P*** | **probNorm** | **cumSum** |
| --- | --- | --- | --- | --- | --- | --- | --- |
| rs13220522 | rs12173854 | 6 | 26371679 | 0.53 | 3.48E-12 | 2.20E-03 | 0.90 |
|  | rs12174602 | 6 | 26372827 | 0.53 | 3.16E-12 | 2.42E-03 | 0.86 |
|  | rs12174623 | 6 | 26373086 | 0.53 | 3.20E-12 | 2.39E-03 | 0.87 |
|  | rs12174631 | 6 | 26373150 | 0.53 | 3.23E-12 | 2.37E-03 | 0.88 |
|  | rs12174639 | 6 | 26373121 | 0.53 | 2.47E-12 | 3.07E-03 | 0.82 |
|  | rs12176129 | 6 | 26514940 | 0.46 | 8.48E-12 | 9.18E-04 | 0.99 |
|  | rs12176317 | 6 | 26372786 | 0.53 | 2.92E-12 | 2.61E-03 | 0.85 |
|  | rs13190739 | 6 | 26587373 | 0.72 | 5.50E-13 | 1.34E-02 | 0.72 |
|  | rs13195509 | 6 | 26463660 | 0.74 | 6.67E-12 | 1.16E-03 | 0.98 |
|  | rs13198716 | 6 | 26582035 | 0.90 | 7.47E-14 | 9.55E-02 | 0.33 |
|  | rs13201782 | 6 | 26651053 | 0.89 | 1.04E-13 | 6.88E-02 | 0.59 |
|  | rs13203358 | 6 | 26590578 | 0.49 | 1.40E-12 | 5.37E-03 | 0.78 |
|  | rs13208916 | 6 | 26601940 | 0.49 | 2.41E-12 | 3.15E-03 | 0.82 |
|  | rs13212985 | 6 | 26609989 | 0.72 | 8.31E-13 | 8.96E-03 | 0.75 |
|  | rs13220261 | 6 | 26499185 | 0.72 | 7.30E-12 | 1.06E-03 | 0.99 |
|  | rs13220495 | 6 | 26441640 | 0.94 | 3.24E-13 | 2.26E-02 | 0.69 |
|  | rs16891727 | 6 | 26488860 | 0.51 | 7.53E-12 | 1.03E-03 | 0.99 |
|  | rs16901784 | 6 | 26555433 | 0.46 | 6.67E-12 | 1.16E-03 | 0.98 |
|  | rs1978 | 6 | 26377573 | 0.53 | 3.53E-12 | 2.17E-03 | 0.90 |
|  | rs1979 | 6 | 26377591 | 0.53 | 5.31E-12 | 1.45E-03 | 0.97 |
|  | rs2073527 | 6 | 26374978 | 0.53 | 2.93E-12 | 2.60E-03 | 0.85 |
|  | rs2073529 | 6 | 26375159 | 0.53 | 2.72E-12 | 2.80E-03 | 0.84 |
|  | rs2073530 | 6 | 26375243 | 0.53 | 2.70E-12 | 2.82E-03 | 0.83 |
|  | rs2073531 | 6 | 26375256 | 0.53 | 2.57E-12 | 2.96E-03 | 0.83 |
|  | rs28360517 | 6 | 26602453 | 0.49 | 1.43E-12 | 5.25E-03 | 0.79 |
|  | rs28362606 | 6 | 26365586 | 0.53 | 4.80E-12 | 1.60E-03 | 0.96 |
|  | rs34104395 | 6 | 26478252 | 0.74 | 6.43E-12 | 1.20E-03 | 0.98 |
|  | rs34148261 | 6 | 26511744 | 0.72 | 4.03E-12 | 1.90E-03 | 0.95 |
|  | rs34229567 | 6 | 26588521 | 0.49 | 1.50E-12 | 5.04E-03 | 0.79 |
|  | rs34246779 | 6 | 26549212 | 0.72 | 2.69E-12 | 2.83E-03 | 0.83 |
|  | rs34436535 | 6 | 26370365 | 0.53 | 3.92E-12 | 1.95E-03 | 0.94 |
|  | rs34550936 | 6 | 26361985 | 0.53 | 3.71E-12 | 2.07E-03 | 0.91 |
|  | rs34605993 | 6 | 26454363 | 0.74 | 3.90E-12 | 1.97E-03 | 0.93 |
|  | rs34622023 | 6 | 26362119 | 0.53 | 3.80E-12 | 2.02E-03 | 0.92 |
|  | rs34781270 | 6 | 26593037 | 0.72 | 5.96E-13 | 1.24E-02 | 0.73 |
|  | rs34878490 | 6 | 26370572 | 0.53 | 3.71E-12 | 2.06E-03 | 0.91 |
|  | rs35076545 | 6 | 26603924 | 0.72 | 6.33E-13 | 1.17E-02 | 0.75 |
|  | rs35277236 | 6 | 26562269 | 0.72 | 1.88E-12 | 4.03E-03 | 0.81 |
|  | rs35304979 | 6 | 26356347 | 0.53 | 3.22E-12 | 2.37E-03 | 0.88 |
|  | rs35307327 | 6 | 26370474 | 0.53 | 3.92E-12 | 1.95E-03 | 0.94 |
|  | rs35400317 | 6 | 26593275 | 0.90 | 6.07E-14 | 1.17E-01 | 0.24 |
|  | rs35433030 | 6 | 26529890 | 0.72 | 3.61E-12 | 2.12E-03 | 0.91 |
|  | rs35555795 | 6 | 26509382 | 0.72 | 5.31E-12 | 1.45E-03 | 0.97 |
|  | rs35627490 | 6 | 26462104 | 0.74 | 4.66E-12 | 1.65E-03 | 0.96 |
|  | rs35680819 | 6 | 26455814 | 0.74 | 5.88E-12 | 1.31E-03 | 0.98 |
|  | rs35934643 | 6 | 26355094 | 0.53 | 3.21E-12 | 2.38E-03 | 0.87 |
|  | rs36033628 | 6 | 26456074 | 0.74 | 5.85E-12 | 1.32E-03 | 0.98 |
|  | rs36162392 | 6 | 26569135 | 0.72 | 1.05E-12 | 7.12E-03 | 0.77 |
|  | rs3734542 | 6 | 26468326 | 0.74 | 5.83E-12 | 1.33E-03 | 0.98 |
|  | rs3757138 | 6 | 26376103 | 0.53 | 2.97E-12 | 2.57E-03 | 0.86 |
|  | rs3823158 | 6 | 26463271 | 0.74 | 4.79E-12 | 1.61E-03 | 0.96 |
|  | rs41266839 | 6 | 26409890 | 0.94 | 3.80E-13 | 1.93E-02 | 0.71 |
|  | rs45527431 | 6 | 26599509 | 0.90 | 6.00E-14 | 1.19E-01 | 0.12 |
|  | rs4634439 | 6 | 26598004 | 0.49 | 1.55E-12 | 4.86E-03 | 0.80 |
|  | rs66488313 | 6 | 26631568 | 0.89 | 7.93E-14 | 9.01E-02 | 0.52 |
|  | rs66757203 | 6 | 26454956 | 0.74 | 2.82E-12 | 2.70E-03 | 0.84 |
|  | rs66823108 | 6 | 26377939 | 0.53 | 4.90E-12 | 1.57E-03 | 0.97 |
|  | rs66827971 | 6 | 26365766 | 0.53 | 3.89E-12 | 1.97E-03 | 0.93 |
|  | rs66941101 | 6 | 26530376 | 0.72 | 3.94E-12 | 1.95E-03 | 0.94 |
|  | rs67234939 | 6 | 26530419 | 0.46 | 8.37E-12 | 9.30E-04 | 0.99 |
|  | rs67509210 | 6 | 26354100 | 0.53 | 3.06E-12 | 2.49E-03 | 0.86 |
|  | rs67777156 | 6 | 26633711 | 0.89 | 7.51E-14 | 9.50E-02 | 0.43 |
|  | rs68072215 | 6 | 26377927 | 0.53 | 4.36E-12 | 1.76E-03 | 0.95 |
|  | rs6900665 | 6 | 26487169 | 0.52 | 7.61E-12 | 1.02E-03 | 0.99 |
|  | rs6920256 | 6 | 26537801 | 0.47 | 3.34E-12 | 2.29E-03 | 0.89 |
|  | rs71557332 | 6 | 26356853 | 0.53 | 3.29E-12 | 2.32E-03 | 0.89 |
|  | rs71557334 | 6 | 26365913 | 0.53 | 5.79E-12 | 1.33E-03 | 0.98 |
|  | rs72836482 | 6 | 26359241 | 0.53 | 3.76E-12 | 2.04E-03 | 0.92 |
|  | rs72841536 | 6 | 26378288 | 0.53 | 3.48E-12 | 2.20E-03 | 0.90 |
|  | rs72843784 | 6 | 26498758 | 0.90 | 1.34E-13 | 5.38E-02 | 0.64 |
|  | rs72844462 | 6 | 26563864 | 0.72 | 2.40E-12 | 3.17E-03 | 0.82 |
|  | rs72845428 | 6 | 26550144 | 0.72 | 2.65E-12 | 2.87E-03 | 0.83 |
|  | rs75782365 | 6 | 26408551 | 0.94 | 2.67E-13 | 2.73E-02 | 0.67 |
|  | rs9348709 | 6 | 26360311 | 0.53 | 3.86E-12 | 1.98E-03 | 0.93 |
|  | rs9348712 | 6 | 26366511 | 0.53 | 4.61E-12 | 1.67E-03 | 0.96 |
|  | rs9348716 | 6 | 26375658 | 0.51 | 4.16E-12 | 1.84E-03 | 0.95 |
|  | rs9348726 | 6 | 26605206 | 0.49 | 1.67E-12 | 4.52E-03 | 0.81 |
|  | rs9357006 | 6 | 26364628 | 0.53 | 3.98E-12 | 1.93E-03 | 0.94 |
|  | rs9358932 | 6 | 26362705 | 0.53 | 3.99E-12 | 1.92E-03 | 0.94 |
|  | rs9358934 | 6 | 26363755 | 0.53 | 3.46E-12 | 2.21E-03 | 0.90 |
|  | rs9358935 | 6 | 26369587 | 0.53 | 3.91E-12 | 1.96E-03 | 0.94 |
|  | rs9358936 | 6 | 26370657 | 0.53 | 3.83E-12 | 2.00E-03 | 0.93 |
|  | rs9358937 | 6 | 26374274 | 0.53 | 2.86E-12 | 2.66E-03 | 0.85 |
|  | rs9366653 | 6 | 26354247 | 0.53 | 3.23E-12 | 2.37E-03 | 0.88 |
|  | rs9366654 | 6 | 26377028 | 0.52 | 2.74E-12 | 2.78E-03 | 0.84 |
|  | rs9366655 | 6 | 26377385 | 0.53 | 3.15E-12 | 2.42E-03 | 0.86 |
|  | rs9379851 | 6 | 26354780 | 0.53 | 5.15E-12 | 1.50E-03 | 0.97 |
|  | rs9379852 | 6 | 26357278 | 0.53 | 3.30E-12 | 2.31E-03 | 0.89 |
|  | rs9379853 | 6 | 26357635 | 0.53 | 3.30E-12 | 2.31E-03 | 0.89 |
|  | rs9379854 | 6 | 26362854 | 0.53 | 3.81E-12 | 2.01E-03 | 0.92 |
|  | rs9379855 | 6 | 26364930 | 0.53 | 3.24E-12 | 2.35E-03 | 0.88 |
|  | rs9379856 | 6 | 26366836 | 0.53 | 4.41E-12 | 1.75E-03 | 0.96 |
|  | rs9379857 | 6 | 26367654 | 0.53 | 4.50E-12 | 1.71E-03 | 0.96 |
|  | rs9379858 | 6 | 26367689 | 0.53 | 5.04E-12 | 1.53E-03 | 0.97 |
|  | rs9379859 | 6 | 26369549 | 0.53 | 4.07E-12 | 1.88E-03 | 0.95 |
|  | rs9379863 | 6 | 26372427 | 0.53 | 3.16E-12 | 2.42E-03 | 0.87 |
|  | rs9379864 | 6 | 26372584 | 0.53 | 3.17E-12 | 2.41E-03 | 0.87 |
|  | rs9379871 | 6 | 26375854 | 0.52 | 2.84E-12 | 2.69E-03 | 0.85 |
|  | rs9379897 | 6 | 26601526 | 0.49 | 8.57E-13 | 8.70E-03 | 0.76 |
|  | rs9379899 | 6 | 26603015 | 0.49 | 1.36E-12 | 5.52E-03 | 0.78 |
|  | rs9379901 | 6 | 26603866 | 0.49 | 1.53E-12 | 4.92E-03 | 0.80 |
|  | rs9393703 | 6 | 26356951 | 0.53 | 3.28E-12 | 2.33E-03 | 0.88 |
|  | rs9393705 | 6 | 26361011 | 0.53 | 6.35E-12 | 1.22E-03 | 0.98 |
|  | rs9393706 | 6 | 26361500 | 0.53 | 3.80E-12 | 2.02E-03 | 0.92 |
|  | rs9393707 | 6 | 26362486 | 0.53 | 3.89E-12 | 1.97E-03 | 0.93 |
|  | rs9393708 | 6 | 26362643 | 0.53 | 3.51E-12 | 2.18E-03 | 0.90 |
|  | rs9393710 | 6 | 26367833 | 0.53 | 4.19E-12 | 1.83E-03 | 0.95 |
|  | rs9393711 | 6 | 26370659 | 0.53 | 4.96E-12 | 1.55E-03 | 0.97 |
|  | rs9393712 | 6 | 26371000 | 0.53 | 3.81E-12 | 2.01E-03 | 0.92 |
|  | rs9393713 | 6 | 26373678 | 0.53 | 3.56E-12 | 2.15E-03 | 0.91 |
|  | rs9393715 | 6 | 26375645 | 0.51 | 5.79E-12 | 1.33E-03 | 0.97 |
|  | rs9393735 | 6 | 26582327 | 0.43 | 7.59E-12 | 1.02E-03 | 0.99 |

**Table S15. List of SNPs in the 99% credible set identified from fine-mapping analysis for each CPASSOC-identified locus shared between major depression and PTB.**

| **Index SNP** | **99%**  **credible-set**  **SNPs** | **CHR** | **BP** | **R2** | ***P*** | **probNorm** | **cumSum** |
| --- | --- | --- | --- | --- | --- | --- | --- |
| rs149543464 | rs149543464 | 6 | 30400763 | 1.00 | 3.49E-10 | 5.33E-01 | 0.53 |
| rs57440165 | rs12173854 | 6 | 26371679 | 0.54 | 1.33E-07 | 8.18E-04 | 0.96 |
|  | rs12174602 | 6 | 26372827 | 0.54 | 1.39E-07 | 7.83E-04 | 0.96 |
|  | rs12174623 | 6 | 26373086 | 0.54 | 1.26E-07 | 8.66E-04 | 0.95 |
|  | rs12174631 | 6 | 26373150 | 0.54 | 1.19E-07 | 9.14E-04 | 0.95 |
|  | rs12174639 | 6 | 26373121 | 0.54 | 1.10E-07 | 9.81E-04 | 0.94 |
|  | rs12176317 | 6 | 26372786 | 0.54 | 1.28E-07 | 8.47E-04 | 0.95 |
|  | rs13190739 | 6 | 26587373 | 0.78 | 2.03E-07 | 5.45E-04 | 0.98 |
|  | rs13194053 | 6 | 27143883 | 0.42 | 9.17E-08 | 1.17E-03 | 0.93 |
|  | rs13195401 | 6 | 26463574 | 0.97 | 2.38E-09 | 4.08E-02 | 0.33 |
|  | rs13195402 | 6 | 26463575 | 0.97 | 3.81E-09 | 2.58E-02 | 0.53 |
|  | rs13198716 | 6 | 26582035 | 0.98 | 6.48E-09 | 1.54E-02 | 0.69 |
|  | rs13201782 | 6 | 26651053 | 1.00 | 2.55E-09 | 3.80E-02 | 0.45 |
|  | rs13207082 | 6 | 27251379 | 0.97 | 1.38E-08 | 7.39E-03 | 0.76 |
|  | rs13212985 | 6 | 26609989 | 0.78 | 2.33E-07 | 4.76E-04 | 0.99 |
|  | rs13217285 | 6 | 26999845 | 0.70 | 3.14E-07 | 3.57E-04 | 0.99 |
|  | rs13219181 | 6 | 27136225 | 0.42 | 1.29E-07 | 8.46E-04 | 0.95 |
|  | rs13220495 | 6 | 26441640 | 0.95 | 2.46E-08 | 4.21E-03 | 0.88 |
|  | rs190945449 | 6 | 26828359 | 0.80 | 1.32E-07 | 8.26E-04 | 0.96 |
|  | rs1977 | 6 | 26377546 | 0.53 | 1.14E-07 | 9.52E-04 | 0.94 |
|  | rs1978 | 6 | 26377573 | 0.53 | 1.40E-07 | 7.81E-04 | 0.96 |
|  | rs1979 | 6 | 26377591 | 0.53 | 1.63E-07 | 6.73E-04 | 0.97 |
|  | rs2073527 | 6 | 26374978 | 0.54 | 1.28E-07 | 8.48E-04 | 0.95 |
|  | rs2073529 | 6 | 26375159 | 0.53 | 1.17E-07 | 9.28E-04 | 0.94 |
|  | rs2073530 | 6 | 26375243 | 0.54 | 1.14E-07 | 9.53E-04 | 0.94 |
|  | rs2073531 | 6 | 26375256 | 0.54 | 1.14E-07 | 9.53E-04 | 0.94 |
|  | rs28360634 | 6 | 27332891 | 0.89 | 6.65E-08 | 1.60E-03 | 0.93 |
|  | rs28362606 | 6 | 26365586 | 0.53 | 1.88E-07 | 5.85E-04 | 0.97 |
|  | rs34388707 | 6 | 27050396 | 0.42 | 5.54E-08 | 1.91E-03 | 0.92 |
|  | rs34436535 | 6 | 26370365 | 0.54 | 1.49E-07 | 7.35E-04 | 0.97 |
|  | rs34546498 | 6 | 26961280 | 0.98 | 4.64E-09 | 2.12E-02 | 0.60 |
|  | rs34550936 | 6 | 26361985 | 0.54 | 2.03E-07 | 5.44E-04 | 0.98 |
|  | rs34569203 | 6 | 27153984 | 0.42 | 8.84E-08 | 1.22E-03 | 0.93 |
|  | rs34622023 | 6 | 26362119 | 0.54 | 2.29E-07 | 4.84E-04 | 0.98 |
|  | rs34781270 | 6 | 26593037 | 0.78 | 1.91E-07 | 5.77E-04 | 0.98 |
|  | rs34783558 | 6 | 27171700 | 0.42 | 2.90E-07 | 3.85E-04 | 0.99 |
|  | rs34816374 | 6 | 26949672 | 0.93 | 3.71E-09 | 2.64E-02 | 0.51 |
|  | rs34878490 | 6 | 26370572 | 0.54 | 1.54E-07 | 7.12E-04 | 0.97 |
|  | rs35076545 | 6 | 26603924 | 0.78 | 1.89E-07 | 5.82E-04 | 0.98 |
|  | rs35173303 | 6 | 26935379 | 0.93 | 4.86E-09 | 2.03E-02 | 0.64 |
|  | rs35304979 | 6 | 26356347 | 0.54 | 2.31E-07 | 4.81E-04 | 0.98 |
|  | rs35307327 | 6 | 26370474 | 0.54 | 1.47E-07 | 7.44E-04 | 0.96 |
|  | rs35400317 | 6 | 26593275 | 0.98 | 2.44E-09 | 3.97E-02 | 0.37 |
|  | rs35565446 | 6 | 27145341 | 0.97 | 1.06E-08 | 9.50E-03 | 0.73 |
|  | rs35741362 | 6 | 27007687 | 0.64 | 2.84E-07 | 3.93E-04 | 0.99 |
|  | rs35869525 | 6 | 26946687 | 0.93 | 4.76E-09 | 2.07E-02 | 0.62 |
|  | rs35909544 | 6 | 27163456 | 0.66 | 2.91E-07 | 3.85E-04 | 0.99 |
|  | rs35934643 | 6 | 26355094 | 0.54 | 2.29E-07 | 4.83E-04 | 0.98 |
|  | rs3757138 | 6 | 26376103 | 0.54 | 1.29E-07 | 8.45E-04 | 0.95 |
|  | rs41266839 | 6 | 26409890 | 0.95 | 3.11E-08 | 3.34E-03 | 0.90 |
|  | rs45527431 | 6 | 26599509 | 0.98 | 2.22E-09 | 4.36E-02 | 0.29 |
|  | rs55664827 | 6 | 26824920 | 0.80 | 1.07E-07 | 1.01E-03 | 0.94 |
|  | rs55834529 | 6 | 27072542 | 0.95 | 8.73E-09 | 1.15E-02 | 0.71 |
|  | rs56114371 | 6 | 27274834 | 0.97 | 8.18E-09 | 1.22E-02 | 0.70 |
|  | rs57440165 | 6 | 26843517 | 1.00 | 6.67E-10 | 1.41E-01 | 0.14 |
|  | rs66462181 | 6 | 27091661 | 0.97 | 1.33E-08 | 7.63E-03 | 0.74 |
|  | rs66488313 | 6 | 26631568 | 1.00 | 1.74E-09 | 5.51E-02 | 0.20 |
|  | rs66823108 | 6 | 26377939 | 0.53 | 1.38E-07 | 7.91E-04 | 0.96 |
|  | rs66827971 | 6 | 26365766 | 0.54 | 1.28E-07 | 8.51E-04 | 0.95 |
|  | rs66975207 | 6 | 26942146 | 1.00 | 2.50E-09 | 3.87E-02 | 0.41 |
|  | rs67092078 | 6 | 27054772 | 0.97 | 6.01E-09 | 1.65E-02 | 0.68 |
|  | rs67330695 | 6 | 27103654 | 0.42 | 1.42E-07 | 7.67E-04 | 0.96 |
|  | rs67509210 | 6 | 26354100 | 0.54 | 2.79E-07 | 4.00E-04 | 0.99 |
|  | rs67540232 | 6 | 27140866 | 0.42 | 1.31E-07 | 8.34E-04 | 0.95 |
|  | rs67777156 | 6 | 26633711 | 1.00 | 1.86E-09 | 5.19E-02 | 0.25 |
|  | rs68072215 | 6 | 26377927 | 0.53 | 1.29E-07 | 8.41E-04 | 0.95 |
|  | rs6904071 | 6 | 27047256 | 0.42 | 5.81E-08 | 1.83E-03 | 0.93 |
|  | rs6913660 | 6 | 27091425 | 0.42 | 1.34E-07 | 8.11E-04 | 0.96 |
|  | rs6914824 | 6 | 27139048 | 0.42 | 1.18E-07 | 9.22E-04 | 0.94 |
|  | rs71206067 | 6 | 26903379 | 0.98 | 3.08E-09 | 3.17E-02 | 0.48 |
|  | rs71537559 | 6 | 27309779 | 0.89 | 1.70E-07 | 6.46E-04 | 0.97 |
|  | rs71557332 | 6 | 26356853 | 0.54 | 2.39E-07 | 4.65E-04 | 0.99 |
|  | rs71557334 | 6 | 26365913 | 0.54 | 2.42E-07 | 4.59E-04 | 0.99 |
|  | rs71557378 | 6 | 26903585 | 0.93 | 4.64E-09 | 2.13E-02 | 0.58 |
|  | rs72836482 | 6 | 26359241 | 0.54 | 2.25E-07 | 4.93E-04 | 0.98 |
|  | rs72839445 | 6 | 27249686 | 0.97 | 1.34E-08 | 7.55E-03 | 0.75 |
|  | rs72839477 | 6 | 27327000 | 0.89 | 5.51E-08 | 1.92E-03 | 0.92 |
|  | rs72841536 | 6 | 26378288 | 0.53 | 2.40E-07 | 4.64E-04 | 0.99 |
|  | rs72842197 | 6 | 27067083 | 0.42 | 9.75E-08 | 1.11E-03 | 0.94 |
|  | rs72843784 | 6 | 26498758 | 0.98 | 4.90E-09 | 2.02E-02 | 0.66 |
|  | rs75782365 | 6 | 26408551 | 0.95 | 3.20E-08 | 3.26E-03 | 0.90 |
|  | rs77666565 | 6 | 26851415 | 0.80 | 1.23E-07 | 8.80E-04 | 0.95 |
|  | rs80264589 | 6 | 26927602 | 0.93 | 4.39E-09 | 2.24E-02 | 0.55 |
|  | rs926300 | 6 | 27059443 | 0.42 | 1.18E-07 | 9.20E-04 | 0.95 |
|  | rs9348709 | 6 | 26360311 | 0.54 | 2.21E-07 | 5.01E-04 | 0.98 |
|  | rs9348712 | 6 | 26366511 | 0.54 | 1.67E-07 | 6.57E-04 | 0.97 |
|  | rs9348716 | 6 | 26375658 | 0.51 | 1.48E-07 | 7.40E-04 | 0.96 |
|  | rs9357006 | 6 | 26364628 | 0.54 | 1.97E-07 | 5.60E-04 | 0.98 |
|  | rs9358932 | 6 | 26362705 | 0.54 | 2.37E-07 | 4.68E-04 | 0.99 |
|  | rs9358934 | 6 | 26363755 | 0.54 | 1.94E-07 | 5.69E-04 | 0.98 |
|  | rs9358935 | 6 | 26369587 | 0.54 | 1.52E-07 | 7.18E-04 | 0.97 |
|  | rs9358936 | 6 | 26370657 | 0.54 | 1.65E-07 | 6.65E-04 | 0.97 |
|  | rs9358937 | 6 | 26374274 | 0.54 | 1.15E-07 | 9.46E-04 | 0.94 |
|  | rs9366654 | 6 | 26377028 | 0.53 | 1.29E-07 | 8.41E-04 | 0.95 |
|  | rs9366655 | 6 | 26377385 | 0.53 | 1.18E-07 | 9.21E-04 | 0.94 |
|  | rs9379851 | 6 | 26354780 | 0.54 | 2.28E-07 | 4.86E-04 | 0.98 |
|  | rs9379852 | 6 | 26357278 | 0.54 | 2.28E-07 | 4.86E-04 | 0.98 |
|  | rs9379853 | 6 | 26357635 | 0.54 | 2.25E-07 | 4.92E-04 | 0.98 |
|  | rs9379854 | 6 | 26362854 | 0.54 | 2.11E-07 | 5.24E-04 | 0.98 |
|  | rs9379855 | 6 | 26364930 | 0.54 | 2.39E-07 | 4.65E-04 | 0.99 |
|  | rs9379856 | 6 | 26366836 | 0.54 | 1.72E-07 | 6.40E-04 | 0.97 |
|  | rs9379857 | 6 | 26367654 | 0.54 | 1.73E-07 | 6.37E-04 | 0.97 |
|  | rs9379858 | 6 | 26367689 | 0.54 | 1.84E-07 | 5.99E-04 | 0.97 |
|  | rs9379859 | 6 | 26369549 | 0.54 | 1.58E-07 | 6.95E-04 | 0.97 |
|  | rs9379863 | 6 | 26372427 | 0.54 | 1.37E-07 | 7.93E-04 | 0.96 |
|  | rs9379864 | 6 | 26372584 | 0.54 | 1.38E-07 | 7.89E-04 | 0.96 |
|  | rs9379871 | 6 | 26375854 | 0.53 | 8.66E-08 | 1.24E-03 | 0.93 |
|  | rs9393703 | 6 | 26356951 | 0.54 | 2.20E-07 | 5.04E-04 | 0.98 |
|  | rs9393705 | 6 | 26361011 | 0.54 | 2.51E-07 | 4.44E-04 | 0.99 |
|  | rs9393706 | 6 | 26361500 | 0.54 | 2.20E-07 | 5.04E-04 | 0.98 |
|  | rs9393707 | 6 | 26362486 | 0.54 | 2.14E-07 | 5.18E-04 | 0.98 |
|  | rs9393708 | 6 | 26362643 | 0.54 | 2.11E-07 | 5.25E-04 | 0.98 |
|  | rs9393710 | 6 | 26367833 | 0.54 | 1.58E-07 | 6.95E-04 | 0.97 |
|  | rs9393711 | 6 | 26370659 | 0.54 | 1.97E-07 | 5.59E-04 | 0.98 |
|  | rs9393712 | 6 | 26371000 | 0.54 | 1.50E-07 | 7.29E-04 | 0.97 |
|  | rs9393713 | 6 | 26373678 | 0.54 | 1.62E-07 | 6.76E-04 | 0.97 |
|  | rs9393715 | 6 | 26375645 | 0.51 | 1.32E-07 | 8.27E-04 | 0.96 |

**Table S16. List of SNPs in the 99% credible set identified from fine-mapping analysis for each CPASSOC-identified locus shared between bipolar disease and PTB**

| **Index SNP** | **99%  credible-set  SNPs** | **CHR** | **BP** | **R2** | **P** | **probNorm** | **cumSum** |
| --- | --- | --- | --- | --- | --- | --- | --- |
| rs1264349 | rs1049633 | 6 | 30867527 | 0.90 | 1.27E-10 | 7.54E-03 | 0.67 |
|  | rs1059612 | 6 | 30708955 | 0.86 | 1.75E-10 | 5.53E-03 | 0.77 |
|  | rs1110482 | 6 | 30803526 | 0.90 | 8.75E-11 | 1.09E-02 | 0.46 |
|  | rs1264304 | 6 | 30882415 | 0.90 | 1.39E-10 | 6.92E-03 | 0.69 |
|  | rs1264308 | 6 | 30879987 | 0.90 | 1.19E-10 | 8.05E-03 | 0.59 |
|  | rs1264310 | 6 | 30873605 | 0.89 | 1.25E-10 | 7.70E-03 | 0.63 |
|  | rs1264312 | 6 | 30872982 | 0.90 | 1.27E-10 | 7.58E-03 | 0.66 |
|  | rs1264313 | 6 | 30872783 | 0.90 | 1.26E-10 | 7.60E-03 | 0.65 |
|  | rs1264322 | 6 | 30857894 | 0.90 | 1.26E-10 | 7.60E-03 | 0.65 |
|  | rs1264324 | 6 | 30855211 | 0.90 | 1.82E-10 | 5.32E-03 | 0.78 |
|  | rs1264325 | 6 | 30851989 | 0.90 | 1.25E-10 | 7.70E-03 | 0.64 |
|  | rs1264326 | 6 | 30851909 | 0.90 | 1.39E-10 | 6.93E-03 | 0.68 |
|  | rs1264341 | 6 | 30802465 | 0.90 | 7.60E-11 | 1.25E-02 | 0.44 |
|  | rs1264347 | 6 | 30798697 | 0.92 | 4.24E-11 | 2.21E-02 | 0.26 |
|  | rs1264349 | 6 | 30796659 | 1.00 | 2.71E-11 | 3.42E-02 | 0.03 |
|  | rs1264350 | 6 | 30796545 | 0.80 | 3.75E-11 | 2.49E-02 | 0.14 |
|  | rs1264351 | 6 | 30792117 | 0.99 | 3.34E-11 | 2.79E-02 | 0.09 |
|  | rs1264353 | 6 | 30787762 | 0.86 | 1.67E-10 | 5.80E-03 | 0.75 |
|  | rs1264356 | 6 | 30784068 | 0.86 | 1.16E-10 | 8.23E-03 | 0.56 |
|  | rs1264361 | 6 | 30777498 | 0.86 | 1.66E-10 | 5.82E-03 | 0.75 |
|  | rs1264373 | 6 | 30769273 | 0.99 | 4.22E-11 | 2.22E-02 | 0.24 |
|  | rs1619179 | 6 | 31175946 | 0.69 | 1.58E-10 | 6.09E-03 | 0.73 |
|  | rs1634716 | 6 | 30969754 | 0.82 | 2.14E-10 | 4.53E-03 | 0.85 |
|  | rs1634721 | 6 | 30977680 | 0.82 | 1.85E-10 | 5.22E-03 | 0.79 |
|  | rs1634726 | 6 | 30985828 | 0.82 | 1.52E-10 | 6.33E-03 | 0.72 |
|  | rs1639108 | 6 | 31196554 | 0.68 | 2.42E-10 | 4.03E-03 | 0.87 |
|  | rs2233980 | 6 | 31079644 | 0.69 | 5.46E-11 | 1.73E-02 | 0.30 |
|  | rs2263298 | 6 | 30820373 | 0.92 | 1.59E-10 | 6.05E-03 | 0.73 |
|  | rs2517572 | 6 | 30842629 | 0.90 | 1.20E-10 | 8.02E-03 | 0.60 |
|  | rs2517578 | 6 | 30811265 | 0.90 | 1.19E-10 | 8.06E-03 | 0.58 |
|  | rs2524266 | 6 | 30780568 | 0.97 | 2.84E-11 | 3.27E-02 | 0.07 |
|  | rs2535328 | 6 | 30825994 | 0.90 | 1.46E-10 | 6.59E-03 | 0.71 |
|  | rs2535332 | 6 | 30813249 | 0.90 | 1.16E-10 | 8.25E-03 | 0.55 |
|  | rs2535334 | 6 | 30812996 | 0.90 | 1.17E-10 | 8.17E-03 | 0.57 |
|  | rs2535340 | 6 | 30838497 | 0.90 | 1.18E-10 | 8.11E-03 | 0.57 |
|  | rs2844656 | 6 | 30834331 | 0.90 | 1.15E-10 | 8.32E-03 | 0.54 |
|  | rs2844661 | 6 | 30821522 | 0.90 | 1.14E-10 | 8.36E-03 | 0.53 |
|  | rs3094024 | 6 | 30495860 | 0.85 | 2.55E-10 | 3.82E-03 | 0.88 |
|  | rs3094030 | 6 | 30353426 | 0.59 | 6.47E-10 | 1.54E-03 | 0.92 |
|  | rs3094031 | 6 | 30351844 | 0.59 | 6.14E-10 | 1.62E-03 | 0.91 |
|  | rs3094032 | 6 | 30351547 | 0.59 | 6.21E-10 | 1.60E-03 | 0.92 |
|  | rs3094034 | 6 | 30363351 | 0.78 | 1.84E-09 | 5.56E-04 | 0.99 |
|  | rs3094035 | 6 | 30363136 | 0.59 | 3.37E-10 | 2.91E-03 | 0.90 |
|  | rs3094036 | 6 | 30363085 | 0.78 | 1.70E-09 | 6.02E-04 | 0.99 |
|  | rs3094049 | 6 | 30359360 | 0.59 | 4.27E-10 | 2.31E-03 | 0.91 |
|  | rs3094050 | 6 | 30358591 | 0.58 | 4.07E-10 | 2.42E-03 | 0.90 |
|  | rs3094053 | 6 | 30355184 | 0.59 | 5.81E-10 | 1.71E-03 | 0.91 |
|  | rs3094057 | 6 | 30329966 | 0.78 | 1.77E-09 | 5.77E-04 | 0.99 |
|  | rs3094058 | 6 | 30327196 | 0.78 | 1.58E-09 | 6.46E-04 | 0.98 |
|  | rs3094059 | 6 | 30327194 | 0.78 | 1.56E-09 | 6.55E-04 | 0.98 |
|  | rs3094067 | 6 | 30299245 | 0.81 | 6.83E-11 | 1.38E-02 | 0.39 |
|  | rs3094088 | 6 | 30894965 | 0.90 | 1.22E-10 | 7.88E-03 | 0.61 |
|  | rs3094118 | 6 | 30732858 | 0.78 | 1.08E-09 | 9.37E-04 | 0.96 |
|  | rs3094125 | 6 | 30709357 | 0.84 | 2.76E-10 | 3.54E-03 | 0.89 |
|  | rs3094222 | 6 | 31081434 | 0.68 | 4.58E-11 | 2.05E-02 | 0.28 |
|  | rs3094671 | 6 | 31044463 | 0.69 | 5.90E-11 | 1.60E-02 | 0.31 |
|  | rs3094703 | 6 | 30358957 | 0.59 | 9.95E-10 | 1.01E-03 | 0.96 |
|  | rs3094704 | 6 | 30357294 | 0.77 | 1.57E-09 | 6.47E-04 | 0.98 |
|  | rs3094712 | 6 | 30351348 | 0.78 | 1.61E-09 | 6.33E-04 | 0.98 |
|  | rs3094717 | 6 | 30385746 | 0.82 | 1.06E-09 | 9.49E-04 | 0.96 |
|  | rs3095152 | 6 | 30899650 | 0.71 | 1.02E-10 | 9.34E-03 | 0.51 |
|  | rs3095153 | 6 | 30899195 | 0.71 | 1.03E-10 | 9.28E-03 | 0.52 |
|  | rs3095155 | 6 | 30899051 | 0.90 | 1.24E-10 | 7.71E-03 | 0.62 |
|  | rs3095276 | 6 | 30566407 | 0.85 | 1.56E-10 | 6.17E-03 | 0.72 |
|  | rs3095311 | 6 | 31051675 | 0.69 | 4.04E-11 | 2.31E-02 | 0.22 |
|  | rs3095326 | 6 | 30725841 | 0.88 | 2.35E-10 | 4.14E-03 | 0.87 |
|  | rs3095327 | 6 | 30699022 | 0.69 | 4.22E-10 | 2.34E-03 | 0.91 |
|  | rs3095328 | 6 | 30723781 | 0.90 | 1.85E-10 | 5.24E-03 | 0.78 |
|  | rs3095332 | 6 | 30722267 | 0.90 | 1.90E-10 | 5.10E-03 | 0.82 |
|  | rs3095333 | 6 | 30750925 | 0.81 | 1.23E-09 | 8.24E-04 | 0.97 |
|  | rs3095334 | 6 | 30742713 | 0.80 | 1.39E-09 | 7.29E-04 | 0.98 |
|  | rs3095336 | 6 | 30738446 | 0.80 | 8.98E-10 | 1.12E-03 | 0.94 |
|  | rs3129809 | 6 | 30335621 | 0.59 | 6.85E-10 | 1.46E-03 | 0.93 |
|  | rs3129810 | 6 | 30337141 | 0.78 | 1.93E-09 | 5.30E-04 | 0.99 |
|  | rs3129812 | 6 | 30337974 | 0.82 | 8.74E-10 | 1.15E-03 | 0.94 |
|  | rs3129815 | 6 | 30340528 | 0.78 | 1.86E-09 | 5.49E-04 | 0.99 |
|  | rs3129818 | 6 | 30342966 | 0.59 | 6.92E-10 | 1.44E-03 | 0.93 |
|  | rs3129820 | 6 | 30343569 | 0.59 | 7.28E-10 | 1.37E-03 | 0.94 |
|  | rs3129821 | 6 | 30344038 | 0.59 | 7.49E-10 | 1.34E-03 | 0.94 |
|  | rs3129822 | 6 | 30346208 | 0.59 | 6.48E-10 | 1.54E-03 | 0.92 |
|  | rs3129823 | 6 | 30347186 | 0.59 | 6.77E-10 | 1.47E-03 | 0.92 |
|  | rs3129972 | 6 | 30717258 | 0.87 | 2.59E-10 | 3.77E-03 | 0.88 |
|  | rs3129973 | 6 | 30721143 | 0.88 | 2.78E-10 | 3.52E-03 | 0.89 |
|  | rs3129974 | 6 | 30723169 | 0.90 | 1.89E-10 | 5.13E-03 | 0.81 |
|  | rs3129978 | 6 | 30746331 | 0.80 | 1.12E-09 | 8.99E-04 | 0.97 |
|  | rs3129980 | 6 | 30758371 | 0.80 | 9.12E-10 | 1.10E-03 | 0.95 |
|  | rs3129981 | 6 | 30758857 | 0.63 | 1.39E-09 | 7.33E-04 | 0.98 |
|  | rs3129982 | 6 | 30760190 | 0.80 | 9.61E-10 | 1.05E-03 | 0.95 |
|  | rs3129983 | 6 | 30760276 | 0.80 | 1.01E-09 | 9.99E-04 | 0.96 |
|  | rs3129984 | 6 | 30761572 | 0.94 | 2.14E-10 | 4.53E-03 | 0.84 |
|  | rs3129985 | 6 | 30762542 | 0.80 | 9.60E-10 | 1.05E-03 | 0.95 |
|  | rs3130117 | 6 | 30508956 | 0.85 | 2.39E-10 | 4.08E-03 | 0.87 |
|  | rs3130123 | 6 | 30352647 | 0.59 | 5.98E-10 | 1.67E-03 | 0.91 |
|  | rs3130125 | 6 | 30352875 | 0.59 | 6.07E-10 | 1.64E-03 | 0.91 |
|  | rs3130126 | 6 | 30353739 | 0.59 | 6.83E-10 | 1.46E-03 | 0.93 |
|  | rs3130129 | 6 | 30395196 | 0.80 | 1.02E-09 | 9.89E-04 | 0.96 |
|  | rs3130141 | 6 | 30432177 | 0.80 | 1.43E-09 | 7.11E-04 | 0.98 |
|  | rs3130247 | 6 | 30515043 | 0.85 | 1.68E-10 | 5.76E-03 | 0.76 |
|  | rs3130350 | 6 | 30327839 | 0.78 | 1.52E-09 | 6.69E-04 | 0.98 |
|  | rs3130351 | 6 | 30328192 | 0.78 | 2.01E-09 | 5.11E-04 | 0.99 |
|  | rs3130352 | 6 | 30328357 | 0.78 | 1.97E-09 | 5.20E-04 | 0.99 |
|  | rs3130355 | 6 | 30318327 | 0.81 | 1.20E-10 | 7.97E-03 | 0.61 |
|  | rs3130356 | 6 | 30336663 | 0.78 | 1.10E-09 | 9.19E-04 | 0.96 |
|  | rs3130363 | 6 | 30340827 | 0.59 | 7.21E-10 | 1.39E-03 | 0.93 |
|  | rs3130364 | 6 | 30319154 | 0.78 | 1.18E-09 | 8.61E-04 | 0.97 |
|  | rs3130365 | 6 | 30341390 | 0.78 | 1.84E-09 | 5.56E-04 | 0.99 |
|  | rs3130370 | 6 | 30345294 | 0.59 | 7.14E-10 | 1.40E-03 | 0.93 |
|  | rs3130372 | 6 | 30346009 | 0.59 | 6.83E-10 | 1.46E-03 | 0.92 |
|  | rs3130544 | 6 | 31058340 | 0.73 | 3.80E-11 | 2.46E-02 | 0.17 |
|  | rs3130557 | 6 | 31094703 | 0.69 | 6.72E-11 | 1.41E-02 | 0.37 |
|  | rs3130562 | 6 | 31100974 | 0.69 | 6.29E-11 | 1.50E-02 | 0.33 |
|  | rs3130574 | 6 | 31016550 | 0.73 | 7.57E-11 | 1.25E-02 | 0.42 |
|  | rs3130641 | 6 | 30764081 | 0.82 | 1.21E-09 | 8.36E-04 | 0.97 |
|  | rs3130658 | 6 | 30701092 | 0.84 | 1.88E-10 | 5.14E-03 | 0.81 |
|  | rs3130660 | 6 | 30706361 | 0.84 | 1.78E-10 | 5.42E-03 | 0.77 |
|  | rs3130661 | 6 | 30708695 | 0.84 | 2.15E-10 | 4.52E-03 | 0.85 |
|  | rs3130668 | 6 | 30743729 | 0.92 | 2.87E-10 | 3.41E-03 | 0.89 |
|  | rs3130669 | 6 | 30743739 | 0.80 | 1.50E-09 | 6.81E-04 | 0.98 |
|  | rs3130670 | 6 | 30743864 | 0.80 | 1.30E-09 | 7.78E-04 | 0.98 |
|  | rs3130673 | 6 | 30746519 | 0.80 | 1.22E-09 | 8.27E-04 | 0.97 |
|  | rs3130985 | 6 | 31085356 | 0.70 | 4.01E-11 | 2.33E-02 | 0.19 |
|  | rs3131041 | 6 | 30755067 | 0.81 | 1.07E-09 | 9.46E-04 | 0.96 |
|  | rs3131044 | 6 | 30758664 | 0.80 | 8.96E-10 | 1.12E-03 | 0.94 |
|  | rs3131045 | 6 | 30758848 | 0.80 | 7.30E-10 | 1.37E-03 | 0.94 |
|  | rs3131047 | 6 | 30759335 | 0.80 | 9.21E-10 | 1.09E-03 | 0.95 |
|  | rs3131048 | 6 | 30759426 | 0.80 | 9.37E-10 | 1.07E-03 | 0.95 |
|  | rs3131049 | 6 | 30759489 | 0.80 | 9.46E-10 | 1.06E-03 | 0.95 |
|  | rs3131050 | 6 | 30760025 | 0.80 | 1.01E-09 | 1.00E-03 | 0.96 |
|  | rs3131052 | 6 | 30760232 | 0.82 | 1.10E-09 | 9.18E-04 | 0.97 |
|  | rs3131054 | 6 | 30761132 | 0.80 | 1.17E-09 | 8.68E-04 | 0.97 |
|  | rs3131055 | 6 | 30761487 | 0.80 | 9.13E-10 | 1.10E-03 | 0.95 |
|  | rs3131058 | 6 | 30762238 | 0.80 | 9.60E-10 | 1.05E-03 | 0.95 |
|  | rs3131060 | 6 | 30763291 | 0.80 | 1.62E-09 | 6.31E-04 | 0.98 |
|  | rs3131111 | 6 | 30381596 | 0.80 | 1.24E-09 | 8.20E-04 | 0.97 |
|  | rs3131112 | 6 | 30447674 | 0.81 | 9.26E-10 | 1.09E-03 | 0.95 |
|  | rs3131781 | 6 | 30937732 | 0.82 | 3.64E-10 | 2.70E-03 | 0.90 |
|  | rs3131788 | 6 | 31024796 | 0.69 | 8.91E-11 | 1.07E-02 | 0.47 |
|  | rs3131920 | 6 | 31018908 | 0.73 | 7.36E-11 | 1.29E-02 | 0.41 |
|  | rs3131921 | 6 | 30907335 | 0.64 | 1.90E-09 | 5.40E-04 | 0.99 |
|  | rs3132510 | 6 | 31172151 | 0.69 | 8.31E-11 | 1.14E-02 | 0.45 |
|  | rs3132541 | 6 | 31098734 | 0.69 | 6.69E-11 | 1.41E-02 | 0.36 |
|  | rs3132577 | 6 | 30831195 | 0.90 | 1.15E-10 | 8.35E-03 | 0.53 |
|  | rs3132580 | 6 | 30920124 | 0.72 | 1.02E-10 | 9.37E-03 | 0.50 |
|  | rs3132581 | 6 | 30913458 | 0.72 | 9.86E-11 | 9.68E-03 | 0.49 |
|  | rs3132582 | 6 | 30689001 | 0.69 | 3.91E-10 | 2.52E-03 | 0.90 |
|  | rs3132583 | 6 | 30688575 | 0.84 | 1.87E-10 | 5.17E-03 | 0.80 |
|  | rs3132599 | 6 | 30748164 | 0.80 | 1.26E-09 | 8.03E-04 | 0.97 |
|  | rs3132600 | 6 | 30746367 | 0.92 | 2.35E-10 | 4.14E-03 | 0.86 |
|  | rs3132603 | 6 | 30744200 | 0.80 | 1.17E-09 | 8.68E-04 | 0.97 |
|  | rs3132605 | 6 | 30739972 | 0.80 | 1.46E-09 | 6.97E-04 | 0.98 |
|  | rs3132610 | 6 | 30544401 | 0.81 | 1.84E-09 | 5.57E-04 | 0.99 |
|  | rs3132615 | 6 | 30441303 | 0.80 | 1.27E-09 | 8.00E-04 | 0.98 |
|  | rs3132617 | 6 | 30378408 | 0.80 | 1.31E-09 | 7.73E-04 | 0.98 |
|  | rs3132619 | 6 | 30374211 | 0.79 | 7.86E-10 | 1.27E-03 | 0.94 |
|  | rs3132625 | 6 | 30347720 | 0.59 | 6.23E-10 | 1.60E-03 | 0.92 |
|  | rs3132627 | 6 | 30346760 | 0.81 | 1.26E-09 | 8.07E-04 | 0.97 |
|  | rs3132630 | 6 | 30345118 | 0.59 | 7.19E-10 | 1.39E-03 | 0.93 |
|  | rs3132631 | 6 | 30344645 | 0.59 | 6.91E-10 | 1.44E-03 | 0.93 |
|  | rs3132634 | 6 | 30343272 | 0.72 | 6.31E-10 | 1.58E-03 | 0.92 |
|  | rs3132645 | 6 | 30409249 | 0.81 | 8.32E-10 | 1.21E-03 | 0.94 |
|  | rs7750641 | 6 | 31129310 | 0.70 | 9.33E-11 | 1.02E-02 | 0.48 |
|  | rs886420 | 6 | 30879636 | 0.92 | 1.72E-10 | 5.61E-03 | 0.76 |
|  | rs886422 | 6 | 30864279 | 0.90 | 1.30E-10 | 7.39E-03 | 0.68 |
|  | rs886424 | 6 | 30782002 | 0.86 | 1.43E-10 | 6.71E-03 | 0.70 |
|  | rs886425 | 6 | 30781301 | 0.86 | 1.64E-10 | 5.88E-03 | 0.74 |
|  | rs9262126 | 6 | 30596421 | 0.86 | 1.87E-10 | 5.18E-03 | 0.79 |
|  | rs9262130 | 6 | 30603519 | 0.86 | 1.42E-10 | 6.78E-03 | 0.70 |
|  | rs9262132 | 6 | 30611350 | 0.85 | 1.90E-10 | 5.10E-03 | 0.82 |
|  | rs9262135 | 6 | 30618906 | 0.86 | 2.00E-10 | 4.85E-03 | 0.84 |
|  | rs9262141 | 6 | 30644137 | 0.86 | 1.91E-10 | 5.07E-03 | 0.83 |
|  | rs9262142 | 6 | 30650026 | 0.86 | 1.99E-10 | 4.88E-03 | 0.83 |
|  | rs9262143 | 6 | 30652781 | 0.86 | 2.29E-10 | 4.24E-03 | 0.86 |
|  | rs9262146 | 6 | 30656828 | 0.86 | 2.14E-10 | 4.53E-03 | 0.84 |
|  | rs9262200 | 6 | 30760725 | 0.80 | 1.00E-09 | 1.00E-03 | 0.96 |
|  | rs9262202 | 6 | 30760907 | 0.80 | 1.22E-09 | 8.31E-04 | 0.97 |
|  | rs9262203 | 6 | 30760968 | 0.80 | 1.07E-09 | 9.42E-04 | 0.96 |
|  | rs9262204 | 6 | 30761089 | 0.80 | 1.43E-09 | 7.13E-04 | 0.98 |
| rs3132948 | rs9273363 | 6 | 32626272 | 0.01 | 3.73E-11 | 2.50E-02 | 0.12 |
|  | rs9273364 | 6 | 32626302 | 0.01 | 7.29E-11 | 1.30E-02 | 0.40 |
|  | rs9273368 | 6 | 32626475 | 0.01 | 6.57E-11 | 1.44E-02 | 0.34 |

**Table S17.** The results of LDSC using the gender-specific GWAS of UKB generated by the Neale Lab.

| **Trait 1** | **Trait 2** | ***r_g_*** | **se** | ***P*** |
| --- | --- | --- | --- | --- |
| Preterm birth | Both-sex depression by Neale Lab | 0.190 | 0.082 | 0.020 |
| Preterm birth | Female-specific depression by Neale Lab | 0.193 | 0.101 | 0.056 |
| Preterm birth | Male-specific depression by Neale Lab | 0.184 | 0.124 | 0.136 |

**Table S18.** The Mendelian randomization result of the association between female depression and the risk of preterm birth.

| **Exposure** | **IVs** | **Outcome** | **Method** | **No.IVs** | **Beta** | **SE** | ***P*** |
| --- | --- | --- | --- | --- | --- | --- | --- |
| Depression | Women-specific IVs identified by Neale Lab | PTB | IVW | 2 | 2.92 | 1.62 | 0.07 |
